# Identification of the Genes of the Plant Pathogen *Pseudomonas syringae* MB03 Required for the Nematicidal Activity Against *Caenorhabditis elegans* Through an Integrated Approach

**DOI:** 10.3389/fmicb.2022.826962

**Published:** 2022-03-09

**Authors:** Muhammad Ali, Tong Gu, Xun Yu, Anum Bashir, Zhiyong Wang, Xiaowen Sun, Naeem Mahmood Ashraf, Lin Li

**Affiliations:** ^1^State Key Laboratory of Agricultural Microbiology, Huazhong Agricultural University, Wuhan, China; ^2^Department of Biotechnology, COMSATS University Islamabad, Abbottabad, Pakistan; ^3^Department of Biochemistry and Biotechnology, University of Gujrat, Gujrat, Pakistan

**Keywords:** *Pseudomonas syringae*, *Caenorhabditis elegans*, pathogenomics, transcriptomics, transposon mutant library, gut colonization, nematicidal activity

## Abstract

Nematicidal potential of the common plant pathogen *Pseudomonas syringae* has been recently identified against *Caenorhabditis elegans*. The current study was designed to investigate the detailed genetic mechanism of the bacterial pathogenicity by applying comparative genomics, transcriptomics, mutant library screening, and protein expression. Results showed that *P. syringae* strain MB03 could kill *C. elegans* in the liquid assay by gut colonization. The genome of *P. syringae* MB03 was sequenced and comparative analysis including multi locus sequence typing, and genome-to-genome distance placed MB03 in phylogroup II of *P. syringae*. Furthermore, comparative genomics of MB03 with nematicidal strains of *Pseudomonas aeruginosa* (PAO1 and PA14) predicted 115 potential virulence factors in MB03. However, genes for previously reported nematicidal metabolites, such as phenazine, pyochelin, and pyrrolnitrin, were found absent in the MB03 genome. Transcriptomics analysis showed that the growth phase of the pathogen considerably affected the expression of virulence factors, as genes for the flagellum, glutamate ABC transporter, *phoP*/*phoQ*, *fleS*/*fleR*, type VI secretion system, and serralysin were highly up-regulated when stationary phase MB03 cells interacted with *C. elegans.* Additionally, screening of a transposon insertion mutant library led to the identification of other nematicidal genes such as *acnA, gltP, oprD*, and *zapE*. Finally, the nematicidal activity of selected proteins was confirmed by heterologous expression in *Escherichia coli*.

## Introduction

*Caenorhabditis elegans* has been widely utilized to study host–pathogen interaction. Pathogenicity mechanisms of numerous bacterial species of *Bacillus* (Geng et al., 2016), *Burkholderia* (Lee et al., 2011; Paiva et al., 2013), *Pseudomonas* (Dubern et al., 2015; Nandi et al., 2015; Ali et al., 2016), *Salmonella* (Alegado et al., 2011), *Staphylococcus* (Begun et al., 2005; Irazoqui et al., 2010) and *Yersinia* (Darby et al., 2002; Atkinson et al., 2011) have been reported against *C. elegans*. Bacterial species have acquired different mechanisms to kill *C. elegans* such as slow killing due to infection and colonization (Ali et al., 2016), biofilm formation (Darby et al., 2002; Atkinson et al., 2011), fast killing due to diffusible metabolites (Kirienko et al., 2015; Nandi et al., 2015), and secretion of proteins (Luo et al., 2012; Geng et al., 2016; Zhang et al., 2016). Several species of the genus *Pseudomonas*, including *P. aeruginosa*, *P. chlororaphis*, *P. fluorescens, P. putida*, and *P. protegens* have been reported for their pathogenicity against animals (Feinbaum et al., 2012; Burlinson et al., 2013; Fernandez et al., 2015; Wei et al., 2017). Among these species, *P. aeruginosa* PA14 has been reported as multi-host pathogen, capable of infecting nematodes, *Drosophila melanogaster*, and plants (Plotnikova et al., 2000; Kim et al., 2008). The interaction of *P. aeruginosa* with *C. elegans* has been well-characterized (Cezairliyan et al., 2013; Kirienko et al., 2013). The killing of *C. elegans* by the strains of *P. aeruginosa* has been attributed due to various mechanisms such as lethal paralysis, agar-based fast killing, liquid killing, and red death, along with gut colonization (Darby et al., 1999; Mahajan-Miklos et al., 1999; Tan et al., 1999b; Gallagher and Manoil, 2001; Zaborin et al., 2009; Cezairliyan et al., 2013; Kirienko et al., 2013, 2015; Ray et al., 2015). It has been established that change in the physical form of the killing assay (from agar-based killing to liquid-based killing) altered the killing mechanism; for instance, agar-based killing was mediated by the phenazines (Cezairliyan et al., 2013) and gut colonization (Tan et al., 1999a), whereas liquid killing was facilitated by pyoverdine (Kirienko et al., 2013).

*Pseudomonas syringae* is an important plant pathogen that causes foliar necroses in host plants and a hypersensitive reaction in non-hosts. Depending upon host range and host-pathogen interaction, this bacterial species was sub-divided into 50 pathovars (Hirano and Upper, 2000). More recently the species has been further divided into 60 pathovars (Gomila et al., 2017) and 13 phylogroups (Berge et al., 2014). The strains of *P. syringae* have been isolated from various agricultural and other environmental sites preferably in proximity to water cycle (Morris et al., 2007, 2008). Among 13 phylogroups, most of the strains in the phylogroup I were found from the damaged tissues of the plants. The phylogroup I also contained strains isolated from some other environmental samples (non-agricultural). The phyogroup II was the most diverse group as it contained strains from all known habitats (Berge et al., 2014). The released genomes of the different *P. syringae* pathovars, such as *P. syringae* pv. *syringae* B728a (Feil et al., 2005), *P. syringae* pv. *tomato* DC3000 (Buell et al., 2003), *P. syringae* CC1557 (Hockett et al., 2014), and *P. syringae* pv. *syringae* HS191 (Ravindran et al., 2015), have shown notable variations in terms of number and function of genes related to the bacterial pathogenicity. These strains not only have the common virulence factors but also have host-specific ones (O’Brien et al., 2011).

*Pseudomonas syringae* has been generally annotated as the “most notorious plant pathogen” (Mansfield et al., 2012), however, little information is available regarding its interaction with *C. elegans* (Ali et al., 2016; Dorati et al., 2018; Manan et al., 2018; Bashir et al., 2020). Previous attempts to investigate its pathogenic potential against *C. elegans* showed that the strain B728a and DC3000 did not possess nematicidal activity on NGM medium (Burlinson et al., 2013). The assay conditions are among the major factors that shape the outcome of the host–pathogen interaction (Burlinson et al., 2013). On these grounds, the pathogenicity of *P. syringae* MB03 against *C. elegans* was re-investigated under various assay conditions, and an obvious shift in the bacterial behavior was observed (from non-pathogenic to pathogenic) (Ali et al., 2016). Moreover, we have previously investigated the pathogenic behavior of *P. syringae* MB03 under nutrient-rich conditions and found that on PG medium, *P. syringae* MB03 could colonize the gut of *C. elegans* (Ali et al., 2016). In contrast, the killing of *C. elegans* by *P. aeruginosa* on PG medium (Cezairliyan et al., 2013) and in a liquid assay (Kirienko et al., 2013) was mediated by toxin secretion, rather than by gut colonization. Recently, Nif3-family protein from *P. syringae* MB03 was predicted as nematicidal factor and the purified protein was able to kill *C. elegans* and *Meloidogyne incognita* (Manan et al., 2018). These observations raised the possibility that *P. syringae* MB03 might be capable of killing nematodes because of multifactor mechanism.

This study was designed to investigate the key virulence factors of the *P. syringae* MB03 required for pathogenicity against *C. elegans* in the liquid killing assay. Comparative genomics, transcriptomics, and transposon insertion mutant library screening were performed to predict and identify key virulence factors. Further, the selected nematicidal genes were heterologously expressed in *Escherichia coli* to evaluate their toxicity. Collectively from these experiments, various species-common and strain-specific novel virulence factors belonging to *P. syringae* were identified.

## Materials and Methods

### Strains and Growth Conditions

A previously isolated laboratory isolate *P. syringae* MB03 (Li et al., 2012) was used to study bacterial pathogenicity against *C. elegans*. This strain has been deposited to the China Culture Collection with accession number CCTCC M2014114. *P. syringae* MB03 was cultured at 28°C and *E. coli* (DH5α, BL21, TOP10) strains were cultured at 37°C for routine growth. The *C. elegans* wild-type N2 (Bristol) was used as a model host in all bioassays. *C. elegans* N2 wildtype was obtained from *Caenorhabditis* Genetics Center College of Biological Sciences, University of Minnesota, United States. The worms were maintained on NGM medium having *E. coli* OP50 as food source at 20°C (Stiernagle, 2006). Synchronization of the population of worms was performed as reported previously (Stiernagle, 2006). The synchronized population of L4 larval stage worms was used in all assays.

### Nematode Killing Assay

*Pseudomonas syringae* MB03 was grown overnight in LB medium. Log phase bacterial cells were washed with M9 buffer and appropriate dilutions were made in S medium (Stiernagle, 2006). The assay was carried out in a 96 well plate. Each well contained 150 μl of bacterial cell suspension, 5 μl of 8 mM 5-fluorodeoxyuridine (FUdR, 0.2 mM final concentration), 40 μl S medium, and 30–40 *C. elegans* wild-type L4 stage.

### Plant Infection Assay

Fresh seedlings of wheat plants were used in the infection assay as previously reported (Uppalapati et al., 2008). Briefly, the bacterial strain was grown overnight in LB medium, and log-phase cells were harvested. Wheat plants were grown under optimum environmental conditions and healthy leaves of approximately similar size were selected for infection assay. Leaves were surface sterilized with 1% sodium hypochlorite solution. After rinsing with sterilized water, leaves were dipped into 100 ml of the bacterial suspension of *P. syringae* MB03 with a cell density of 0.05_OD600_. Leaves were placed on wet plates and sealed to avoid contamination. For the determination of infection, leaves were regularly observed for the appearance of lesions.

### Gut Colonization

To express red fluorescent protein (RFP), the plasmid pMCh-23 (Berry et al., 2012) was introduced into *P. syringae* MB03. Fluorescent protein-expressing *P. syringae* cells were grown overnight under optimum conditions, repeatedly washed with S medium and appropriate cell dilutions were prepared. L4 synchronized worms were washed with M9 buffer. The assay was performed in a 96 well plate, and each well contained 150 μl of RFP expressing *P. syringae* cell suspension, 5 μl of 8 mM 5-fluorodeoxyuridine (FUdR, 0.2-mM final concentration), 40 μl S medium, and 30–40 L4 worms.

### Genome Sequencing

To increase the understanding of the virulence mechanism of this bacterial strain, whole-genome shotgun sequencing of *P. syringae* MB03 was performed using Illumina technology. Quality trimming of 150-nucleotide (nt) paired-end reads was produced from a 500-bp genomic library (5.7 Mb, 175-fold coverage). For *de novo* assembly of the genome, SOAPdenovo software was used. Open reading frame calling and annotation was performed by using Glimmer software (Delcher et al., 2007). Blastall software was used to assign putative roles to proteins. The KEGG automatic annotation server (KAAS) was used to determine metabolic pathways (Kanehisa et al., 2016). The analysis generated 76 contigs, where the largest contig size was 549643 bp. The genome sequence was deposited at DDBJ/EMBL/GenBank under accession LAGV00000000. The version described in this article is version LAGV01000000. The genome sequence was also deposited to Integrated Microbial Genome (Genome submission ID 60303).

### Phylogeny and Comparative Genomics

Multi loci sequence analysis (MLSA) using seven housekeeping genes including *gyrB, gapA, fruK, pgi, rpoD, anB*, and *gltA* was performed to further classify *P. syringae* MB03 as reported previously (Sarkar and Guttman, 2004). The *P. syringae* MB03 genome was compared with other strains of *P. syringae* with the help of Mauve (Darling et al., 2004). GC skew was generated by submitting the draft genome of *P. syringae* MB03 to the CGView Server (Grant and Stothard, 2008). To distribute genes of *P. syringae* MB03 into auxiliary and core genome, the genome of *P. syringae* MB03 was compared with the previously determined core genome of *P. syringae* species (Baltrus et al., 2011). For this purpose, *in silico* genome subtraction was performed using mGenomeSubtractor with default settings (*H* value > 0.64) (Shao et al., 2010). The EDGAR online tool was used to determine the strain-specific genes (Blom et al., 2009). In addition, the amino acid identity matrix and genome-to-genome distance were also generated at EDGAR. Genomic islands were predicted by IslandViewer3 (Dhillon et al., 2013). Among the genome sequences used in this study for the comparison with *P. syringae* MB03, complete genome sequences of strains *P. syringae* B728a, CC1557, DC3000, HS191 are available at NCBI. In case of strains B64 and SM, only draft genome sequences are available at NCBI.

### Pathogenomic Analysis

To identify nematicidal proteins and virulence factors, protein databases were searched for nematicidal proteins, and their homologs were determined in *P. syringae* MB03 genome. Previously, the nematicidal proteins of *P. aeruginosa* strain PA14 and strain PAO1 have been identified by screening the transposon insertion mutant libraries (Feinbaum et al., 2012; Dubern et al., 2015). The protein sequences of these virulence factors were used to identify their homologs in *P. syringae* MB03. Moreover, amino acid sequences of virulence factors of *P. syringae* pv. *syringae* B728a, *P. syringae* pv. *tomato* DC3000, *P. aeruginosa* PA14 and *P. aeruginosa* PA01 were obtained from the virulence factor database (VFDB) (Chen et al., 2012) and their homologs were also identified in *P. syringae* MB03 by applying BLASTp. Sequences of type III secretion system effectors were obtained from *Pseudomonas syringae* Genome Resources Home Page^1^ and again BLASTp was used to identify the presence of genes of effector proteins in the genome *P. syringae* MB03. To investigate insertion or deletion of genomic sequences, the mauve suite was used and the genome of *P. syringae* MB03 was compared with the previously reported genomes of *P. syringae* pv. *syringae* B728a, *P. syringae* pv. *syringae* HS191, *P. syringae* pv. *syringae* B64, and *P. syringae* pv. *syringae* SM.

### Bioinformatics Analysis of Hypothetical Proteins and Transcriptional Regulators

The sequences of selected hypothetical proteins of *P. syringae* MB03 were retrieved from the NCBI database. Physiochemical properties, including molecular weight, aliphatic index, stability, and isoelectric points of hypothetical proteins, were identified by ProtParam server (Gasteiger et al., 2005). To explore the sub-cellular localization of hypothetical proteins, three different servers SignalP 4.1 (Petersen et al., 2011), CELLO (Yu et al., 2004), and TMHMM 2.0 (Krogh et al., 2001) were used. SignalP 4.1 is designed to identify a leader sequence at the N-terminus of a protein to provide an idea about the localization of the protein in the cell. Similarly, CELLO identifies the localization of proteins by support vector mechanics (SVM) based on the *n*-peptide composition. Conversely, TMHMM predicts the localization of the protein in the membrane by determining the transmembrane helical structures in the protein. Virulence of hypothetical proteins was predicted using the VICMpred server (Saha and Raghava, 2006). HPIDB 2.0 (Ammari et al., 2016) was used to predict hypothetical proteins, which are potentially involved in host-pathogen interaction. HPIDB is a database of experimentally characterized proteins, which are involved in host–pathogen interactions. Sequence homology-based search in HPIDB was used to predict proteins involved in host–pathogen interaction. To elucidate the 3D structures of hypothetical proteins suspected to be involved in host-pathogen interaction, I-TASSER (Yang et al., 2015) server was employed. Predicted 3D models for each protein by the server were further sorted based on the *C*-score and refined using the 3Drefine algorithm (Bhattacharya et al., 2016). Finally, transcription regulator binding sites were predicted by using CollecTF (Kılıç et al., 2016) using a homology based method, and their target genes were predicted.

### RNA Sequencing and Transcriptional Analysis

RNA sequencing of the *P. syringae* MB03 cells was performed to determine the change in the bacterial gene expression during the host–pathogen interactions. Interaction of host-pathogen was studied during two different growth phases of bacterial culture [(I) exponential phase and (II) stationary phase]. For this purpose, a single pure colony of *P. syringae* MB03 was cultured in LB broth. The overnight culture was used to further inoculate two different groups (namely, exponential phase group and stationary phase group) which were incubated at 28°C with shaking at 160 rpm for 12 h. Bacterial cells were harvested by centrifugation and washed repeatedly with S buffer. Finally, pre-fasting L4 *C. elegans* were exposed to bacterial cells in NGM broth for 5 h. After a specified time of interaction, worms were removed by centrifugation and washed repeatedly to obtain bacterial cells. This resulted in the fabrication of the exponential phase group. The pellets of bacterial cells were used to extract total RNA (*P. syringae* MB03 exposed to *C. elegans* as treatment and *P. syringae* MB03 incubated without *C. elegans* as control). For the stationary phase group, the entire sample treatment was the same, except the bacterial cells were grown for 24 h so that the cells might enter the stationary phase of growth. After 24 h growth, bacterial cells were exposed to pre-fasting L4 worms for 5 h. Worms were removed after a specified time interval. The bacterial cell pellet was obtained by repeated washing of worms. A bacterial cell pallet was used to isolate total RNA from the stationary phase bacterial cells for transcriptomics (*P. syringae* MB03 exposed to *C. elegans* as treatment and *P. syringae* MB03 incubated without *C. elegans* as control).

The overall methodology for transcriptomics was followed as described previously (Abbasi et al., 2021). Cellular RNA was isolated by Trizol method (Sigma-Aldrich, St. Louis, MO, United States). RNA samples were run on agarose gel for qualitative analysis. Further Qubit fluorometer (Thermo Fisher Scientific, Waltham, MA, United States) was used to quantify RNA samples and Agilent 2100 Bioanalyzer (Agilent, Santa Clara, CA, United States) was used to check integrity of the samples. Before library construction, rRNA was removed using Illumina rRNA depletion kit. Random hexamers were used for the preparation of cDNA. After RNA end repair and tail addition, sequencing adapters were connected. PCR enrichment was performed to obtain final cDNA library. Finally, HiSeq/MiSeq sequencing was performed. Clean reads were obtained by removing reads containing adapter sequence and low-quality reads. Clean reads were mapped with the genome sequence of *P. syringae* MB03 and with reference genomes including *P. syringae* B728a, *P. syringae* DC3000 using Bowtie 2 (Langmead and Salzberg, 2012). The enrichment analysis of GO and KEGG pathways was performed by using webserver for gene ontology (GO) and KEGG database.

### Construction and Screening of the Transposon Insertion Mutant Library

For the construction of transposon insertion mutant library, donor *E. coli* MB266 (S17-λpir harboring pUT mini-Tn5 Km2) and recipient cells (*P. syringae* MB03) were grown overnight in LB at 37 and 28°C, respectively. One ml from each culture was subjected to centrifugation. Bacterial pellets of donor cells and recipient cells were washed and re-suspended in 1ml of LB. One hundred μl of each re-suspended culture was mixed and spread on LB agar plate. After 24 h of incubation, bacterial cells were scraped from the agar and suspended in 1 ml phosphate-buffered saline (PBS). Appropriate dilutions of the bacterial growth were spread on MG agar plate (to inhibit the growth of donor strains) supplemented with 50 mg/ml kanamycin (to select the plasmid transformed cells of *P. syringae* MB03) and incubated at 28°C for 48 h.

### Screening of the Transposon Mutant Library for the Loss of Virulence

For the screening of *P. syringae* MB03 attenuated mutants, the liquid killing assay was conducted. Briefly, mutants of *P. syringae* MB03 were grown for 24 h at 28°C. The cells were washed and diluted in S medium supplemented with 5 μg/ml cholesterol. Cell density was adjusted at 0.6_OD600_ and 150 μL of cell suspension was added into the assay. Worms were synchronized and 30–50 L4 synchronized worms were added in the assay as described previously (Powell and Ausubel, 2008). For the inhibition of egg lying, 5-fluorodeoxyuridine (FUdR) was added at a concentration of 50 μg/ml (Meisel et al., 2014). The assay was set in a 96 well-plate incubated at 25°C. Mutants were screened based upon their pathogenicity against worms compared with that of the wild-type *P. syringae* MB03. Genes harboring transposons were identified by HiTAIL PCR, sequencing and the subsequent DNA alignment revealed the insertion position of the transposon.

### Heterologous Expression of Candidate Nematicidal Genes

The genes of potential nematicidal proteins were selected from the results of three different approaches; (1) *de novo* RNA sequencing, (2) comparative genomics, and (3) prediction based upon an online web tool, VirulentPred (Garg and Gupta, 2008). Selected genes were amplified through PCR and were inserted into the pTrcHis series (Invitrogen, Waltham, MA, United States). Newly prepared vectors were cloned into *E. coli* DH5α. The recombinant strains were grown over LB agar containing ampicillin as selection marker. Further verification of cloned genes was performed by PCR and gene sequencing. After final verification, vectors were cloned into *E. coli* strain TOP10 or *E. coli* strain JM109 for protein expression. The His-tag labeled proteins were induced by adding IPTG to the bacterial growth medium, and the proteins were purified with a Ni-NTA affinity column. After dialysis, SDS PAGE was run for the quantitative analysis of proteins and Bradford assay was used for the quantification of proteins (Bradford, 1976). Recombinant cells and purified proteins were used to determine nematicidal activities.

## Results

### Pathogenicity of *Pseudomonas syringae* MB03 Against *Caenorhabditis elegans*

**FIGURE 1:**
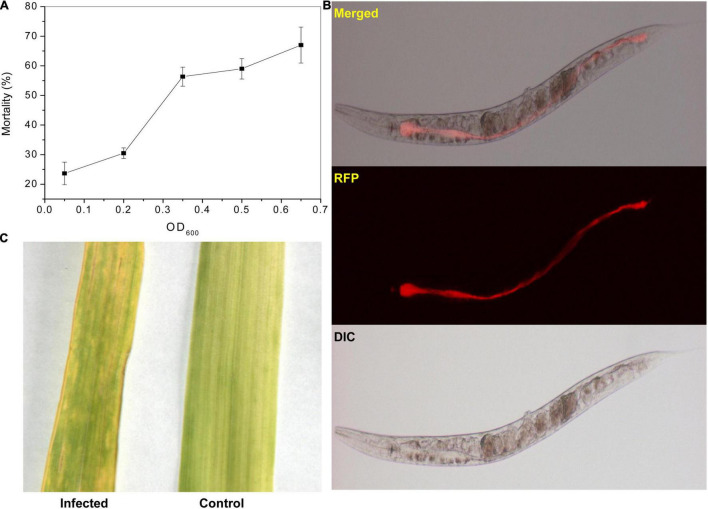
Pathogenicity of *P. syringae* MB03 against *C. elegans* and wheat leaves. The impact of the initial cell density of MB03 on killing *C. elegans* is shown in figure. **(A)** Assay was performed in a 96 well plate. L4 synchronized worms (30–40) were placed in each assay well. The fraction of dead worms was determined after 72 h. The worms were considered dead if no response was shown to touch. Figure shows increase in worm mortality with an increase in initial cell density of *P. syringae* MB03. **(B)** Gut colonization of *C. elegans* by *P. syringae* MB03 is shown. The red fluorescence protein (RFP) expressing vector was cloned into *P. syringae* MB03. The worms were fed on RFP expressing *P. syringae* MB03 and worms were investigated after 48 h to observe distention of anterior and posterior parts of the gut. The red fluorescence is depicting colonization of the host gut by bacterial cells of *P. syringae* MB03 harboring pMCh-23 plasmid. **(C)** Infection of the wheat leaf by *P. syringae* MB03. The bacterial strain was grown overnight, and a cell suspension made in MgSO_4_ was used to infect leaves. Deterioration in the leaf infected with *P. syringae* MB03 is obvious.

The killing assay showed that the *P. syringae* MB03 could kill *C. elegans* in the liquid assay. The percent mortality increased with an increase in cell inoculum (Figure 1A). Interestingly, *P. syringae* MB03 was found to be capable of gut colonization in this liquid-based killing assay. An obvious distention in the anterior and posterior parts of the gut of worms was observed due to bacterial colonization (Figure 1B). Previously, it was reported that gut colonization of *C. elegans* by *P. aeruginosa* was observed during agar-based slow killing (Tan et al., 1999a) whereas liquid killing was not mediated by gut colonization (Kirienko et al., 2013).

### Comparative Genomics for the Identification of Unique Features of *Pseudomonas syringae* MB03

#### Genomic Organization of *Pseudomonas syringae* MB03 Is Highly Similar to the Strains of Phylogroup II

**FIGURE 2:**
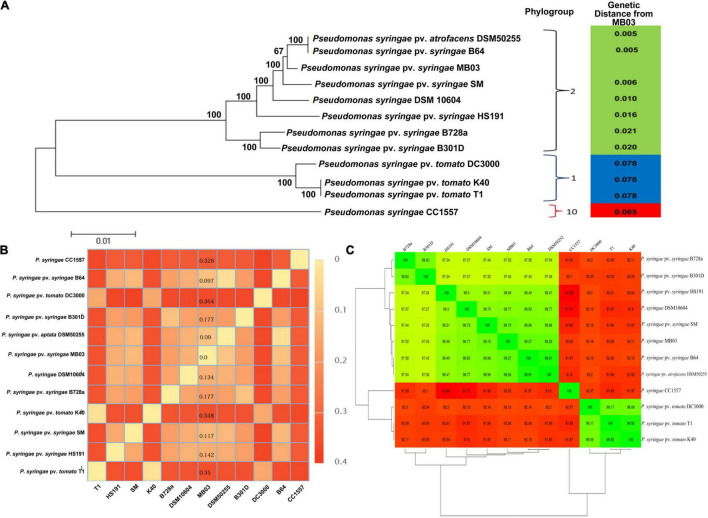
Phylogenetics of *P. syringae* MB03. Phylogenetic analysis of *P. syringae* MB03 was performed based on MLSA using seven housekeeping genes **(A)**. The tree was constructed by the neighbor-joining method by MEGA5 and bootstrap values are shown at internal nodes. Based on seven genes, the genetic distance of MB03 from other strains was determined, shown in the right panel. Strain MB03 was grouped in phylogroup II of *P. syringae* species. Genome-to-genome distance of strain MB03 with other strains is also shown **(B)**. Least distance of *P. syringae* MB03 was observed with *P. syringae* DSM50255, B64 and SM. The mean amino acid identity matrix is shown for selected strains **(C)**. The numerical value within a box shows the percent of the mean amino acid identity of homologs of two strains. Red and green colors are used to show percent similarity.

The *de novo* gene prediction showed that the 5.77 Mb genome of *P. syringae* MB03 contains 5,026 genes with an average gene length of 930 bp. The general features of the *P. syringae* MB03 genome are described in Supplementary Tables 1, 2. Previously, strains of *P. syringae* have been classified into 13 different phylogroups based on multi-locus sequence typing (Berge et al., 2014). All the strains within the same phylogroup showed less than 5% genetic distance (Berge et al., 2014). Results of “Multi Locus Sequence Analysis” (MLSA) showed a common ancestry among strains MB03, DSM50255, and B64. Based on the results of MLSA, MB03 was placed in *P. syringae* phylogroup II (Figure 2A). The strains of phylogroup II, contained very few Type three secretion system genes and were found very active for the ice nucleation damage on plants (Berge et al., 2014). We have previously reported ice nucleation activity of *P. syringae* MB03 (Li et al., 2012). Moreover, genome-to-genome distance (Figure 2B) and an amino acid identity matrix (AAI) were generated (Figure 2C) to further deepen the phylogenetics of MB03. The homologs of MB03 showed the highest AAI to the strains B64 and DSM50255 followed by SM. The results of genomic distance analysis coincided with the results of MLSA and AAI. The strains of phylogroup II such as B64 and SM are known for their potential to infect monocot plants, such as wheat (Dudnik and Dudler, 2013a). Therefore, it was hypothesized that MB03 would be able to infect wheat because it had been predicted as a member of phylogroup II. The pathogenicity of MB03 in wheat was assessed, and the strain could infect the wheat leaf (Figure 1C). Based on these results, MB03 was placed in *P. syringae* phylogroup II (Figure 2A).

The GC skew of MB03 was generated using the CGView server. All the predicted ORFs of MB03 were compared with the reference strains (*P. syringae* B728a, DC3000 and CC1557); the opacity of the line is representing the degree of similarity (Supplementary Figure 1). The genome alignment of MB03, SM and B64 showed noticeable similarity in terms of evolutionary rearrangements (as shown by colored block in Supplementary Figure 2).

#### The Strain-Specific Gene Content of MB03 Is Exceeded by the Hypothetical Proteins

Previously, strains of *P. syringae* were considered harmless to *C. elegans* (Burlinson et al., 2013). Contrary to this, *P. syringae* MB03 could kill *C. elegans*. This raised the possibility of the presence of strain-specific genes in MB03 strain which could involve in host infection. The distinction between the core and dispensable genomes provides better insight into the phenotypes and virulence mechanisms of a strain (Pallen and Wren, 2007). Formerly, the core genome of *P. syringae* was determined (40% identity over 40% length), and the analysis resulted in the determination of 3397 genes as the core genome of *P. syringae* species (Baltrus et al., 2011). When the *P. syringae* MB03 genome was compared (using 40% identity over 40% length as selection criteria) with the previously determined core genome by Baltrus et al. (2011), homologs of 3346 genes (out of 3397 genes) were found in the genome of strain MB03. For the determination of species-common genes and strain-specific genes, *P. syringae* MB03 was compared with DC3000, CC1557, B728a, HS191, B64, and SM (Table 1). The highest number of strain-specific genes was found when *P. syringae* MB03 was compared with DC3000. Moreover, 156 genes of *P. syringae* MB03 showed no homologs in the six tested strains; hence, these genes were specified as unique genes (Supplementary Table 3). Among these unique genes, 135 were predicted as hypothetical proteins. Moreover, four transcriptional regulators (VT47_00425, VT47_00955, VT47_13240, and VT47_22400) were only found in *P. syringae* MB03. Among these regulators, VT47_00420, VT47_00425, and VT47_22400 were found on a predicted genomic island.

**TABLE 1:** Strain-specific genes of *P. syringae* MB03 compared with other reference strains.

		Compared *P. syringae* strains					
		DC3000	CC1557	B782a	SM	B64	HS191
MB03 strain-specific genes^a^		774	744	529	397	386	377
MB03 unique genes^b^	156						

*^a^Genes of P. syringae MB03 not found in the compared strain. ^b^Homologs from P. syringae MB03 genome were not found in any of the reference strains hence, considered unique genes of P. syringae MB03.*

*Further detail of these genes is provided in Supplementary Table 3.*

#### Predicted Genomic Islands of *Pseudomonas syringae* MB03 Have the Potential to Amend the Pool of Virulence Factors

Genomic islands evolve due to horizontal gene transfer and sometimes play a vital role in bacterial lifestyle (O’Brien et al., 2011). Regarding *P. syringae* species, two candidate pathogenicity islands of *P. syringae* B728a have been reported in PAI DB^2^. The *hrp* pathogenicity island of B728a (related to Type III secretion system) contains most of the *hop* effectors of this strain (Psyr_1175 to Psyr_1239). When compared with MB03, integrase, transposase, *hopX1*, *avrB3* and two hypothetical proteins were absent. Alternatively, several additional unique proteins were found in MB03. The rest of the island was similar between the two strains (Supplementary Figure 3). The other candidate pathogenicity island of B728a was not observed in MB03.

When analyzed at Islandviewver3, 24 genomic islands were observed (Supplementary Table 4 and Supplementary Figure 4). To avoid false-positive results, only those islands were analyzed that were present within a single contig. One of these islands contained genes for a Type VI secretion system and hop proteins. This cluster (VT47_11230–VT47_11300) was not present in other *P. syringae* strains. Another important island (VT47_00420−VT47_00465) contained many strain-specific genes. The gene VT47_00420 encodes a LasR transcriptional regulator that is absent in the genome of HS191, B728a, DC3000, B64, SM, and CC1557. According to the KEGG pathway and previous literature reports, the homologs of LasR transcriptional regulators have a well-defined role in bacterial pathogenicity (Feinbaum et al., 2012). LasR controls the expression of hundreds of genes including quorum sensing, secreted virulence factors, and secondary metabolites (Gilbert et al., 2009).

### Transcriptomics for Determining Bacterial Response During Host–Pathogen Interaction

#### The Transcriptional Response of the Pathogen Varied Depending Upon Its Growth Phase

**FIGURE 3:**
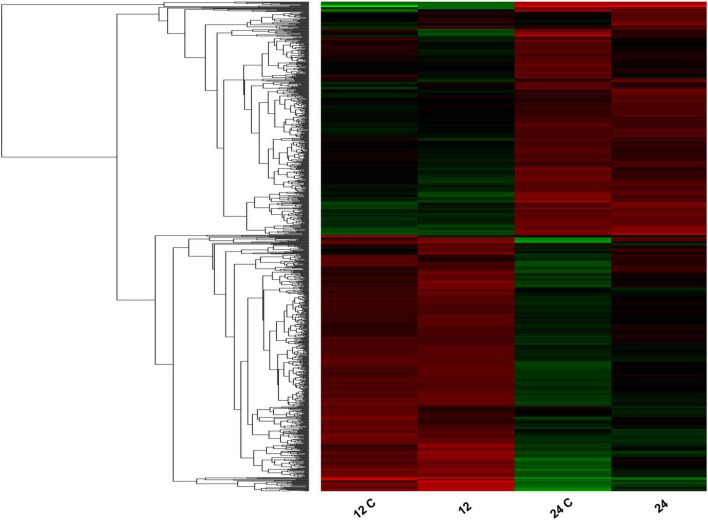
Heatmap showing differential expression of bacterial genes in response to *C. elegans*. Total RNA was extracted from the bacterial cells with/without the host from two different phases of bacterial growth (exponential phase represented as 12 and 12 C whereas stationary phase represented as 24 and 24 C). Control samples of 12 h growth and 24 h growth are represented as 12 and 24, respectively whereas bacterial cells cultured with *C. elegans* are shown as 12 C and 24 C. Red bars show up-regulation and green bars show downregulation of genes. The intensity of the color is directly proportional to the magnitude of gene expression. Results of transcriptomics were cross verified by performing qRT-PCR on selected genes.

*Pseudomonas syringae* MB03 cells were exposed to the model host *C. elegans* during the exponential phase, as well as the stationary phase of bacterial growth, to analyze the transcriptome of MB03 cells. Various genes were differentially expressed depending on the growth phase of the pathogen (Figure 3). In total, 76 and 138 genes showed log_2_ ≥ 2-fold differential expression when the host–pathogen interaction took place during the exponential and stationary phase, respectively (Supplementary Table 5). Among these genes, only 11 genes overlapped and showed similar patterns of expression regardless of the growth phase. Up-regulated genes of interest included *atoE* (V47_15130) for short-chain fatty acid degradation; *hisJ* (VT47_18480), which is a part of an ABC transporter for histidine transport; and *gltI* (VT47_18645), which might be correlated to a shift in nutrient availability. Additionally, *pntA* (VT47_24115) and *scoB* (VT47_15125) were up-regulated, whereas *scoP* (V47_01435), *queD* (VT47_04670), *rpsT* (VT47_03545), and two hypothetical proteins (VT47_13300, VT47_14685) were down-regulated during host–pathogen interaction regardless of the bacterial growth phase (Supplementary Table 5).

Genes for the utilization of D-galactonate (VT47_09385, VT47_09390, and VT47_09395) were only up-regulated in the exponential phase cells. Another important difference in the gene expression pattern was observed in the case of ABC transporters primarily related to amino acids. In bacterial two-component systems, *phoP*/*phoQ* genes were up-regulated when interaction was studied with exponentially growing bacterial cells. This system has been documented to trigger various bacterial virulence factor during environmental stress and host pathogen interaction (Alegado and Tan, 2008; Gellatly et al., 2012). Similarly, genes for tricarboxylate transport (*tctA* VT47_18945, *tctB* VT47_18940, *tctC* VT47_18935) and amino acid uptake and metabolism (*aatP* VT47_18660, *aatM* VT47_18655, *aatQ* VT47_18650, *aatJ* VT47_18645) were up-regulated during the exponential phase interaction. It has been reported that most of hosts enforce amino acid starvation to the invading bacteria. In response, bacterial pathogens manipulate the host metabolism to overcome nutrient depletion (Zhang and Rubin, 2013). Most genes related to flagellar assembly were up-regulated when bacterial cells in the stationary phase were used in host-pathogen interaction (Supplementary Table 5). Previously, flagellum has been reported for its role in the adhesion and penetration of pathogen into host tissue (Haiko and Westerlund-Wikstrom, 2013; Coloma-Rivero et al., 2020). Moreover, in our previous study, genes related to flagellum were found up-regulated during host-pathogen interaction (Ali et al., 2016).

Among the genes present on the predicted genomic islands, five genes showed highly differentiated expression when MB03 was exposed to *C. elegans* (Supplementary Table 6). All these genes were part of the auxiliary genome of *P. syringae*. Among these genes, VT47_06210 was responsible for the conversion of nitroalkane into nitrite, whereas VT47_06205 was involved in nitric oxide detoxification. Previously, nitric oxide detoxification genes have been reported in certain strains of *P. syringae* (Marcelletti et al., 2011). Analysis of the genome organization revealed the presence of one transcriptional regulator (VT47_06200) just upstream of these genes. This transcriptional regulator was annotated as *norR*, which has also been reported for nitric oxide detoxification.

#### Up Regulation of Bacterial Two-Component Systems Might Help the Bacterial Strain in Sensing the Host Environment

According to COG, 282 genes of MB03 were grouped under the signal transduction category (Supplementary Table 2). Among two-component systems (TCS), *phoQ* of *phoQ*/*phoP* was up-regulated during host-pathogen interaction regardless of the growth phase of the bacterial pathogen. Previously, up regulation of this system has been reported in *P. aeruginosa* cells after interaction with human bronchial epithelial cells, and mutation in the *phoQ* gene resulted in reduced virulence (Gellatly et al., 2012). This system has also been known to confer antimicrobial resistance to the bacterial cells. Previously, it was reported that the *phoP* mutant of *Salmonella enterica* was unable to colonize the gut of *C. elegans* (Aballay et al., 2000).

Other TCS related to bacterial pathogenicity include *fleS*/*fleR*, which regulates flagellum biosynthesis along with *rpoN* sigma factor (Dasgupta et al., 2003). Previously, the synthesis of flagellum and transcription of biosynthetic genes have been divided into four phases. The FleS/FleR system was reported to control expression of genes required for transcription of class III genes of the flagellum (Dasgupta et al., 2003). Down regulation of this TCS during the exponential phase interaction and up-regulation during the stationary phase interaction highlighted its role in the late phase of MB03 infection of *C. elegans* (Supplementary Table 5). Interestingly, the same transcriptional pattern was observed for *rpoN.* The genes related to flagellum synthesis and functioning were also up-regulated during the stationary phase interaction.

The KdpD/KdpE TCS has been reported not only to regulate intracellular potassium concentration but also to play an important role in bacterial virulence (Freeman et al., 2013). Bacterial species such as enterohemorrhagic *Escherichia coli*, *Francisella tularensis*, and *Salmonella typhimurium* having mutated *kdpD*/*kdpE* genes, showed significantly attenuated virulence when studied in different host cells (Alegado et al., 2011; Njoroge et al., 2012). Accordingly, *kdpD* mutants of *P. aeruginosa* and *S. typhimurium* species were attenuated in killing *C. elegans* (Alegado et al., 2011; Feinbaum et al., 2012). A fully functional *kdpD* gene was required for colonization of the host gut by bacterial cells. In this study, no significant change was observed in the expression of the *kdpD*/*kdpE* two component system. However, the *kdpB* gene was significantly up-regulated during the stationary phase interaction. Previously, it was reported that a *kdpB* mutant of *P. aeruginosa* PAO1 was attenuated in its virulence against *C. elegans* (Dubern et al., 2015). Another important two-component system is *gacA*/*gacS*, which is well-studied for its role in bacterial virulence. However, in our transcriptomics data, no notable change was observed in the expression of this system.

#### Genes of Type III and VI Secretion Systems Were Differentially Expressed During Host–Pathogen Interaction

In the case of *P. syringae* pathovars, the Type III secretion system and its effectors (TTE) are among the major factors in its plant host determination (Lindeberg et al., 2006). To date, more than 50 TTEs have been identified in *P. syringae* strains (Baltrus et al., 2011). Their sequences were obtained to identify homologs in strain MB03 by applying BLASTp, and a total of 10 effectors were identified (Supplementary Table 7). Five effectors (AvrE, HopAA, HopAH, HopI, and HopM), which are found in almost all of the strains (Baltrus et al., 2011), were also present in MB03. Previously, it was reported that members of *P. syringae* genomogroup II contain fewer effectors compared to other groups. *P. syringae* B64, which was isolated from the wheat plant, contained 10 effector proteins (Dudnik and Dudler, 2013b), and out of those, six are common between B64 and MB03 (HopZ3 and five core effectors including AvrE1, HopAA1 HopI1 HopM1, and HopAH1).

Although most of the genes related to the Type III secretion system and effectors showed no change in their transcription levels, *hrcN* (VT47_05965) and *hrcQa* (VT47_05955) were up-regulated when stationary phase MB03 cells were exposed to the worms. Both of these proteins are among the core proteins of Type III secretion system and share significant similarities with flagellar proteins (Van Gijsegem et al., 1995). Other than the well-known motility function of the flagellum, secretion of proteins especially virulence factors has also been well-documented (Young et al., 1999).

#### Type VI Secretion System

Among bacterial secretion systems, the Type VI secretion system plays an important role in the host-microbe interaction and virulence (Mougous et al., 2006; Jiang et al., 2014). However, its role in *P. syringae* is still not well-characterized. Recently, an effort was made to analyze the distribution of the Type VI secretion system in *P. syringae* species by *in silico* characterization (Sarris et al., 2010). Although we observed two Hcp secretion islands (HSI) clusters in MB03 (VT47_23900–VT47_23835 and VT47_11235–VT47_11300), these were not identical to the clusters found in DC3000 in terms of gene number and function. The Type VI secretion system of B728a is comprised of the HSI-I cluster, Ppka locus, and certain *hcp*/*vgrG* genes. The Ppka and Pppa were observed in many strains of *P. syringae* such as B728a, DC3000 and T1 (Sarris et al., 2010). Compared with B728a, the Ppka locus along with homologs for genes Psyr_0101 and Psyr_1935 were absent in MB03. The *ppkA* and *pppA* are regulatory genes, and both work antagonistically. The homolog of PpkA locus was not found in MB03. However, this is not unusual as many other bacterial strains with missing PpkA loci have been reported (Sarris et al., 2010). Except for one gene, *impM* (VT47_23885), the entire cluster was up-regulated during stationary phase host-pathogen interaction. Two Rhs element Vgr proteins (Valine-glycine repeat protein, an essential component of secretion machinery) (VT47_23935 and VT47_23990) were also up-regulated.

Moreover, MB03 contained another contiguous cluster (Supplementary Figure 5) encoding for a Type VI secretion system (VT47_11230–VT47_11300). This cluster of MB03 was absent in strain B728a; however, homologs of genes of this cluster were found in B64, and SM. The ClpB/V, DotU, and IcmF are among the core components of T6SS, and the VT47_11270 loci of MB03 encodes a ClpB protein, which can provide energy to the secretion system. Interestingly, this cluster was accompanied by two transcriptional regulators: one σ^54^ dependent (VT47_11275) within the cluster and another LysR family protein (VT47_11320) downstream of the cluster. Previously, a σ^54^ dependent transcriptional regulator was reported to play an important role in the regulation of the T6SS clusters (Bernard et al., 2011). Among the genes of HSI-II, *impA*, which encodes a secretion protein, was up-regulated during the stationary phase interaction. There is no direct evidence to explain the role of these Type VI effector proteins; however, it appears that these proteins might have important roles in the animal pathogenicity of *P. syringae* MB03.

### Genes Related to Locomotion and Adhesion Were Up Regulated During Host–Pathogen Interaction

The flagellum has a well-defined role in host colonization, locomotion, protein secretion, and chemotaxis, and all these functions help in pathogenesis (Young et al., 1999; Dasgupta et al., 2003). Mutations in alginate biosynthesis genes of *P. syringae* pathovars lead to a significant decrease in plant pathogenesis (Dorati et al., 2018). In the current study, *alg44*, *algE*, and *algD* were upregulated during the exponential phase interaction, whereas *algD*, *algE*, *algF*, *algL*, and *alg8* were upregulated during the stationary phase interaction. Interestingly, the genes related to the flagellum were down-regulated during the exponential phase interaction and up-regulated during the stationary phase interaction (Supplementary Table 5). In *P. aeruginosa*, transcription of flagellum-related genes is controlled by *fleQ*, *vfr*, and other sigma factors (Dasgupta et al., 2003). Gene *fleQ*, which regulates transcription of class 1 flagellum genes, was also down-regulated during the exponential phase interaction. On the other hand, almost all the genes for the flagellum were up-regulated when the stationary phase MB03 was cultured with *C. elegans*. Animal pathogens have been well-reported for protein secretions through the flagellum (Fretin et al., 2005; Hautefort et al., 2008). Late expression of the flagella-related genes in MB03 infection is in accordance with a previous report where flagellum-related genes were expressed in the late phase of infection (Hautefort et al., 2008). Up regulation of flagellum-related genes during host–pathogen interaction might be related to protein secretion and motility. Possibly, this up-regulation occurred after infection by the pathogen as it was previously observed in the case of the *Salmonella* strain (Hautefort et al., 2008). Additionally, the involvement of flagellar genes in the killing of *C. elegans* has also been reported in *Burkholderia pseudomallei* (O’Quinn et al., 2001).

### Homologs of Nematicidal Genes of *Pseudomonas aeruginosa* in MB03 and Their Transcriptional Response

Virulence factors of different pathogens, such as *P. aeruginosa*, have been previously characterized using *C. elegans* as a model organism; a total of 170 nematicidal genes in *P. aeruginosa* PA14 (Feinbaum et al., 2012) and 68 in *P. aeruginosa* PAO1 have been identified (Dubern et al., 2015). To further evaluate the nematicidal potential of MB03, its proteins were compared with the reported virulence factors of *P. aeruginosa* PA14 and PAO1. For this purpose, *in silico* subtraction was used, and conserved proteins were retrieved (*H*-value ≥ 0.64). This revealed 87 and 30 homologs of PA14 and PAO1, respectively, in MB03 (Supplementary Table 8). Among these, 10 homologs showed significant variation in their transcriptional profiles (Table 2) upon host-pathogen interaction. Six homolog genes were up regulated during the stationary phase interaction: *lysR* transcriptional regulator (VT47_13020), isovaleryl-CoA dehydrogenase (VT47_11890), putative acyl-CoA carboxylase alpha chain (VT47_11875), acyl-CoA carboxyltransferase beta chain (VT47_11885), *prpC* (VT47_09990), and PrpB (VT47_09985). On the other hand, *crfX* (VT47_10030), (VT47_19750) and hypothetical protein (VT47_10035) were down regulated (Table 2). Regarding nematicidal genes of PAO1, homologs of 30 genes were found in MB03, as well as in the core genome of *P. syringae* (*H* value ≥ 0.64). Among these 30 homologs, one named *kdpB* was markedly up regulated.

**TABLE 2:** Homologs of *P. aeruginosa* PA14 and PAO1 nematicidal genes in *P. syringae* MB03 showing differential transcriptional response.

Gene	Locus tag	Homolog in the core genome of *P. syringae*	Transcriptional response at different interaction stages^a^		Function
			12 h	24 h	
	VT47_13020	Yes	Unchanged	Up	LysR family transcriptional regulator
	|VT47_11890	Yes	Unchanged	Up	Isovaleryl-CoA dehydrogenase
	VT47_11875	Yes	Unchanged	Up	3-Methylcrotonyl-CoA carboxylase alpha subunit
	VT47_11885	Yes	Unchanged	Up	Propionyl-CoA carboxylase
*prpC*	VT47_09990	Yes	Unchanged	Up	2-Methylcitrate synthase
*prpB*	VT47_09985	Yes	Unchanged	Up	2-Methylisocitrate lyase
*cmpX*	VT47_10035	Yes	Unchanged	Down	Hypothetical protein
*ibaG*	VT47_19750	Yes	Unchanged	Down	BolA-like protein
	VT47_10030	Yes	Unchanged	Down	CrfX protein
*kdpB*	VT47_09695	Yes	Unchanged	Up	Potassium-transporting ATPase subunit B

*^a^Differential expression was investigated during two growth phases of the bacterial pathogen, the exponential phase (12 h) and stationary phase (24 h).*

#### Up Regulation of ABC Transporters Provide Insight Into Nutritional Availability

Comparative genomics revealed the presence of six ABC transporter genes of MB03 (VT47_07640, VT47_08280, VT47_08285, VT47_12125, VT47_13510, VT47_20470) homologs, which were previously demonstrated to be essential for full bacterial virulence of *P. aeruginosa* against *C. elegans* (Feinbaum et al., 2012; Dubern et al., 2015). The results of the transcriptomics showed up-regulation of different genes related to ABC transporters (Table 3). Glutamate/aspartate transporters encoded by *gltI, gltJ, gltK*, and *gltL*, were highly up-regulated when MB03 cells were exposed to *C. elegans*. This system binds and transports glutamate and aspartate amino acids to the bacterial cell. It has been proposed that the release of these amino acids indicated disruption of host cells and that bacterial cells used these as sources of carbon and nitrogen (Hassel et al., 2014).

**TABLE 3:** Transcriptional profile of ABC transporters during the exponential and the stationary phase interactions.

Transporter	Genes	Expression		Description
		12 h	24 h	
Phosphate and amino acid ABC transporter				
Phosphate	*pstSCAB*	Up	Partially Up regulated^b^	Upregulation of the phosphate transporter has been previously reported in *Salmonella* infection of epithelial cells (Hautefort et al., 2008)
Lysine/Arginine/Ornithine	*argT, hisMQP*	Up	Highly Up	The presence of amino acids in the extra cellular environment was co-related to host tissue damage (Hassel et al., 2014).
Glutamate/Aspartate	*gltIKJL*	Up		
General L-amino acid	*aapJQMP*	Up	Up	
Branched-chain amino acid	*livKHMGF*	Up	Up	
Histidine	*hisMP* ^a^		Partially Up-regulated ^b^	
Mineral and organic ion ABC transporter				
Alkanesulfonate	*ssuACB*	Down		
Glycine betaine/Proline	*proXWV*	Up		
Oligosaccharide and polyol ABC transporter				
	*smoEG*		Partially up regulated^b^	
Monosaccharide transporter				
Ribose/Autoinducer 2/D-Xylose	*rbsBCAD*	Up	Partially up regulated^b^	Highly up-regulated at 12 h, whereas only *rbsB* gene was up-regulated at 24 h
L-Arabinose	*araFHG*	Up		
D-Xylose	*xylFHG*	Up	Partially up regulated^b^	Only *xylF* gene showed noticeable induction at 24 h
ABC-2 transporter				
Lipopolysaccharide	*rfbAB*		Up	

*^a^All the genes of the ABC transporter were not differentially expressed. Genes that showed variation in their expression levels were presented. ^b^Some of the genes of the ABC transporter showed variation in transcriptional profile.*

Also, glycine betaine/proline transporter was up-regulated during the exponential phase interaction (Table 3). This has also been reported for osmoprotection in *P. aeruginosa* (Wargo, 2013). Moreover, glycine betaine uptake by *proXVWZ* ABC transporter was found to be vital for the growth and survival of *Mycobacterium tuberculosis* in human macrophages (Price et al., 2008).

#### Various Transcriptional Regulators Showed Significant Differential Expression During Host–Pathogen Interaction

Eight transcriptional regulators showed significant change in their expression profiles when *P. syringae* MB03 interacted with *C. elegans* (Table 4). Out of these transcriptional regulators, the homolog of one gene (LysR family, VT47_13020) was reported as essential for full virulence of *P. aeruginosa* PA14 against *C. elegans* (Feinbaum et al., 2012). The LysR family transcriptional regulators are well-documented for their impact on bacterial pathogenicity by regulating numerous virulence factors, helping in bacterial adhesion and adaptation to a hostile environment (Xiao et al., 2006). Two other regulators, *araC* family, and *betI*, were differentially expressed when *P. syringae* MB03 interacted with *C. elegans*. Among these regulators, the AraC family transcriptional regulator was up-regulated during the exponential and stationary phase interactions. This regulator is located upstream of glycine betaine/proline ABC transporter genes (*proXVW*) in the genome of *P. syringae* MB03. Genes for betaine transport were also up-regulated during the exponential phase interaction. Transcriptional factors related to the AraC family have been reported for carbon metabolism, bacterial pathogenicity, and stress response (Gallegos et al., 1997).

**TABLE 4:** Expression profile of selected transcriptional regulators and their function.

Locus tag	Expression^a^		Product	Description	PS core*^b^*
	12 h	24 h			
VT47_06065		Down	DNA-binding transcriptional regulator, IscR family	The regulator is present upstream of iron-sulfur cluster operon (*isc*SUA). It represses the expression of the iron-sulfur cluster.	Yes
VT47_09405		Up	IclR family transcriptional regulator		Yes
VT47_10490	Up		GntR family transcriptional regulator		No
VT47_13020		Up	DNA-binding transcriptional regulator, LysR family		Yes
VT47_16235		Up	Flagellar biosynthesis regulator FlhF	It regulates the synthesis of flagellar proteins, and it is essential for the placement and assembly of polar flagella.	Yes
VT47_22635	Up	Up	AraC family transcriptional regulator	The regulator is present upstream to glycine/betaine ABC transporter genes (*proXVW*).	Yes
VT47_22755	Down		BetI family transcriptional regulator	This is a transcriptional repressor of betaine regulon. Betaine biosynthesis genes *betA*/*betB* are found adjacent to this regulator.	Yes
VT47_24210		Up	Chemotaxis protein CheY	This CheY family protein is flanked by PhoR/PhoB two-component system and phosphate ABC transporter.	Yes

*^a^Differential expression was investigated during two growth phases of the bacterial pathogen, the exponential phase (12 h) and stationary phase (24 h). ^b^Comparison was done with the core genome determined by Baltrus et al. (2011).*

### Screening of a Mutant Library Revealed Nematicidal Genes of *Pseudomonas syringae* MB03

The construction of a transposon insertion mutant library resulted in 1265 mutants of *P. syringae* MB03. The killing assay was performed to screen for mutants with attenuated virulence. For comparison, *P. syringae* wildtype strain MB03 was used, and all the worms died after 7–8 days when exposed to the wild-type strain. Primary screening resulted in the identification of 12 mutants with attenuated virulence. The second round of screening was performed which resulted in the identification of seven mutants (Table 5). The worms survived for at least 10 days when exposed to these attenuated mutants. These seven mutant genes were assessed for their homologs in the previously reported virulence factors of Gram-negative bacteria, especially virulence factors of *P. aeruginosa* and *P. syringae* (Feinbaum et al., 2012; Dubern et al., 2015). For this purpose, VFDB was also searched; however, homologs of these seven genes have not been previously reported for their virulence against *C. elegans*.

**TABLE 5:** Nematicidal genes identified by transposon insertion mutant library screening.

Locus tags	Size (bp)	Predicted function	Transcriptional response	Genome distribution
VT47_19645	1815	Lipoprotein		Auxiliary Genome
VT47_06935	747	Hypothetical protein		Core genome
VT47_06900	1284	Outer membrane porin (*oprD*)	Up regulation at stationary phase	Core genome
VT47_00795	1398	Glutamate: protein symporter (*gltP*)		Core genome
VT47_19690	1095	Putative ATPase (*zapE*)		Core genome
VT47_09995	2589	Aconitate hydratase (*acnA*)	Up regulation at stationary phase	Core genome
VT47_23445	171	Hypothetical protein		Auxiliary genome

Among these mutants, VT47_19690 was identical to *zapE* of *E. coli* strain K-12 and a hypothetical protein (PA4438) of *P. aeruginosa* PA14. Recently, the ZapE protein has been reported for its role in cell division, and the protein is required for bacterial infection (Marteyn et al., 2014). The importance of cell division has also been elucidated in the *P. aeruginosa* – *C. elegans* model, where a mutation in the *minD* gene resulted in attenuated virulence (Feinbaum et al., 2012). Another mutant strain Δ*acnA* (VT47_09995) showed attenuated virulence. The protein can interconvert citrate and isocitrate thereby facilitating a shift in different metabolic pathways, including the citrate cycle and glyoxylate cycle. Similarly, the mutation in the *oprD* (V47_06900) gene resulted in attenuated pathogenicity against *C. elegans.* The gene *oprD* encodes porin which forms a channel in the bacterial cell membrane. Different functions have been associated with membrane porins, including resistance against antimicrobials, nutrient uptake, and adhesion (Tamber et al., 2006; Muller et al., 2011). In addition, the porin of *P. aeruginosa* may also possess protease activity (Yoshihara et al., 1996).

### *In silico* Characterization of Selected Hypothetical Proteins and Transcriptional Regulators

The hypothetical proteins and transcriptional regulators, which showed differential expression during transcriptomics analysis were selected for *in silico* characterization. Molecular weight, isoelectric point, aliphatic index, and stability of the hypothetical proteins were predicted by Protparam software. Among 25 hypothetical proteins, 12 were found to be unstable (Supplementary Table 9). Signal peptides were predicted at the N-terminus of 7 hypothetical proteins, indicating their localization in periplasmic space, outer or inner membrane. This notion was further validated by CELLO and TMHMM results. Seven hypothetical proteins were predicted in the periplasmic space while four were associated with the outer and inner membranes. The proteins on the outer membrane could be potentially involved in the host-pathogen interaction, and therefore, 3D structures of these were predicted using I-TASSER. Interestingly, one hypothetical protein (Accession No: KZL40945) was predicted to be involved in host–pathogen interaction by the VICMpred server. This was also supported by our experimental data from the transposon insertion mutant library screening. Moreover, this protein is predicted as a transcriptional regulator based on homology modeling by the HPIDB server. Similarly, uncharacterized transcriptional regulators which showed highly differential expression were selected to predict target genes (Supplementary Table 10).

### Heterologous Expression of Predicted Virulence Factors

**FIGURE 4:**
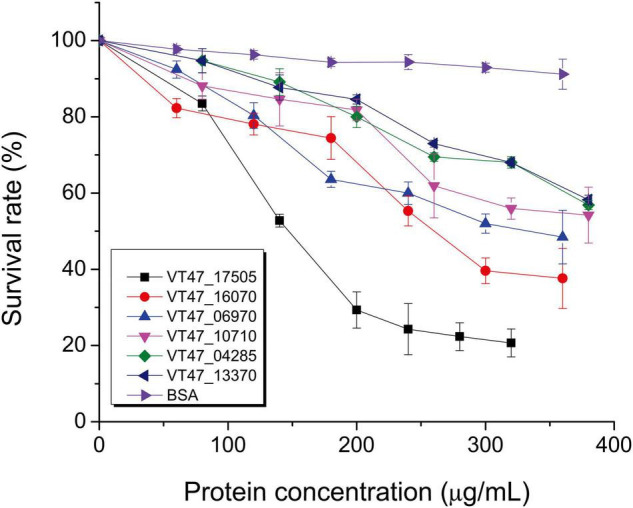
Nematicidal activity of purified bacterial proteins. Potential nematicidal proteins of *P. syringae* MB03 were expressed in *E. coli* TOP10 or JM109 and purified by affinity column chromatography for the liquid killing assay. The killing of worms was determined after 3 days. Worms that did not respond to the touch were considered dead. Bovine serum albumin (BSA) was used as a control. The experiment was performed in triplicate and mean values are represented. On *X*-axis, concentrations of purified proteins are shown. On *Y*-axis, survival of *C. elegans* is shown. NCBI accession numbers of the genes are shown in text box (VT47_17505 esterase, VT47_16070 undefined protein, VT47_06970 membrane protein, VT47_10710 chitinase, VT47_04285 MCP, VT47_13370 MCP). The details and selection criteria of these proteins are provided in Table 6.

Proteases were expressed in *E. coli*, and their impact on virulence acquisition by living cells was investigated. For this purpose, the fraction of killed worms was determined during the interaction of recombinant *E. coli* strains and *C. elegans*. The screening resulted in the identification of potential proteases and esterases (Table 6 and Figure 4). Among these enzymes, VT47_14740 showed 54% identity with metalloproteinase serralysin (AFX62372) of *S. marcescens* (Paiva et al., 2013). VT47_14210 also showed 34% identity with metalloproteinase serralysin (AFX62372). The protein VT47_17780 had 33% identity with a serine protease precursor (JX667979) of *S. marcescens* (Paiva et al., 2013). However, the results showed high up-regulation of a metallo-protease serralysin (VT47_14740) during the stationary phase interaction (Supplementary Table 5). Recently, a serralysin-like protein has been reported to play an important role in the pathogenicity of *Serratia marcescens* against insects (Pineda-Castellanos et al., 2015).

**TABLE 6:** Heterologous expression of proteins and nematicidal activity of recombinant *E. coli* cells.

Protein	Locus tag	Function	Methodology	Killing^a^
Proteases				
	VT47_24200	Hemolysins and related proteins containing CBS domains R	Genomics	±
Metalloprotease	VT47_14210	ZnMc superfamily	Genomics	+
	VT47_14120	Protease M4 superfamily	VirlentPred	+ +
	VT47_04845	Peptidase superfamily M48	VirlentPred	+
	VT47_20845	Putative metalloprotease Psyr	VirlentPred	+
	VT47_20605	Zn_peptidase superfamily	VirlentPred	+
Alkaline protease	VT47_14725	Alkaline protease secretion protein AprE	VirlentPred	+
	VT47_14720	Alkaline protease secretion protein AprF	VirlentPred	+ +
Serine protease	VT47_18880	Trypsin-like serine proteases, typically periplasmic, contain C-terminal PDZ domain	Genomics	±
	VT47_05790	Easterase_lipase super family	VirlentPred	+
	VT47_21025	Easterase_lipase super family	VirlentPred	+ +
Esterase	VT47_17505	NLPC_P60 family	VirlentPred	+ +
	VT47_09750	Methyl-accepting chemotaxis protein	VirlentPred	+ +
Chitinase	VT47_10710	Methyl-accepting chemotaxis protein	VirlentPred	+ +
MCP	VT47_04285	Methyl-accepting chemotaxis protein	VirlentPred	+ +
	VT47_03775	Methyl-accepting chemotaxis protein	VirlentPred	+ +
	VT47_13370	Methyl-accepting chemotaxis protein	VirlentPred	+ +
	VT47_05595	Chemotaxis family	VirlentPred	+
	VT47_06255	RtxA structural toxin protein	VirlentPred	+ +
CP	VT47_04590	RTX toxins	VirlentPred	+
RTX	VT47_14740	RTX toxins and related Ca2+-binding proteins	Genomics	+ +
	VT47_21930	Iron-sulfur cluster assembly protein	Transcriptomics	+ +
	VT47_01265	Conserved blocks	Transcriptomics	+
Bacterioferritin	VT47_17830	P-loop-NTPase superfamily	VirlentPred	+ +
IscA	VT47_06970	TolA colicin import membrane protein	VirlentPred	+ +
Undefined protein	VT47_16070	Pertactin-like passenger domains	VirlentPred	+ +
Antimicrobial peptide	VT47_19645	PBP1-YraM-Lppc-lipoprotein like	VirlentPred	+
Adhesin				
Lipoprotein				

*^a^Assay was performed in triplicate and mean values are shown. The results of killing were grouped as +++ ≥ 80%, ++= 50–80%, += 20–50% and ±= 10–20%.*

## Discussion

*Pseudomonas syringae* has been traditionally recognized as a plant pathogen; however, notable killing activity against the animal model *C. elegans* has been recently demonstrated for a *P. syringae* wild-type strain MB03 (Ali et al., 2016; Bashir et al., 2020, 2021). Various killing mechanisms of *P. syringae* MB03 such as gut colonization of host (Ali et al., 2016), production of secondary metabolites (Bashir et al., 2020), and nematicidal proteins (Manan et al., 2018) have been discovered. For instance, strain MB03 was capable of gut colonization under nutrient-rich conditions (Ali et al., 2016) and secreted pyoverdine under an iron-deficient environment which mediated host killing (Bashir et al., 2020). In the current study, comparative genomics, transcriptomics, and transposon insertion mutant library analyses were applied for genome-wide identification of common and unique virulence factors required for the nematicidal activity of *P. syringae* MB03 against *C. elegans*. The results indicated that contrary to the *P. aeruginosa* liquid-based killing mechanism, *P. syringae* MB03 colonized the gut of the worm, and lethality followed an infection-like course. When analyzed at the genetic level, we identified some unique potential nematicidal virulence factors, as well as factors for locomotion, nutrient acquisition, adhesion to host, and protein secretion that could play important roles in nematode killing.

Classification of potential virulence factors into strain-specific, auxiliary and core genome showed that most of the virulence factors were from the core genome *P. syringae*. In the case of genes showing high differential expression (log2 ≥ 2) during host–pathogen interaction, approximately 70% of the genes were part of the core genome. The fraction of the core genome also outnumbered the auxiliary genome during the determination of nematicidal homologs of *P. aeruginosa* and from mutant library screening. It should be noted that approximately 66% of genes of MB03 contribute to the core genome, and the remaining 34% of the genome is comprised of auxiliary and strain-specific genes. Hence, it can be concluded that the distribution of virulence factors was not biased toward the core genome or auxiliary genome. The core genome of *P. syringae*, which was employed in comparative genomics, was determined by considering three diverse phylogroups of the *P. syringae* species (Baltrus et al., 2011). Moreover, most strain-specific genes were hypothesized to reside on genomic islands, and indeed, 66 strain-specific genes (42%) were found on the predicted genomic island (Supplementary Table 4).

Previously, near-complete transposon insertion mutant libraries were constructed to identify virulence factors of *P. aeruginosa* strains PAO1 and PA14 (Feinbaum et al., 2012; Dubern et al., 2015). These genome-wide studies revealed various genes related to nematicidal activity including enzymes, secondary metabolites, and two-component systems, etc. Comparative genomics was applied to identify the homologs of those genes in *P. syringae* MB03. The analysis resulted in the identification of 115 candidate genes that may assist pathogens during host-pathogen interaction.

Transcriptomics provided information about a variety of candidate nematicidal genes of *P. syringae* MB03, and the results showed a notable influence of the growth phase on the expression of bacterial genes during host–pathogen interaction. Moreover, the expression of some of the genes was in accordance with previous reports. For example, certain MB03 homolog genes of nematicidal proteins of *P. aeruginosa* showed differential expression (Table 2; Feinbaum et al., 2012; Dubern et al., 2015). It is worth noting that these *P. aeruginosa* homologs showed differential expression during the stationary phase interaction. Similarly, the expression of two-component signal transduction systems can be justified based on previous reports showing their role in bacterial pathogenicity. Up regulation of the *phoQ*/*phoP* and *fleS*/*fleR* two-component systems during the stationary phase, interaction might be correlated to bacterial infection and colonization of *C. elegans* (Alegado and Tan, 2008; Gellatly et al., 2012; Ali et al., 2016).

Different mechanisms have been proposed by which bacterial species kill *C. elegans*. The most common pathogenicity mechanisms include toxin secretion, gut colonization, and persistent infection (Cezairliyan et al., 2013). In the case of toxin-mediated killing, various metabolites such as pyoverdine, phenazine, pyochelin, pyrrolnitrin have been identified in the *Pseudomonas* – *C. elegans* infection model (Cezairliyan et al., 2013; Kirienko et al., 2013). Comparative genomics revealed that MB03 only possessed genes for pyoverdine. In addition, agar-based pathogenicity of *P. aeruginosa* was found to be dependent upon quorum sensing (Feinbaum et al., 2012), a mechanism by which population-dependent genes are regulated. The strain B728a harbors two mechanisms for quorum sensing, *ahlI-ahlR* and *hdtS* (Feil et al., 2005). Similar quorum sensing-related genes were observed in B64, SM, and HS191 (Dudnik and Dudler, 2013a,b; Ravindran et al., 2015). However, only the *hdtS* (VT47_24855) homolog was found in MB03, and no significant change in the expression of *hdtS* was observed. Hence, *hdtS* did not appear to play a significant role in the killing of *C. elegans*.

Siderophores are low-molecular-weight iron chelators that are secreted outside the cell. In DC3000, three different iron chelators, including pyoverdine, pyochelin, and yersiniabactin, have been reported. However, only pyoverdine and achromobactin were found in MB03. Recently, it has been reported that pyoverdine alone was sufficient to kill *C. elegans* (Kirienko et al., 2015). Interestingly, in a liquid killing assay, mutations in pyochelin biosynthesis genes showed no effects on the virulence of *P. aeruginosa* against *C. elegans*, whereas mutations in *pvdA, pvdD, pvdE, pvdF*, and *pvdP* resulted in decreased killing efficacy (Kirienko et al., 2013). A recent study conducted on *P. syringae* MB03 demonstrated the role of pyoverdine in the killing of *C. elegans* (Bashir et al., 2020).

Other than secreted metabolites, bacterial enzymes, and proteins capable of host degradation also play a vital role in bacterial invasion and infection (Matsumoto, 2004; Yoon et al., 2018). Similarly, signaling molecules such as cyclic-di-GMP secreted by *V. cholerae* attracted *C. elegans* toward pathogen (Angeloni et al., 2020). In the case of enzymes, alkaline proteases, serine proteases, metalloproteinase, and neutral protease with nematicidal activities have been identified in *Brevibacillus laterosporus, Bacillus* sp*., Pseudoalteromonas tunicate, Serratia* sp., and *Stenotrophomonas maltophilia* (Qiuhong et al., 2006; Paiva et al., 2013; Salikin et al., 2021). Extracellular secreted proteases might assist bacterial infection by degrading the outer proteinaceous membrane of the cuticle (Cox et al., 1981). These proteases help bacterial strains in nutrient acquisition, resistance against host defense by modulating host proteins, and colonization of host by tissue invasion and damage (Matsumoto, 2004). In the current study, some bacterial enzymes were expressed in *E. coli*, and some of the proteases of *P. syringae* MB03 were found to be toxic against *C. elegans* (Table 6).

Gut colonization is another mechanism by which pathogens kill *C. elegans* (White et al., 2015; Ali et al., 2016). It is one of the mechanisms used by *P. aeruginosa* to kill *C. elegans* in the slow killing assay (Mahajan-Miklos et al., 1999). *P. syringae* also kills *C. elegans* by gut colonization (Ali et al., 2016). Gut colonization in the liquid killing assay (Figure 1) is contrary to *P. aeruginosa*, which implies this killing mechanism in the liquid killing assay (Kirienko et al., 2013, 2015).

The integrated utilization of various techniques resulted in the genome-wide prediction of bacterial virulence factors that required lethal host–pathogen interaction. For instance, transcriptomics and transposon insertion mutant library identified novel genes required for successful infection.

## Conclusion

In summary, *P. syringae* MB03 can kill *C. elegans* in the liquid assay via gut colonization. Comparative genomics revealed 156 unique genes and 115 potential nematicidal genes in the MB03 genome. The current study was performed using draft genome sequence of *P. syringae* MB03 hence, there is a possibility of identification more genes related to the virulence. Transcriptomics analysis showed that a variety of virulence genes were highly up-regulated. Furthermore, seven nematicidal genes were identified via screening of transposon insertion mutant library and bioassays. Total 27 nematicidal enzymes/proteins were identified based upon the activity of heterologously expressing strains of *E. coli*. The pathogenicity appeared to be a combinatorial action of various genes, including regulatory genes (signal transduction system and transcriptional regulators), genes related to locomotion (flagella proteins), and genes for nutrient acquisition (different metabolic proteins and catabolic enzymes).

## Data Availability Statement

The datasets presented in this study can be found in online repositories. The names of the repository/repositories and accession number(s) can be found in the article/Supplementary Material.

## Author Contributions

LL: conceptualization, funding acquisition, project administration, resources, supervision, and writing – review and editing. MA: formal analysis, investigation, and writing – original draft. TG, XY, AB, ZW, XS, and NA: validation and visualization. All authors: contributed to the article and approved the submitted version.

## Conflict of Interest

The authors declare that the research was conducted in the absence of any commercial or financial relationships that could be construed as a potential conflict of interest.

## Publisher’s Note

All claims expressed in this article are solely those of the authors and do not necessarily represent those of their affiliated organizations, or those of the publisher, the editors and the reviewers. Any product that may be evaluated in this article, or claim that may be made by its manufacturer, is not guaranteed or endorsed by the publisher.

## Footnotes

1
http://www.pseudomonas-syringae.org

2
http://www.paidb.re.kr/genome_pai.php?gma=NC_007005&m=g
