# Supplementary material for: Identification of the Genes of the Plant Pathogen Pseudomonas syringae MB03 Required for the Nematicidal Activity Against Caenorhabditis elegans Through an Integrated Approach
Source: Front Microbiol. 2022 Mar 9;13:826962. doi: 10.3389/fmicb.2022.826962 (PMC8959697; doi:10.3389/fmicb.2022.826962)
Supplement: Supplementary file 1 [file Data_Sheet_1.PDF]

Table S1. Genomic features of *P. syringae* MB03 (genome version LAGV01000000)

Attributes	Values
Genome size (bp)	5777225
Plasmid	0
Total genes	5026
rRNA genes	4
tRNA genes	53
Protein coding genes	4881
Number of contigs	76
Pseudogenes	40
