# Supplementary material for: Identification of the Genes of the Plant Pathogen Pseudomonas syringae MB03 Required for the Nematicidal Activity Against Caenorhabditis elegans Through an Integrated Approach
Source: Front Microbiol. 2022 Mar 9;13:826962. doi: 10.3389/fmicb.2022.826962 (PMC8959697; doi:10.3389/fmicb.2022.826962)
Supplement: Supplementary file 2 [file Data_Sheet_2.PDF]

Table S2. **Distribution of genes of selected strains of *P. syringae* under COG categories**.

COG Categories	Number of genes					
	MB03	B728a	CC1557	DC3000	SM	B64
Amino acid transport and metabolism	420	450	428	448	430	422
Carbohydrate transport and metabolism	258	257	253	264	255	258
Cell cycle control, cell division, chromosome partitioning	39	39	38	39	39	38
Cell motility	145	149	124	153	149	141
Cell wall/membrane/envelope biogenesis	250	258	228	250	252	246
Chromatin structure and dynamics	1	1		1	1	1
Coenzyme transport and metabolism	216	213	214	218	214	212
Defense mechanisms	90	88	73	91	88	86
Energy production and conversion	220	214	215	222	214	216
Extracellular structures	36	37	32	38	38	33
Function unknown	205	215	221	218	212	205
General function prediction only	371	369	341	388	364	365
Inorganic ion transport and metabolism	269	284	267	277	268	266
Intracellular trafficking, secretion, and vesicular transport	111	100	118	109	118	105
Lipid transport and metabolism	167	177	164	183	164	164
Mobilome: prophages, transposons	24	52	41	193	37	36
Nucleotide transport and metabolism	89	89	86	84	90	91
Posttranslational modification, protein turnover, chaperones	150	157	146	149	157	151
Replication, recombination and repair	116	126	117	163	131	118
RNA processing and modification	1	1	1	1	1	1
Secondary metabolites biosynthesis, transport and catabolism	112	116	103	113	112	115
Signal transduction mechanisms	282	292	259	300	286	271
Transcription	301	312	311	311	306	309
Translation, ribosomal structure and biogenesis	247	246	235	239	249	248
