# Supplementary material for: Identification of the Genes of the Plant Pathogen Pseudomonas syringae MB03 Required for the Nematicidal Activity Against Caenorhabditis elegans Through an Integrated Approach
Source: Front Microbiol. 2022 Mar 9;13:826962. doi: 10.3389/fmicb.2022.826962 (PMC8959697; doi:10.3389/fmicb.2022.826962)
Supplement: Supplementary file 3 [file Data_Sheet_3.PDF]

Table S3. Strain specific genes of *P. syringae* MB03 when compared with other strains. [a]

Absent in CC1557		Absent in B728a		Absent in SM		Absent in B64		Absent in HS191		Absent in DC3000, CC1557, B728a, SM and B64		Absent in HS191, DC3000, CC1557, B728a, SM, B64	
VT47_00160	hypothetical protein	VT47_00270	hypothetical protein	VT47_00395	hypothetical protein	VT47_00395	hypothetical protein	VT47_00395	hypothetical protein	VT47_00395	hypothetical protein	VT47_00395	hypothetical protein
VT47_00165	hypothetical protein	VT47_00395	hypothetical protein	VT47_00415	alpha-ketoglutarate transporter	VT47_00420	LuxR family transcriptional	VT47_00415	alpha-ketoglutarate transporter	VT47_00420	LuxR family transcriptional regulator	VT47_00430	anthranilate synthase
VT47_00395	hypothetical protein	VT47_00420	LuxR family transcriptional regulator	VT47_00420	LuxR family transcriptional regulator	VT47_00425	anthranilate synthase subunit II	VT47_00420	LuxR family transcriptional	VT47_00430	anthranilate synthase	VT47_00435	hypothetical protein
VT47_00420	LuxR family transcriptional regulator	VT47_00425	anthranilate synthase subunit II	VT47_00425	anthranilate synthase subunit II	VT47_00430	anthranilate synthase	VT47_00430	anthranilate synthase	VT47_00435	hypothetical protein	VT47_00440	alpha/beta hydrolase
VT47_00425	anthranilate synthase subunit II	VT47_00430	anthranilate synthase	VT47_00430	anthranilate synthase	VT47_00435	hypothetical protein	VT47_00435	hypothetical protein	VT47_00440	alpha/beta hydrolase	VT47_00445	hypothetical protein
VT47_00430	anthranilate synthase	VT47_00435	hypothetical protein	VT47_00435	hypothetical protein	VT47_00440	alpha/beta hydrolase	VT47_00440	alpha/beta hydrolase	VT47_00445	hypothetical protein	VT47_00450	FMN reductase
VT47_00435	hypothetical protein	VT47_00440	alpha/beta hydrolase	VT47_00440	alpha/beta hydrolase	VT47_00445	hypothetical protein	VT47_00445	hypothetical protein	VT47_00450	FMN reductase	VT47_00455	transcriptional regulator
VT47_00440	alpha/beta hydrolase	VT47_00445	hypothetical protein	VT47_00445	hypothetical protein	VT47_00450	FMN reductase	VT47_00450	FMN reductase	VT47_00455	transcriptional regulator	VT47_00460	hypothetical protein
VT47_00445	hypothetical protein	VT47_00450	FMN reductase	VT47_00450	FMN reductase	VT47_00455	transcriptional regulator	VT47_00455	transcriptional regulator	VT47_00460	hypothetical protein	VT47_00465	coenzyme F390 synthetase
VT47_00450	FMN reductase	VT47_00455	transcriptional regulator	VT47_00455	transcriptional regulator	VT47_00460	hypothetical protein	VT47_00460	hypothetical protein	VT47_00465	coenzyme F390 synthetase	VT47_00510	hypothetical protein
VT47_00455	transcriptional regulator	VT47_00460	hypothetical protein	VT47_00460	hypothetical protein	VT47_00465	coenzyme F390 synthetase	VT47_00465	coenzyme F390 synthetase	VT47_00510	hypothetical protein	VT47_00755	hypothetical protein
VT47_00460	hypothetical protein	VT47_00465	coenzyme F390 synthetase	VT47_00465	coenzyme F390 synthetase	VT47_00510	hypothetical protein	VT47_00500	acetyltransferase	VT47_00560	xylose isomerase	VT47_00760	hypothetical protein
VT47_00465	coenzyme F390 synthetase	VT47_00470	TetR family transcriptional regulator	VT47_00500	acetyltransferase	VT47_00555	LysR family transcriptional	VT47_00505	hypothetical protein	VT47_00755	hypothetical protein	VT47_00915	hypothetical protein
VT47_00470	TetR family transcriptional regulator	VT47_00475	NmrA family protein	VT47_00505	hypothetical protein	VT47_00560	xylose isomerase	VT47_00510	hypothetical protein	VT47_00760	hypothetical protein	VT47_00930	hypothetical protein
VT47_00475	NmrA family protein	VT47_00480	epoxide hydrolase	VT47_00510	hypothetical protein	VT47_00565	hypothetical protein	VT47_00515	hypothetical protein	VT47_00915	hypothetical protein	VT47_00940	hypothetical protein
VT47_00480	epoxide hydrolase	VT47_00500	acetyltransferase	VT47_00515	hypothetical protein	VT47_00570	hypothetical protein	VT47_00520	DNA mismatch repair protein MutT	VT47_00930	hypothetical protein	VT47_00950	hypothetical protein
VT47_00485	lipoprotein	VT47_00505	hypothetical protein	VT47_00520	DNA mismatch repair protein MutT	VT47_00755	hypothetical protein	VT47_00535	deoxyribose mutarotase	VT47_00940	hypothetical protein	VT47_00955	AbrB family transcriptional regulator
VT47_00505	hypothetical protein	VT47_00510	hypothetical protein	VT47_00555	LysR family transcriptional regulator	VT47_00760	hypothetical protein	VT47_00755	hypothetical protein	VT47_00950	hypothetical protein	VT47_00960	hypothetical protein
VT47_00510	hypothetical protein	VT47_00515	hypothetical protein	VT47_00560	xylose isomerase	VT47_00900	oxidoreductase	VT47_00760	hypothetical protein	VT47_00955	AbrB family transcriptional regulator	VT47_00965	hypothetical protein
VT47_00515	hypothetical protein	VT47_00520	DNA mismatch repair protein MutT	VT47_00565	hypothetical protein	VT47_00905	GCN5 family acetyltransferase	VT47_00915	hypothetical protein	VT47_00960	hypothetical protein	VT47_00970	hypothetical protein
VT47_00520	DNA mismatch repair protein MutT	VT47_00560	xylose isomerase	VT47_00570	hypothetical protein	VT47_00915	hypothetical protein	VT47_00930	hypothetical protein	VT47_00965	hypothetical protein	VT47_00975	hypothetical protein
VT47_00555	LysR family transcriptional regulator	VT47_00565	hypothetical protein	VT47_00605	hypothetical protein	VT47_00925	hypothetical protein	VT47_00935	NAD-dependent deacetylase	VT47_00970	hypothetical protein	VT47_00980	hypothetical protein

VT47_00560	xylose isomerase	VT47_00570	hypothetical protein	VT47_00755	hypothetical protein	VT47_00930	hypothetical protein	VT47_00940	hypothetical protein	VT47_00975	hypothetical protein	VT47_00985	hypothetical protein
VT47_00565	hypothetical protein	VT47_00755	hypothetical protein	VT47_00760	hypothetical protein	VT47_00935	NAD-dependent deacetylase	VT47_00950	hypothetical protein	VT47_00980	hypothetical protein	VT47_00990	hypothetical protein
VT47_00570	hypothetical protein	VT47_00760	hypothetical protein	VT47_00890	hypothetical protein	VT47_00940	hypothetical protein	VT47_00955	AbrB family transcriptional	VT47_00985	hypothetical protein	VT47_03115	nuclease
VT47_00575	hypothetical protein	VT47_00800	glutathione S-transferase	VT47_00915	hypothetical protein	VT47_00950	hypothetical protein	VT47_00960	hypothetical protein	VT47_00990	hypothetical protein	VT47_03125	metal-dependent hydrolase
VT47_00580	membrane protein	VT47_00905	GCN5 family acetyltransferase	VT47_00925	hypothetical protein	VT47_00955	AbrB family transcriptional	VT47_00965	hypothetical protein	VT47_01145	hypothetical protein	VT47_03130	restriction endonuclease subunit R
VT47_00595	hypothetical protein	VT47_00915	hypothetical protein	VT47_00930	hypothetical protein	VT47_00960	hypothetical protein	VT47_00970	hypothetical protein	VT47_03115	nuclease	VT47_03140	hypothetical protein
VT47_00755	hypothetical protein	VT47_00925	hypothetical protein	VT47_00935	NAD-dependent deacetylase	VT47_00965	hypothetical protein	VT47_00975	hypothetical protein	VT47_03125	metal-dependent hydrolase	VT47_03145	restriction endonuclease subunit M
VT47_00760	hypothetical protein	VT47_00930	hypothetical protein	VT47_00940	hypothetical protein	VT47_00970	hypothetical protein	VT47_00980	hypothetical protein	VT47_03130	restriction endonuclease subunit R	VT47_03150	hypothetical protein
VT47_00890	hypothetical protein	VT47_00940	hypothetical protein	VT47_00950	hypothetical protein	VT47_00975	hypothetical protein	VT47_00985	hypothetical protein	VT47_03140	hypothetical protein	VT47_03720	hypothetical protein
VT47_00905	GCN5 family acetyltransferase	VT47_00950	hypothetical protein	VT47_00955	AbrB family transcriptional regulator	VT47_00980	hypothetical protein	VT47_00990	hypothetical protein	VT47_03145	restriction endonuclease subunit M	VT47_03725	hypothetical protein
VT47_00915	hypothetical protein	VT47_00955	AbrB family transcriptional regulator	VT47_00960	hypothetical protein	VT47_00985	hypothetical protein	VT47_00995	hypothetical protein	VT47_03150	hypothetical protein	VT47_03730	chromosome segregation protein SMC
VT47_00925	hypothetical protein	VT47_00960	hypothetical protein	VT47_00965	hypothetical protein	VT47_00990	hypothetical protein	VT47_01000	transposase	VT47_03720	hypothetical protein	VT47_03735	helicase UvrD
VT47_00930	hypothetical protein	VT47_00965	hypothetical protein	VT47_00970	hypothetical protein	VT47_00995	hypothetical protein	VT47_01735	hypothetical protein	VT47_03725	hypothetical protein	VT47_03740	hypothetical protein
VT47_00935	NAD-dependent deacetylase	VT47_00970	hypothetical protein	VT47_00975	hypothetical protein	VT47_01000	transposase	VT47_02750	hypothetical protein	VT47_03730	chromosome segregation protein SMC	VT47_04525	glycosyl transferase family 2
VT47_00940	hypothetical protein	VT47_00975	hypothetical protein	VT47_00980	hypothetical protein	VT47_01145	hypothetical protein	VT47_03115	nuclease	VT47_03735	helicase UvrD	VT47_04530	4-amino-4-deoxy-L-arabinose-phospho-UDP
VT47_00950	hypothetical protein	VT47_00980	hypothetical protein	VT47_00985	hypothetical protein	VT47_01545	energy transducer TonB	VT47_03125	metal-dependent hydrolase	VT47_03740	hypothetical protein	VT47_04535	phosphoglycerol transferase
VT47_00955	AbrB family transcriptional regulator	VT47_00985	hypothetical protein	VT47_00990	hypothetical protein	VT47_01555	hypothetical protein	VT47_03130	restriction endonuclease subunit R	VT47_03745	hypothetical protein	VT47_04565	hypothetical protein
VT47_00960	hypothetical protein	VT47_00990	hypothetical protein	VT47_00995	hypothetical protein	VT47_01560	bacteriocin immunity protein	VT47_03140	hypothetical protein	VT47_04525	glycosyl transferase family 2	VT47_07020	hypothetical protein
VT47_00965	hypothetical protein	VT47_00995	hypothetical protein	VT47_01000	transposase	VT47_01565	HNH endonuclease	VT47_03145	restriction endonuclease subunit	VT47_04530	4-amino-4-deoxy-L-arabinose-phospho-UDP	VT47_07035	hypothetical protein
VT47_00970	hypothetical protein	VT47_01000	transposase	VT47_01145	hypothetical protein	VT47_01660	glutamyl-tRNA amidotransferase	VT47_03150	hypothetical protein	VT47_04535	phosphoglycerol transferase	VT47_07040	hypothetical protein
VT47_00975	hypothetical protein	VT47_01145	hypothetical protein	VT47_01735	hypothetical protein	VT47_01670	hypothetical protein	VT47_03450	hypothetical protein	VT47_04565	hypothetical protein	VT47_07130	hypothetical protein
VT47_00980	hypothetical protein	VT47_01320	membrane protein	VT47_02515	glycosyltransferase sugar-binding domain-	VT47_02750	hypothetical protein	VT47_03720	hypothetical protein	VT47_05605	hypothetical protein	VT47_07165	aspartate carbamoyltransferase
VT47_00985	hypothetical protein	VT47_01430	membrane protein	VT47_02815	flagellar motor protein MotA	VT47_03105	DNA-binding protein	VT47_03725	hypothetical protein	VT47_07020	hypothetical protein	VT47_07175	methyltransferase type 11
VT47_00990	hypothetical protein	VT47_01550	energy transducer TonB	VT47_02990	amino acid ABC transporter substrate-	VT47_03110	hypothetical protein	VT47_03730	chromosome segregation protein	VT47_07035	hypothetical protein	VT47_07195	hypothetical protein
VT47_00995	hypothetical protein	VT47_01665	glutamyl-tRNA amidotransferase	VT47_03115	nuclease	VT47_03115	nuclease	VT47_03735	helicase UvrD	VT47_07040	hypothetical protein	VT47_08395	hypothetical protein

VT47_01000	transposase	VT47_01860	membrane protein	VT47_03125	metal-dependent hydrolase	VT47_03125	metal-dependent hydrolase	VT47_03740	hypothetical protein	VT47_07130	hypothetical protein	VT47_08400	hypothetical protein
VT47_01145	hypothetical protein	VT47_02060	amidase	VT47_03130	restriction endonuclease subunit R	VT47_03130	restriction endonuclease subunit [illegible]	VT47_03940	pilus assembly protein PilA	VT47_07165	aspartate carbamoyltransferase [illegible]	VT47_08635	hypothetical protein
VT47_01265	hypothetical protein	VT47_02090	signal peptide protein	VT47_03135	hypothetical protein	VT47_03135	hypothetical protein	VT47_04490	glycosyl transferase family 1	VT47_07175	methyltransferase type 11	VT47_08640	hypothetical protein
VT47_01270	AsnC family transcriptional regulator	VT47_02105	peptidase S1	VT47_03140	hypothetical protein	VT47_03140	hypothetical protein	VT47_04495	GDP-6-deoxy-D-lyxo-4-hexulose reductase	VT47_07195	hypothetical protein	VT47_08645	hypothetical protein
VT47_01430	membrane protein	VT47_02530	actin	VT47_03145	restriction endonuclease subunit M	VT47_03145	restriction endonuclease subunit [illegible]	VT47_04505	ABC transporter	VT47_07955	hypothetical protein	VT47_08655	hypothetical protein
VT47_01465	isoprenoid biosynthesis protein	VT47_02750	hypothetical protein	VT47_03150	hypothetical protein	VT47_03150	hypothetical protein	VT47_04515	SAM-dependent methlyltransferase	VT47_08395	hypothetical protein	VT47_09555	hypothetical protein
VT47_01545	energy transducer TonB	VT47_03105	DNA-binding protein	VT47_03675	DNA topoisomerase III	VT47_03675	DNA topoisomerase III	VT47_04520	glycosyl transferase family 1	VT47_08400	hypothetical protein	VT47_09795	hypothetical protein
VT47_01550	energy transducer TonB	VT47_03110	hypothetical protein	VT47_03715	hypothetical protein	VT47_03715	hypothetical protein	VT47_04525	glycosyl transferase family 2	VT47_08635	hypothetical protein	VT47_09800	hypothetical protein
VT47_01555	hypothetical protein	VT47_03115	nuclease	VT47_03720	hypothetical protein	VT47_03720	hypothetical protein	VT47_04530	4-amino-4-deoxy-L-arabinose-phospho- [illegible]	VT47_08640	hypothetical protein	VT47_09805	ProQ activator of osmoprotectant
VT47_01560	bacteriocin immunity protein	VT47_03125	metal-dependent hydrolase	VT47_03725	hypothetical protein	VT47_03725	hypothetical protein	VT47_04535	phosphoglycerol transferase	VT47_08645	hypothetical protein	VT47_09810	hypothetical protein
VT47_01565	HNH endonuclease	VT47_03130	restriction endonuclease subunit R	VT47_03730	chromosome segregation protein [illegible]	VT47_03730	chromosome segregation protein [illegible]	VT47_04560	glycosyltransferase	VT47_08650	hypothetical protein	VT47_09815	hypothetical protein
VT47_01650	lipase	VT47_03135	hypothetical protein	VT47_03735	helicase UvrD	VT47_03735	helicase UvrD	VT47_04565	hypothetical protein	VT47_08655	hypothetical protein	VT47_09820	hypothetical protein
VT47_01665	glutamyl-tRNA amidotransferase	VT47_03140	hypothetical protein	VT47_03740	hypothetical protein	VT47_03740	hypothetical protein	VT47_04570	HAD superfamily hydrolase-like protein	VT47_09555	hypothetical protein	VT47_09825	hypothetical protein
VT47_01700	hypothetical protein	VT47_03145	restriction endonuclease subunit M	VT47_03745	hypothetical protein	VT47_03745	hypothetical protein	VT47_04575	UDP-glucose 6-dehydrogenase	VT47_09795	hypothetical protein	VT47_09840	hypothetical protein
VT47_01705	hypothetical protein	VT47_03150	hypothetical protein	VT47_03755	transposase	VT47_03755	transposase	VT47_04580	glycosyl transferase	VT47_09800	hypothetical protein	VT47_09850	hypothetical protein
VT47_01715	hypothetical protein	VT47_03510	membrane protein	VT47_03760	hypothetical protein	VT47_03760	hypothetical protein	VT47_04585	glycosyl transferase	VT47_09805	ProQ activator of osmoprotectant	VT47_09860	hypothetical protein
VT47_01735	hypothetical protein	VT47_03675	DNA topoisomerase III	VT47_04300	GDP-D-mannose dehydratase	VT47_03940	pilus assembly protein PilA	VT47_04590	hemolysin	VT47_09810	hypothetical protein	VT47_09865	hypothetical protein
VT47_02045	cell division protein	VT47_03680	LysR family transcriptional regulator	VT47_04305	GDP-6-deoxy-D-lyxo-4-hexulose reductase	VT47_04110	MFS transporter	VT47_04595	hypothetical protein	VT47_09815	hypothetical protein	VT47_09875	hypothetical protein
VT47_02050	glutamate synthase	VT47_03685	LuxR family transcriptional regulator	VT47_04525	glycosyl transferase family 2	VT47_04255	acyltransferase	VT47_04605	hemolysin secretion protein D	VT47_09820	hypothetical protein	VT47_09880	hypothetical protein
VT47_02060	amidase	VT47_03695	lysine transporter LysE	VT47_04530	4-amino-4-deoxy-L-arabinose-phospho-UDP [illegible]	VT47_04300	GDP-D-mannose dehydratase	VT47_04610	channel protein TolC	VT47_09825	hypothetical protein	VT47_10670	hypothetical protein
VT47_02065	hypothetical protein	VT47_03715	hypothetical protein	VT47_04535	phosphoglycerol transferase	VT47_04305	GDP-6-deoxy-D-lyxo-4-hexulose reductase	VT47_04615	glycosyl transferase family 1	VT47_09840	hypothetical protein	VT47_10675	hypothetical protein
VT47_02090	signal peptide protein	VT47_03720	hypothetical protein	VT47_04565	hypothetical protein	VT47_04525	glycosyl transferase family 2	VT47_05045	hypothetical protein	VT47_09850	hypothetical protein	VT47_11105	hypothetical protein
VT47_02105	peptidase S1	VT47_03725	hypothetical protein	VT47_05185	YD repeat-containing protein	VT47_04530	4-amino-4-deoxy-L-arabinose-phospho- [illegible]	VT47_06000	avirulence protein	VT47_09860	hypothetical protein	VT47_11895	hypothetical protein
VT47_02125	3-oxoacyl-ACP synthase	VT47_03730	chromosome segregation protein SMC	VT47_05320	HxlR family transcriptional regulator	VT47_04535	phosphoglycerol transferase	VT47_06005	type III secretion chaperone CesT	VT47_09865	hypothetical protein	VT47_12030	hypothetical protein
VT47_02145	membrane protein	VT47_03735	helicase UvrD	VT47_05605	hypothetical protein	VT47_04555	spore coat protein	VT47_06010	hypothetical protein	VT47_09875	hypothetical protein	VT47_12525	syringomycin synthetase

VT47_02515	glycosyltransferase sugar-binding domain-	VT47_03740	hypothetical protein	VT47_05680	hypothetical protein	VT47_04560	glycosyltransferase	VT47_06205	dihydropteridine reductase	VT47_09880	hypothetical protein	VT47_12565	hypothetical protein
VT47_02520	ADP-ribosylating toxin	VT47_03745	hypothetical protein	VT47_05995	hypothetical protein	VT47_04565	hypothetical protein	VT47_06215	tautomerase	VT47_10410	integrase	VT47_12595	amino acid adenylation
VT47_02530	actin	VT47_03755	transposase	VT47_06000	avirulence protein	VT47_04570	HAD superfamily hydrolase-like protein	VT47_06225	hypothetical protein	VT47_10415	hypothetical protein	VT47_12710	hypothetical protein
VT47_02690	heptose 1-phosphate adenyltransferase	VT47_03760	hypothetical protein	VT47_06005	type III secretion chaperone CesT	VT47_04575	UDP-glucose 6-dehydrogenase	VT47_06230	hypothetical protein	VT47_10670	hypothetical protein	VT47_12715	hypothetical protein
VT47_03055	ABC transporter permease	VT47_03835	haloacid dehalogenase	VT47_06010	hypothetical protein	VT47_05605	hypothetical protein	VT47_06255	NGG1p interacting factor 3 protein, NIF3	VT47_10675	hypothetical protein	VT47_12725	hypothetical protein
VT47_03060	ABC transporter ATP-binding protein	VT47_04300	GDP-D-mannose dehydratase	VT47_06225	hypothetical protein	VT47_06010	hypothetical protein	VT47_06555	beta-(1-3)-glucosyl transferase	VT47_11105	hypothetical protein	VT47_12730	hypothetical protein
VT47_03065	glycosyl transferase family 2	VT47_04305	GDP-6-deoxy-D-lyxo-4-hexulose reductase	VT47_06555	beta-(1-3)-glucosyl transferase	VT47_06200	chemotaxis protein CheY	VT47_07020	hypothetical protein	VT47_11895	hypothetical protein	VT47_12735	hypothetical protein
VT47_03070	phosphodiesterase	VT47_04525	glycosyl transferase family 2	VT47_07020	hypothetical protein	VT47_06205	dihydropteridine reductase	VT47_07025	Clp protease	VT47_12030	hypothetical protein	VT47_12760	hypothetical protein
VT47_03075	HAD family hydrolase	VT47_04530	4-amino-4-deoxy-L-arabinose-phospho-UDP	VT47_07035	hypothetical protein	VT47_06210	2-nitropropane dioxygenase	VT47_07030	histidine kinase	VT47_12525	syringomycin synthetase	VT47_12775	hypothetical protein
VT47_03080	glycosyl transferase family 2	VT47_04535	phosphoglycerol transferase	VT47_07040	hypothetical protein	VT47_06215	tautomerase	VT47_07035	hypothetical protein	VT47_12565	hypothetical protein	VT47_12780	hypothetical protein
VT47_03085	hypothetical protein	VT47_04560	glycosyltransferase	VT47_07130	hypothetical protein	VT47_06555	beta-(1-3)-glucosyl transferase	VT47_07040	hypothetical protein	VT47_12595	amino acid adenylation	VT47_13240	regulatory protein
VT47_03090	hypothetical protein	VT47_04565	hypothetical protein	VT47_07135	hypothetical protein	VT47_07005	integrase	VT47_07045	general stress protein	VT47_12710	hypothetical protein	VT47_13335	hypothetical protein
VT47_03095	hypothetical protein	VT47_04570	HAD superfamily hydrolase-like protein	VT47_07165	aspartate carbamoyltransferase	VT47_07020	hypothetical protein	VT47_07055	cyclase/dehydrase	VT47_12715	hypothetical protein	VT47_13365	hypothetical protein
VT47_03100	WavE lipopolysaccharide	VT47_04575	UDP-glucose 6-dehydrogenase	VT47_07175	methyltransferase type 11	VT47_07025	Clp protease	VT47_07060	hypothetical protein	VT47_12725	hypothetical protein	VT47_14285	hypothetical protein
VT47_03105	DNA-binding protein	VT47_04975	hypothetical protein	VT47_07195	hypothetical protein	VT47_07030	histidine kinase	VT47_07065	chemotaxis protein	VT47_12730	hypothetical protein	VT47_14290	hypothetical protein
VT47_03110	hypothetical protein	VT47_05120	hypothetical protein	VT47_07210	hypothetical protein	VT47_07035	hypothetical protein	VT47_07075	chemotaxis protein	VT47_12735	hypothetical protein	VT47_14295	hypothetical protein
VT47_03115	nuclease	VT47_05125	peptidase	VT47_07225	integrase	VT47_07040	hypothetical protein	VT47_07080	lipoprotein	VT47_12760	hypothetical protein	VT47_14300	hypothetical protein
VT47_03125	metal-dependent hydrolase	VT47_05130	membrane protein	VT47_07235	histidine kinase	VT47_07045	general stress protein	VT47_07090	hypothetical protein	VT47_12775	hypothetical protein	VT47_14305	hypothetical protein
VT47_03130	restriction endonuclease subunit R	VT47_05605	hypothetical protein	VT47_07295	site-specific recombinase	VT47_07050	alcohol dehydrogenase	VT47_07100	ompetence-damaged protein	VT47_12780	hypothetical protein	VT47_15015	hypothetical protein
VT47_03135	hypothetical protein	VT47_05680	hypothetical protein	VT47_07625	dehydrogenase	VT47_07055	cyclase/dehydrase	VT47_07125	phenazine biosynthesis protein PhzF	VT47_12965	nucleoside hydrolase	VT47_15235	hypothetical protein
VT47_03140	hypothetical protein	VT47_05695	DNA mismatch repair protein MutT	VT47_07955	hypothetical protein	VT47_07060	hypothetical protein	VT47_07130	hypothetical protein	VT47_13240	regulatory protein	VT47_15910	hypothetical protein
VT47_03145	restriction endonuclease subunit M	VT47_05760	ester cyclase	VT47_08010	LuxR family transcriptional regulator	VT47_07065	chemotaxis protein	VT47_07165	aspartate carbamoyltransferase	VT47_13260	hypothetical protein	VT47_15920	hypothetical protein
VT47_03150	hypothetical protein	VT47_05790	lipase	VT47_08015	fatty acid desaturase	VT47_07070	hypothetical protein	VT47_07170	MFS transporter	VT47_13335	hypothetical protein	VT47_16075	hypothetical protein
VT47_03285	AraC family transcriptional regulator	VT47_05795	transketolase	VT47_08020	thioester reductase	VT47_07075	chemotaxis protein	VT47_07175	methyltransferase type 11	VT47_13365	hypothetical protein	VT47_17915	hypothetical protein
VT47_03325	LysR family transcriptional regulator	VT47_05800	transketolase	VT47_08025	amino acid adenylation	VT47_07080	lipoprotein	VT47_07180	glucose starvation-inducible protein B	VT47_14285	hypothetical protein	VT47_18080	hypothetical protein

VT47_03445	DNA mismatch repair protein MutT	VT47_05805	MFS transporter permease	VT47_08030	MFS transporter	VT47_07090	hypothetical protein	VT47_07185	general stress protein	VT47_14290	hypothetical protein	VT47_18120	hypothetical protein
VT47_03450	hypothetical protein	VT47_05810	short-chain dehydrogenase	VT47_08340	hypothetical protein	VT47_07100	ompetence-damaged protein	VT47_07195	hypothetical protein	VT47_14295	hypothetical protein	VT47_18125	hypothetical protein
VT47_03675	DNA topoisomerase III	VT47_05815	LysR family transcriptional regulator	VT47_08345	hypothetical protein	VT47_07105	transposase	VT47_07230	N-methyl-transferase related protein	VT47_14300	hypothetical protein	VT47_18135	hypothetical protein
VT47_03715	hypothetical protein	VT47_05995	hypothetical protein	VT47_08395	hypothetical protein	VT47_07110	transposase	VT47_07295	site-specific recombinase	VT47_14305	hypothetical protein	VT47_18145	hypothetical protein
VT47_03720	hypothetical protein	VT47_06160	hypothetical protein	VT47_08400	hypothetical protein	VT47_07125	phenazine biosynthesis protein PhzF	VT47_07875	hypothetical protein	VT47_15015	hypothetical protein	VT47_18150	hypothetical protein
VT47_03725	hypothetical protein	VT47_06200	chemotaxis protein CheY	VT47_08405	hypothetical protein	VT47_07130	hypothetical protein	VT47_08395	hypothetical protein	VT47_15235	hypothetical protein	VT47_18160	hypothetical protein
VT47_03730	chromosome segregation protein	VT47_06205	dihydropteridine reductase	VT47_08435	ArsR family transcriptional regulator	VT47_07135	hypothetical protein	VT47_08400	hypothetical protein	VT47_15910	hypothetical protein	VT47_19025	hypothetical protein
VT47_03735	helicase UvrD	VT47_06210	2-nitropropane dioxygenase	VT47_08635	hypothetical protein	VT47_07140	membrane protein	VT47_08635	hypothetical protein	VT47_15920	hypothetical protein	VT47_20140	hypothetical protein
VT47_03740	hypothetical protein	VT47_06215	tautomerase	VT47_08640	hypothetical protein	VT47_07145	hypothetical protein	VT47_08640	hypothetical protein	VT47_16075	hypothetical protein	VT47_20160	hypothetical protein
VT47_03745	hypothetical protein	VT47_06225	hypothetical protein	VT47_08645	hypothetical protein	VT47_07150	conjugal transfer protein	VT47_08645	hypothetical protein	VT47_17915	hypothetical protein	VT47_20170	hypothetical protein
VT47_03755	transposase	VT47_06585	hypothetical protein	VT47_08650	hypothetical protein	VT47_07155	conjugal transfer protein	VT47_08655	hypothetical protein	VT47_18080	hypothetical protein	VT47_20175	hypothetical protein
VT47_03760	hypothetical protein	VT47_07020	hypothetical protein	VT47_08655	hypothetical protein	VT47_07165	aspartate carbamoyltransferase	VT47_08965	cupin	VT47_18120	hypothetical protein	VT47_20190	Appr-1-p processing protein
VT47_03840	membrane protein	VT47_07025	Clp protease	VT47_09555	hypothetical protein	VT47_07170	MFS transporter	VT47_08970	LysR family transcriptional	VT47_18125	hypothetical protein	VT47_20690	hypothetical protein
VT47_03845	hypothetical protein	VT47_07030	histidine kinase	VT47_09660	hypothetical protein	VT47_07175	methyltransferase type 11	VT47_09555	hypothetical protein	VT47_18135	hypothetical protein	VT47_20695	hypothetical protein
VT47_03905	hypothetical protein	VT47_07035	hypothetical protein	VT47_09680	LuxR family transcriptional regulator	VT47_07180	glucose starvation-inducible protein B	VT47_09785	transposase	VT47_18145	hypothetical protein	VT47_20700	hypothetical protein
VT47_03910	hypothetical protein	VT47_07040	hypothetical protein	VT47_09780	transposase	VT47_07185	general stress protein	VT47_09795	hypothetical protein	VT47_18150	hypothetical protein	VT47_20720	hypothetical protein
VT47_03940	pilus assembly protein PilA	VT47_07045	general stress protein	VT47_09795	hypothetical protein	VT47_07195	hypothetical protein	VT47_09800	hypothetical protein	VT47_18160	hypothetical protein	VT47_20780	hypothetical protein
VT47_04255	acyltransferase	VT47_07050	alcohol dehydrogenase	VT47_09800	hypothetical protein	VT47_07210	hypothetical protein	VT47_09805	ProQ activator of osmoprotectant	VT47_19025	hypothetical protein	VT47_21330	hypothetical protein
VT47_04285	chemotaxis protein	VT47_07055	cyclase/dehydrase	VT47_09805	ProQ activator of osmoprotectant	VT47_07220	hypothetical protein	VT47_09810	hypothetical protein	VT47_20140	hypothetical protein	VT47_21625	hypothetical protein
VT47_04350	GCN5 family acetyltransferase	VT47_07060	hypothetical protein	VT47_09810	hypothetical protein	VT47_07225	integrase	VT47_09815	hypothetical protein	VT47_20160	hypothetical protein	VT47_21630	hypothetical protein
VT47_04395	plasmid maintenance protein	VT47_07065	chemotaxis protein	VT47_09815	hypothetical protein	VT47_07235	histidine kinase	VT47_09820	hypothetical protein	VT47_20170	hypothetical protein	VT47_21635	thiamine biosynthesis protein ThiF
VT47_04400	antitoxin	VT47_07070	hypothetical protein	VT47_09820	hypothetical protein	VT47_07295	site-specific recombinase	VT47_09825	hypothetical protein	VT47_20175	hypothetical protein	VT47_21640	hypothetical protein
VT47_04450	chemotaxis protein	VT47_07075	chemotaxis protein	VT47_09825	hypothetical protein	VT47_07520	Ice nucleation protein	VT47_09830	hypothetical protein	VT47_20190	Appr-1-p processing protein	VT47_21645	patatin
VT47_04465	lactate dehydrogenase	VT47_07080	lipoprotein	VT47_09830	hypothetical protein	VT47_07625	dehydrogenase	VT47_09835	serine/threonine protein phosphatase	VT47_20690	hypothetical protein	VT47_21650	hypothetical protein
VT47_04470	LysR family transcriptional regulator	VT47_07090	hypothetical protein	VT47_09835	serine/threonine protein phosphatase	VT47_07955	hypothetical protein	VT47_09840	hypothetical protein	VT47_20695	hypothetical protein	VT47_21655	exonuclease V subunit alpha

VT47_04490	glycosyl transferase family 1	VT47_07100	ompetence-damaged protein	VT47_09840	hypothetical protein	VT47_08340	hypothetical protein	VT47_09850	hypothetical protein	VT47_20700	hypothetical protein	VT47_22245	hypothetical protein
VT47_04495	GDP-6-deoxy-D-lyxo-4-hexulose reductase	VT47_07105	transposase	VT47_09850	hypothetical protein	VT47_08395	hypothetical protein	VT47_09855	hypothetical protein	VT47_20720	hypothetical protein	VT47_22250	hypothetical protein
VT47_04500	GDP-D-mannose dehydratase	VT47_07125	phenazine biosynthesis protein PhzF	VT47_09855	hypothetical protein	VT47_08400	hypothetical protein	VT47_09860	hypothetical protein	VT47_20780	hypothetical protein	VT47_22310	phage replication protein
VT47_04505	ABC transporter	VT47_07130	hypothetical protein	VT47_09860	hypothetical protein	VT47_08405	hypothetical protein	VT47_09865	hypothetical protein	VT47_21330	hypothetical protein	VT47_22330	hypothetical protein
VT47_04510	sugar ABC transporter ATP-binding protein	VT47_07135	hypothetical protein	VT47_09865	hypothetical protein	VT47_08635	hypothetical protein	VT47_09870	hypothetical protein	VT47_21625	hypothetical protein	VT47_22380	integrase
VT47_04515	SAM-dependent methlyltransferase	VT47_07165	aspartate carbamoyltransferase [illegible]	VT47_09870	hypothetical protein	VT47_08640	hypothetical protein	VT47_09875	hypothetical protein	VT47_21630	hypothetical protein	VT47_22625	hypothetical protein
VT47_04520	glycosyl transferase family 1	VT47_07170	MFS transporter	VT47_09875	hypothetical protein	VT47_08645	hypothetical protein	VT47_09880	hypothetical protein	VT47_21635	thiamine biosynthesis protein ThiF	VT47_23050	hypothetical protein
VT47_04525	glycosyl transferase family 2	VT47_07175	methyltransferase type 11	VT47_09880	hypothetical protein	VT47_08650	hypothetical protein	VT47_10290	hypothetical protein	VT47_21640	hypothetical protein	VT47_23165	hypothetical protein
VT47_04530	4-amino-4-deoxy-L-arabinose-phospho- [illegible]	VT47_07180	glucose starvation-inducible protein B	VT47_10115	histidine kinase	VT47_08655	hypothetical protein	VT47_10425	hypothetical protein	VT47_21645	patatin	VT47_23170	hypothetical protein
VT47_04535	phosphoglycerol transferase	VT47_07185	general stress protein	VT47_10410	integrase	VT47_08950	hypothetical protein	VT47_10430	hypothetical protein	VT47_21650	hypothetical protein	VT47_23175	hypothetical protein
VT47_04555	spore coat protein	VT47_07195	hypothetical protein	VT47_10415	hypothetical protein	VT47_08965	cupin	VT47_10445	prophage PssSM-02	VT47_21655	exonuclease V subunit alpha	VT47_23180	phosphatidylserine decarboxylase
VT47_04560	glycosyltransferase	VT47_07210	hypothetical protein	VT47_10425	hypothetical protein	VT47_08970	LysR family transcriptional	VT47_10470	glucose starvation-inducible protein B	VT47_22245	hypothetical protein	VT47_23645	hypothetical protein
VT47_04565	hypothetical protein	VT47_07225	integrase	VT47_10430	hypothetical protein	VT47_09115	histidine kinase	VT47_10475	Clp protease	VT47_22250	hypothetical protein	VT47_23665	hypothetical protein
VT47_04570	HAD superfamily hydrolase-like protein	VT47_07230	N-methyl-transferase related protein	VT47_10435	hypothetical protein	VT47_09555	hypothetical protein	VT47_10480	prophage PssSM-02	VT47_22310	phage replication protein	VT47_23675	hypothetical protein
VT47_04575	UDP-glucose 6-dehydrogenase	VT47_07235	histidine kinase	VT47_10440	prophage PSSB64-02	VT47_09615	hypothetical protein	VT47_10485	hypothetical protein	VT47_22330	hypothetical protein	VT47_23680	hypothetical protein
VT47_04580	glycosyl transferase	VT47_07295	site-specific recombinase	VT47_10470	glucose starvation-inducible protein B	VT47_09680	LuxR family transcriptional	VT47_10640	Mn2+/Fe2+ transporter	VT47_22380	integrase	VT47_24780	hypothetical protein
VT47_04585	glycosyl transferase	VT47_07300	hypothetical protein	VT47_10475	Clp protease	VT47_09795	hypothetical protein	VT47_10670	hypothetical protein	VT47_22400	AraC family transcriptional regulator	VT47_24830	hypothetical protein
VT47_04590	hemolysin	VT47_07625	dehydrogenase	VT47_10485	hypothetical protein	VT47_09800	hypothetical protein	VT47_10675	hypothetical protein	VT47_22625	hypothetical protein	VT47_24895	hypothetical protein
VT47_04595	hypothetical protein	VT47_07670	hypothetical protein	VT47_10525	NADH:flavin oxidoreductase	VT47_09805	ProQ activator of osmoprotectant	VT47_10805	hypothetical protein	VT47_23050	hypothetical protein	VT47_24900	non-ribosomal peptide synthetase
VT47_04600	peptidase	VT47_07875	hypothetical protein	VT47_10550	amidase	VT47_09810	hypothetical protein	VT47_11070	AraC family transcriptional	VT47_23165	hypothetical protein	VT47_24925	hypothetical protein
VT47_04605	hemolysin secretion protein D	VT47_07955	hypothetical protein	VT47_10555	ABC transporter substrate-binding	VT47_09815	hypothetical protein	VT47_11105	hypothetical protein	VT47_23170	hypothetical protein	VT47_24940	hypothetical protein
VT47_04610	channel protein TolC	VT47_08065	hypothetical protein	VT47_10560	dihydrofolate reductase	VT47_09820	hypothetical protein	VT47_11225	hypothetical protein	VT47_23175	hypothetical protein	VT47_24945	hypothetical protein
VT47_04615	glycosyl transferase family 1	VT47_08340	hypothetical protein	VT47_10565	monooxygenase	VT47_09825	hypothetical protein	VT47_11230	type IV secretion protein Rhs	VT47_23180	phosphatidylserine decarboxylase	VT47_24960	hypothetical protein
VT47_04620	mannose-1-phosphate guanyltransferase	VT47_08395	hypothetical protein	VT47_10665	fimbrial protein	VT47_09830	hypothetical protein	VT47_11235	Major exported protein	VT47_23645	hypothetical protein	VT47_24965	hypothetical protein
VT47_04825	glycerol acyltransferase	VT47_08400	hypothetical protein	VT47_10670	hypothetical protein	VT47_09840	hypothetical protein	VT47_11240	type VI secretion protein ImpA	VT47_23665	hypothetical protein	VT47_25010	hypothetical protein

VT47_04830	LITAF-like zinc ribbon domain containing	VT47_08405	hypothetical protein	VT47_10675	hypothetical protein	VT47_09850	hypothetical protein	VT47_11245	type VI secretion protein	VT47_23675	hypothetical protein	VT47_25015	hypothetical protein
VT47_04835	hypothetical protein	VT47_08435	ArsR family transcriptional regulator	VT47_10805	hypothetical protein	VT47_09860	hypothetical protein	VT47_11255	type VI secretion protein	VT47_23680	hypothetical protein	VT47_25020	hypothetical protein
VT47_04840	hypothetical protein	VT47_08525	ribbon-helix-helix protein domain-containing	VT47_11065	lysine transporter LysE	VT47_09865	hypothetical protein	VT47_11260	type VI secretion protein	VT47_24780	hypothetical protein	VT47_25025	hypothetical protein
VT47_04845	peptidase M48	VT47_08635	hypothetical protein	VT47_11070	AraC family transcriptional regulator	VT47_09870	hypothetical protein	VT47_11265	type VI secretion protein	VT47_24830	hypothetical protein	VT47_25030	hypothetical protein
VT47_04850	membrane protein	VT47_08640	hypothetical protein	VT47_11105	hypothetical protein	VT47_09875	hypothetical protein	VT47_11275	Fis family transcriptional	VT47_24895	hypothetical protein	VT47_25040	hypothetical protein
VT47_04855	16S rRNA methyltransferase	VT47_08645	hypothetical protein	VT47_11220	hypothetical protein	VT47_09880	hypothetical protein	VT47_11280	hypothetical protein	VT47_24900	non-ribosomal peptide synthetase	VT47_25045	beta-lactamase TEM
VT47_04975	hypothetical protein	VT47_08650	hypothetical protein	VT47_11305	acetyltransferase	VT47_10100	nucleotide cyclase	VT47_11285	type VI secretion protein	VT47_24910	hypothetical protein	VT47_25080	hypothetical protein
VT47_04980	hypothetical protein	VT47_08655	hypothetical protein	VT47_11860	peptidylprolyl isomerase	VT47_10410	integrase	VT47_11290	type VI secretion protein	VT47_24925	hypothetical protein	VT47_25085	hypothetical protein
VT47_05040	serine/threonine protein kinase	VT47_08660	type III effector HopAZ1	VT47_11895	hypothetical protein	VT47_10415	hypothetical protein	VT47_11295	membrane protein	VT47_24940	hypothetical protein	VT47_25090	hypothetical protein
VT47_05120	hypothetical protein	VT47_08965	cupin	VT47_12030	hypothetical protein	VT47_10420	prophage PssSM-03	VT47_11300	type VI secretion protein VasK	VT47_24945	hypothetical protein		
VT47_05125	peptidase	VT47_08970	LysR family transcriptional regulator	VT47_12230	fatty acid desaturase	VT47_10430	hypothetical protein	VT47_11345	hypothetical protein	VT47_24960	hypothetical protein		
VT47_05130	membrane protein	VT47_09095	ligand-gated channel	VT47_12235	hypothetical protein	VT47_10470	glucose starvation-inducible protein B	VT47_11895	hypothetical protein	VT47_24965	hypothetical protein		
VT47_05185	YD repeat-containing protein	VT47_09555	hypothetical protein	VT47_12240	hypothetical protein	VT47_10475	Clp protease	VT47_12030	hypothetical protein	VT47_25010	hypothetical protein		
VT47_05315	2'-hydroxyisoflavone reductase	VT47_09785	transposase	VT47_12245	hypothetical protein	VT47_10480	prophage PssSM-02	VT47_12065	ABC transporter substrate-binding	VT47_25015	hypothetical protein		
VT47_05320	HxlR family transcriptional regulator	VT47_09795	hypothetical protein	VT47_12250	metal-dependent hydrolase	VT47_10550	amidase	VT47_12230	fatty acid desaturase	VT47_25020	hypothetical protein		
VT47_05605	hypothetical protein	VT47_09800	hypothetical protein	VT47_12255	fatty acid desaturase	VT47_10665	fimbrial protein	VT47_12235	hypothetical protein	VT47_25025	hypothetical protein		
VT47_05675	hypothetical protein	VT47_09805	ProQ activator of osmoprotectant	VT47_12260	auxin-responsive GH3-related protein	VT47_10670	hypothetical protein	VT47_12240	hypothetical protein	VT47_25030	hypothetical protein		
VT47_05680	hypothetical protein	VT47_09810	hypothetical protein	VT47_12265	FeS-binding protein	VT47_10675	hypothetical protein	VT47_12245	hypothetical protein	VT47_25040	hypothetical protein		
VT47_05785	lipoprotein	VT47_09815	hypothetical protein	VT47_12330	peptide synthetase	VT47_11105	hypothetical protein	VT47_12250	metal-dependent hydrolase	VT47_25045	beta-lactamase TEM		
VT47_05825	type III effector	VT47_09820	hypothetical protein	VT47_12440	transposase	VT47_11220	hypothetical protein	VT47_12255	fatty acid desaturase	VT47_25065	hypothetical protein		
VT47_05835	type III chaperone ShcM	VT47_09825	hypothetical protein	VT47_12520	amino acid adenylation	VT47_11305	acetyltransferase	VT47_12260	auxin-responsive GH3-related protein	VT47_25080	hypothetical protein		
VT47_05840	restriction endonuclease Eco57I	VT47_09830	hypothetical protein	VT47_12525	syringomycin synthetase	VT47_11895	hypothetical protein	VT47_12265	FeS-binding protein	VT47_25085	hypothetical protein		
VT47_05860	ATPase AAA	VT47_09835	serine/threonine protein phosphatase	VT47_12530	peptide synthetase	VT47_12030	hypothetical protein	VT47_12440	transposase	VT47_25090	hypothetical protein		
VT47_05870	membrane protein	VT47_09840	hypothetical protein	VT47_12560	hypothetical protein	VT47_12330	peptide synthetase	VT47_12525	syringomycin synthetase				
VT47_05995	hypothetical protein	VT47_09850	hypothetical protein	VT47_12565	hypothetical protein	VT47_12440	transposase	VT47_12565	hypothetical protein				

VT47_06000	avirulence protein	VT47_09855	hypothetical protein	VT47_12570	non-ribosomal peptide synthetase	VT47_12520	amino acid adenylation	VT47_12595	amino acid adenylation
VT47_06005	type III secretion chaperone CesT	VT47_09860	hypothetical protein	VT47_12575	peptide synthetase	VT47_12525	syringomycin synthetase	VT47_12710	hypothetical protein
VT47_06010	hypothetical protein	VT47_09865	hypothetical protein	VT47_12580	hypothetical protein	VT47_12530	peptide synthetase	VT47_12715	hypothetical protein
VT47_06200	chemotaxis protein CheY	VT47_09870	hypothetical protein	VT47_12585	non-ribosomal peptide synthetase	VT47_12560	hypothetical protein	VT47_12725	hypothetical protein
VT47_06205	dihydropteridine reductase	VT47_09875	hypothetical protein	VT47_12595	amino acid adenylation	VT47_12565	hypothetical protein	VT47_12730	hypothetical protein
VT47_06210	2-nitropropane dioxygenase	VT47_09880	hypothetical protein	VT47_12600	hypothetical protein	VT47_12570	non-ribosomal peptide synthetase	VT47_12735	hypothetical protein
VT47_06215	tautomerase	VT47_10135	hypothetical protein	VT47_12605	acetyltransferase	VT47_12575	peptide synthetase	VT47_12760	hypothetical protein
VT47_06230	hypothetical protein	VT47_10410	integrase	VT47_12615	amino acid adenylation protein	VT47_12580	hypothetical protein	VT47_12765	hypothetical protein
VT47_06530	lipoprotein-related protein, YcsW	VT47_10415	hypothetical protein	VT47_12705	hypothetical protein	VT47_12585	non-ribosomal peptide synthetase	VT47_12775	hypothetical protein
VT47_06555	beta-(1-3)-glucosyl transferase	VT47_10420	prophage PssSM-03	VT47_12710	hypothetical protein	VT47_12595	amino acid adenylation	VT47_12780	hypothetical protein
VT47_06590	zinc-dependent metalloprotease	VT47_10430	hypothetical protein	VT47_12715	hypothetical protein	VT47_12600	hypothetical protein	VT47_12910	beta-glucosidase
VT47_07005	integrase	VT47_10435	hypothetical protein	VT47_12725	hypothetical protein	VT47_12615	amino acid adenylation protein	VT47_12915	UDP-galactopyranose mutase
VT47_07020	hypothetical protein	VT47_10445	prophage PssSM-02	VT47_12730	hypothetical protein	VT47_12700	hypothetical protein	VT47_12920	glycosyl transferase
VT47_07025	Clp protease	VT47_10470	glucose starvation-inducible protein B	VT47_12735	hypothetical protein	VT47_12710	hypothetical protein	VT47_12925	UDP-glucose 4-epimerase
VT47_07030	histidine kinase	VT47_10475	Clp protease	VT47_12750	recombinase	VT47_12715	hypothetical protein	VT47_13240	regulatory protein
VT47_07035	hypothetical protein	VT47_10550	amidase	VT47_12755	integrase	VT47_12725	hypothetical protein	VT47_13335	hypothetical protein
VT47_07040	hypothetical protein	VT47_10665	fimbrial protein	VT47_12760	hypothetical protein	VT47_12730	hypothetical protein	VT47_13365	hypothetical protein
VT47_07045	general stress protein	VT47_10670	hypothetical protein	VT47_12765	hypothetical protein	VT47_12735	hypothetical protein	VT47_13410	endoribonuclease L-PSP
VT47_07050	alcohol dehydrogenase	VT47_10675	hypothetical protein	VT47_12775	hypothetical protein	VT47_12750	recombinase	VT47_13415	monoamine oxidase
VT47_07055	cyclase/dehydrase	VT47_10805	hypothetical protein	VT47_12780	hypothetical protein	VT47_12755	integrase	VT47_13420	ABC transporter substrate-binding
VT47_07060	hypothetical protein	VT47_10810	nuclease	VT47_12965	nucleoside hydrolase	VT47_12760	hypothetical protein	VT47_13430	amino acid ABC transporter permease
VT47_07065	chemotaxis protein	VT47_11030	hypothetical protein	VT47_13100	hypothetical protein	VT47_12765	hypothetical protein	VT47_13435	ligand-gated channel
VT47_07070	hypothetical protein	VT47_11065	lysine transporter LysE	VT47_13105	LysR family transcriptional regulator	VT47_12775	hypothetical protein	VT47_13440	ATPase AAA
VT47_07075	chemotaxis protein	VT47_11070	AraC family transcriptional regulator	VT47_13240	regulatory protein	VT47_12780	hypothetical protein	VT47_13445	dehydrogenase
VT47_07080	lipoprotein	VT47_11105	hypothetical protein	VT47_13260	hypothetical protein	VT47_12965	nucleoside hydrolase	VT47_13450	hydroxypyruvate isomerase

VT47_07090	hypothetical protein	VT47_11110	hypothetical protein	VT47_13335	hypothetical protein	VT47_13100	hypothetical protein	VT47_13470	xanthine dehydrogenase
VT47_07100	ompetence-damaged protein	VT47_11220	hypothetical protein	VT47_13365	hypothetical protein	VT47_13105	LysR family transcriptional	VT47_13480	4-carboxymuconolactone
VT47_07105	transposase	VT47_11225	hypothetical protein	VT47_13410	endoribonuclease L-PSP	VT47_13240	regulatory protein	VT47_13485	NADPH:quinone reductase
VT47_07125	phenazine biosynthesis protein PhzF	VT47_11230	type IV secretion protein Rhs	VT47_13415	monoamine oxidase	VT47_13260	hypothetical protein	VT47_13490	hypothetical protein
VT47_07130	hypothetical protein	VT47_11235	Major exported protein	VT47_13420	ABC transporter substrate-binding	VT47_13335	hypothetical protein	VT47_13495	hypothetical protein
VT47_07135	hypothetical protein	VT47_11240	type VI secretion protein ImpA	VT47_13425	arginine ABC transporter ATP-binding	VT47_13365	hypothetical protein	VT47_14280	hypothetical protein
VT47_07145	hypothetical protein	VT47_11245	type VI secretion protein	VT47_13430	amino acid ABC transporter permease	VT47_13605	amino acid adenylation	VT47_14285	hypothetical protein
VT47_07165	aspartate carbamoyltransferase	VT47_11250	type VI secretion protein	VT47_13435	ligand-gated channel	VT47_14285	hypothetical protein	VT47_14290	hypothetical protein
VT47_07170	MFS transporter	VT47_11255	type VI secretion protein	VT47_13440	ATPase AAA	VT47_14290	hypothetical protein	VT47_14295	hypothetical protein
VT47_07175	methyltransferase type 11	VT47_11260	type VI secretion protein	VT47_13445	dehydrogenase	VT47_14295	hypothetical protein	VT47_14300	hypothetical protein
VT47_07180	glucose starvation-inducible protein B	VT47_11265	type VI secretion protein	VT47_13450	hydroxypyruvate isomerase	VT47_14300	hypothetical protein	VT47_14305	hypothetical protein
VT47_07185	general stress protein	VT47_11270	ATPase AAA	VT47_13460	ferredoxin	VT47_14305	hypothetical protein	VT47_14375	energy transducer TonB
VT47_07195	hypothetical protein	VT47_11275	Fis family transcriptional regulator	VT47_13465	molybdopterin dehydrogenase	VT47_14315	filamentous hemagglutinin	VT47_14620	hypothetical protein
VT47_07205	hypothetical protein	VT47_11280	hypothetical protein	VT47_13470	xanthine dehydrogenase	VT47_14375	energy transducer TonB	VT47_15015	hypothetical protein
VT47_07210	hypothetical protein	VT47_11285	type VI secretion protein	VT47_13475	LysR family transcriptional regulator	VT47_14385	hypothetical protein	VT47_15235	hypothetical protein
VT47_07225	integrase	VT47_11290	type VI secretion protein	VT47_13480	4-carboxymuconolactone	VT47_14540	membrane protein	VT47_15735	dehydrogenase
VT47_07230	N-methyl-transferase related protein	VT47_11295	membrane protein	VT47_13485	NADPH:quinone reductase	VT47_15015	hypothetical protein	VT47_15740	aspartate aminotransferase
VT47_07235	histidine kinase	VT47_11300	type VI secretion protein VasK	VT47_13490	hypothetical protein	VT47_15235	hypothetical protein	VT47_15745	DNA mismatch repair protein MutT
VT47_07295	site-specific recombinase	VT47_11305	acetyltransferase	VT47_13495	hypothetical protein	VT47_15910	hypothetical protein	VT47_15750	aminotransferase
VT47_07300	hypothetical protein	VT47_11315	alcohol dehydrogenase	VT47_13605	amino acid adenylation	VT47_15920	hypothetical protein	VT47_15755	FAD-dependent oxidoreductase
VT47_07305	hypothetical protein	VT47_11320	transcriptional regulator	VT47_14165	oxidoreductase	VT47_15930	hypothetical protein	VT47_15760	phosphoglycolate phosphatase
VT47_07310	fimbrial protein	VT47_11345	hypothetical protein	VT47_14170	LysR family transcriptional regulator	VT47_16075	hypothetical protein	VT47_15765	dihydrorhizobitoxine desaturase
VT47_07415	hypothetical protein	VT47_11860	peptidylprolyl isomerase	VT47_14280	hypothetical protein	VT47_16285	hypothetical protein	VT47_15770	5'-methylthioadenosine phosphorylase
VT47_07420	WbqC-like family protein	VT47_11895	hypothetical protein	VT47_14285	hypothetical protein	VT47_16560	hypothetical protein	VT47_15775	GDP-mannose pyrophosphatase
VT47_07610	CoA-transferase	VT47_12025	hypothetical protein	VT47_14290	hypothetical protein	VT47_16565	membrane protein	VT47_15780	ABC transporter permease

VT47_07625	dehydrogenase	VT47_12030	hypothetical protein	VT47_14295	hypothetical protein	VT47_17675	phosphoketolase	VT47_15785	MFS transporter
VT47_07870	threonine dehydratase	VT47_12230	fatty acid desaturase	VT47_14300	hypothetical protein	VT47_17680	propionate kinase	VT47_15790	haloacid dehalogenase
VT47_07920	diguanylate cyclase	VT47_12235	hypothetical protein	VT47_14305	hypothetical protein	VT47_17685	universal stress protein	VT47_15900	alanine acetyltransferase
VT47_07955	hypothetical protein	VT47_12240	hypothetical protein	VT47_14315	filamentous hemagglutinin	VT47_17690	Crp/Fnr family transcriptional	VT47_15910	hypothetical protein
VT47_08010	LuxR family transcriptional regulator	VT47_12245	hypothetical protein	VT47_14375	energy transducer TonB	VT47_17695	ion transporter	VT47_15920	hypothetical protein
VT47_08015	fatty acid desaturase	VT47_12250	metal-dependent hydrolase	VT47_14385	hypothetical protein	VT47_17700	hypothetical protein	VT47_15930	hypothetical protein
VT47_08020	thioester reductase	VT47_12255	fatty acid desaturase	VT47_14690	beta-glucosidase	VT47_17705	GNAT family acetyltransferase	VT47_16075	hypothetical protein
VT47_08025	amino acid adenylation	VT47_12260	auxin-responsive GH3-related protein	VT47_15015	hypothetical protein	VT47_17720	hypothetical protein	VT47_17675	phosphoketolase
VT47_08030	MFS transporter	VT47_12265	FeS-binding protein	VT47_15235	hypothetical protein	VT47_17845	CopG family transcriptional	VT47_17680	propionate kinase
VT47_08340	hypothetical protein	VT47_12330	peptide synthetase	VT47_15555	TetR family transcriptional regulator	VT47_17850	periplasmic or secreted lipoprotein	VT47_17685	universal stress protein
VT47_08345	hypothetical protein	VT47_12435	lysine exporter protein LysE/YggA	VT47_15900	alanine acetyltransferase	VT47_17915	hypothetical protein	VT47_17695	ion transporter
VT47_08395	hypothetical protein	VT47_12440	transposase	VT47_15910	hypothetical protein	VT47_18080	hypothetical protein	VT47_17700	hypothetical protein
VT47_08400	hypothetical protein	VT47_12455	transcriptional regulator	VT47_15920	hypothetical protein	VT47_18115	mechanosensitive ion channel protein MscS	VT47_17705	GNAT family acetyltransferase
VT47_08405	hypothetical protein	VT47_12460	MFS transporter permease	VT47_15930	hypothetical protein	VT47_18120	hypothetical protein	VT47_17755	restriction endonuclease
VT47_08415	histidine kinase	VT47_12465	3-phosphoglycerate dehydrogenase	VT47_16075	hypothetical protein	VT47_18125	hypothetical protein	VT47_17760	ATPase
VT47_08435	ArsR family transcriptional regulator	VT47_12470	hypothetical protein	VT47_16560	hypothetical protein	VT47_18135	hypothetical protein	VT47_17845	CopG family transcriptional
VT47_08470	oxidoreductase	VT47_12475	alcohol dehydrogenase	VT47_16565	membrane protein	VT47_18140	hypothetical protein	VT47_17850	periplasmic or secreted lipoprotein
VT47_08480	thioester reductase	VT47_12505	hypothetical protein	VT47_17090	biotin transporter BioY	VT47_18145	hypothetical protein	VT47_17915	hypothetical protein
VT47_08485	thioester reductase	VT47_12525	syringomycin synthetase	VT47_17675	phosphoketolase	VT47_18150	hypothetical protein	VT47_18080	hypothetical protein
VT47_08490	thioester reductase	VT47_12530	peptide synthetase	VT47_17680	propionate kinase	VT47_18160	hypothetical protein	VT47_18120	hypothetical protein
VT47_08495	taurine dioxygenase	VT47_12560	hypothetical protein	VT47_17685	universal stress protein	VT47_18200	hypothetical protein	VT47_18125	hypothetical protein
VT47_08500	acetyltransferase	VT47_12565	hypothetical protein	VT47_17690	Crp/Fnr family transcriptional regulator	VT47_18235	leucine-rich repeat domain protein	VT47_18135	hypothetical protein
VT47_08635	hypothetical protein	VT47_12570	non-ribosomal peptide synthetase	VT47_17695	ion transporter	VT47_18455	zinc chelation protein SecC	VT47_18140	hypothetical protein
VT47_08640	hypothetical protein	VT47_12575	peptide synthetase	VT47_17700	hypothetical protein	VT47_18685	5- methyltetrahydroptero	VT47_18145	hypothetical protein
VT47_08645	hypothetical protein	VT47_12580	hypothetical protein	VT47_17705	GNAT family acetyltransferase	VT47_18720	hypothetical protein	VT47_18150	hypothetical protein

VT47_08650	hypothetical protein	VT47_12585	non-ribosomal peptide synthetase	VT47_17715	hypothetical protein	VT47_18725	hypothetical protein	VT47_18155	hypothetical protein
VT47_08655	hypothetical protein	VT47_12595	amino acid adenylation	VT47_17720	hypothetical protein	VT47_18960	hypothetical protein	VT47_18160	hypothetical protein
VT47_08660	type III effector HopAZ1	VT47_12600	hypothetical protein	VT47_17915	hypothetical protein	VT47_19025	hypothetical protein	VT47_18165	nuclease
VT47_08840	LuxR family transcriptional regulator	VT47_12605	acetyltransferase	VT47_18080	hypothetical protein	VT47_19270	toxin	VT47_18170	lipoprotein
VT47_08965	cupin	VT47_12610	gluconate transporter	VT47_18120	hypothetical protein	VT47_19295	hypothetical protein	VT47_18175	hypothetical protein
VT47_08970	LysR family transcriptional regulator	VT47_12615	amino acid adenylation protein	VT47_18125	hypothetical protein	VT47_19500	hypothetical protein	VT47_18200	hypothetical protein
VT47_09095	ligand-gated channel	VT47_12665	GntR family transcriptional regulator	VT47_18135	hypothetical protein	VT47_20105	fimbrial protein	VT47_18250	antitoxin
VT47_09225	pyoverdine sidechain peptide synthetase	VT47_12670	serine kinase	VT47_18145	hypothetical protein	VT47_20110	bacteriocin	VT47_18455	zinc chelation protein SecC
VT47_09235	peptide synthetase	VT47_12675	4-aminobutyrate aminotransferase	VT47_18150	hypothetical protein	VT47_20120	prophage PssSM-03	VT47_18585	(2Fe-2S)-binding protein
VT47_09520	acetyltransferase domain protein	VT47_12680	dehalogenase	VT47_18155	hypothetical protein	VT47_20130	hypothetical protein	VT47_18960	hypothetical protein
VT47_09555	hypothetical protein	VT47_12695	Type III secretion system effector HopBA1	VT47_18160	hypothetical protein	VT47_20140	hypothetical protein	VT47_19005	helicase
VT47_09615	hypothetical protein	VT47_12705	hypothetical protein	VT47_18165	nuclease	VT47_20150	hypothetical protein	VT47_19025	hypothetical protein
VT47_09625	hypothetical protein	VT47_12710	hypothetical protein	VT47_18170	lipoprotein	VT47_20160	hypothetical protein	VT47_20110	bacteriocin
VT47_09630	fimbrial protein	VT47_12715	hypothetical protein	VT47_18200	hypothetical protein	VT47_20170	hypothetical protein	VT47_20130	hypothetical protein
VT47_09635	ferrous iron transporter B	VT47_12725	hypothetical protein	VT47_18455	zinc chelation protein SecC	VT47_20175	hypothetical protein	VT47_20140	hypothetical protein
VT47_09640	pilus assembly protein	VT47_12730	hypothetical protein	VT47_18545	endonuclease III	VT47_20180	hypothetical protein	VT47_20160	hypothetical protein
VT47_09645	pilus assembly protein	VT47_12735	hypothetical protein	VT47_18685	5-methyltetrahydropteroyl [illegible]	VT47_20190	Appr-1-p processing protein	VT47_20170	hypothetical protein
VT47_09660	hypothetical protein	VT47_12750	recombinase	VT47_18720	hypothetical protein	VT47_20200	diguanylate cyclase	VT47_20175	hypothetical protein
VT47_09665	imidazole glycerol phosphate synthase	VT47_12755	integrase	VT47_18725	hypothetical protein	VT47_20245	hypothetical protein	VT47_20190	Appr-1-p processing protein
VT47_09670	lipoprotein	VT47_12760	hypothetical protein	VT47_18960	hypothetical protein	VT47_20535	LuxR family transcriptional [illegible]	VT47_20375	toxin
VT47_09780	transposase	VT47_12775	hypothetical protein	VT47_19025	hypothetical protein	VT47_20585	hypothetical protein	VT47_20380	toxin
VT47_09785	transposase	VT47_12780	hypothetical protein	VT47_19295	hypothetical protein	VT47_20690	hypothetical protein	VT47_20585	hypothetical protein
VT47_09795	hypothetical protein	VT47_12965	nucleoside hydrolase	VT47_19485	hypothetical protein	VT47_20695	hypothetical protein	VT47_20680	hypothetical protein
VT47_09800	hypothetical protein	VT47_13030	endoribonuclease L-PSP	VT47_20140	hypothetical protein	VT47_20700	hypothetical protein	VT47_20690	hypothetical protein
VT47_09805	ProQ activator of osmoprotectant	VT47_13225	hypothetical protein	VT47_20160	hypothetical protein	VT47_20720	hypothetical protein	VT47_20695	hypothetical protein

VT47_09810	hypothetical protein	VT47_13240	regulatory protein	VT47_20170	hypothetical protein	VT47_20780	hypothetical protein	VT47_20700	hypothetical protein
VT47_09815	hypothetical protein	VT47_13250	hypothetical protein	VT47_20175	hypothetical protein	VT47_21330	hypothetical protein	VT47_20720	hypothetical protein
VT47_09820	hypothetical protein	VT47_13255	prophage PssSM-03	VT47_20180	hypothetical protein	VT47_21470	hypothetical protein	VT47_20780	hypothetical protein
VT47_09825	hypothetical protein	VT47_13260	hypothetical protein	VT47_20190	Appr-1-p processing protein	VT47_21530	hypothetical protein	VT47_21330	hypothetical protein
VT47_09830	hypothetical protein	VT47_13265	chemotaxis protein	VT47_20245	hypothetical protein	VT47_21535	hypothetical protein	VT47_21530	hypothetical protein
VT47_09835	serine/threonine protein phosphatase	VT47_13325	acyl-CoA dehydrogenase	VT47_20535	LuxR family transcriptional regulator	VT47_21540	short-chain dehydrogenase	VT47_21535	hypothetical protein
VT47_09840	hypothetical protein	VT47_13330	acyl-CoA dehydrogenase	VT47_20600	MFS transporter	VT47_21625	hypothetical protein	VT47_21540	short-chain dehydrogenase
VT47_09850	hypothetical protein	VT47_13335	hypothetical protein	VT47_20690	hypothetical protein	VT47_21630	hypothetical protein	VT47_21625	hypothetical protein
VT47_09855	hypothetical protein	VT47_13365	hypothetical protein	VT47_20695	hypothetical protein	VT47_21635	thiamine biosynthesis protein ThiF	VT47_21630	hypothetical protein
VT47_09860	hypothetical protein	VT47_13380	hypothetical protein	VT47_20700	hypothetical protein	VT47_21640	hypothetical protein	VT47_21635	thiamine biosynthesis protein ThiF
VT47_09865	hypothetical protein	VT47_13440	ATPase AAA	VT47_20720	hypothetical protein	VT47_21645	patatin	VT47_21640	hypothetical protein
VT47_09870	hypothetical protein	VT47_13445	dehydrogenase	VT47_20780	hypothetical protein	VT47_21650	hypothetical protein	VT47_21645	patatin
VT47_09875	hypothetical protein	VT47_13450	hydroxypyruvate isomerase	VT47_21330	hypothetical protein	VT47_21655	exonuclease V subunit alpha	VT47_21650	hypothetical protein
VT47_09880	hypothetical protein	VT47_13460	ferredoxin	VT47_21470	hypothetical protein	VT47_22135	hypothetical protein	VT47_21655	exonuclease V subunit alpha
VT47_10075	sucrase	VT47_13465	molybdopterin dehydrogenase	VT47_21530	hypothetical protein	VT47_22245	hypothetical protein	VT47_22015	tail fiber protein
VT47_10410	integrase	VT47_13470	xanthine dehydrogenase	VT47_21535	hypothetical protein	VT47_22250	hypothetical protein	VT47_22090	bacteriocin
VT47_10415	hypothetical protein	VT47_13475	LysR family transcriptional regulator	VT47_21540	short-chain dehydrogenase	VT47_22305	plasmid-related protein	VT47_22135	hypothetical protein
VT47_10430	hypothetical protein	VT47_13480	4-carboxymuconolactone decarboxylase	VT47_21625	hypothetical protein	VT47_22310	phage replication protein	VT47_22245	hypothetical protein
VT47_10435	hypothetical protein	VT47_13485	NADPH:quinone reductase	VT47_21630	hypothetical protein	VT47_22330	hypothetical protein	VT47_22250	hypothetical protein
VT47_10440	prophage PSSB64-02	VT47_13490	hypothetical protein	VT47_21635	thiamine biosynthesis protein ThiF	VT47_22355	hypothetical protein	VT47_22300	integrase
VT47_10445	prophage PssSM-02	VT47_13495	hypothetical protein	VT47_21640	hypothetical protein	VT47_22380	integrase	VT47_22305	plasmid-related protein
VT47_10465	prophage PssSM-03, GDSL-like [illegible]	VT47_13600	hypothetical protein	VT47_21645	patatin	VT47_22395	lysine transporter LysE	VT47_22310	phage replication protein
VT47_10470	glucose starvation-inducible protein B	VT47_13605	amino acid adenylation	VT47_21650	hypothetical protein	VT47_22400	AraC family transcriptional [illegible]	VT47_22315	conjugal transfer protein TrbJ
VT47_10475	Clp protease	VT47_14280	hypothetical protein	VT47_21655	exonuclease V subunit alpha	VT47_22625	hypothetical protein	VT47_22320	conjugal transfer protein TrbJ
VT47_10485	hypothetical protein	VT47_14285	hypothetical protein	VT47_22010	tail fiber assembly protein	VT47_22630	transposase	VT47_22325	conjugal transfer protein

VT47_10530	RND transporter	VT47_14290	hypothetical protein	VT47_22015	tail fiber protein	VT47_23050	hypothetical protein	VT47_22330	hypothetical protein
VT47_10550	amidase	VT47_14295	hypothetical protein	VT47_22090	bacteriocin	VT47_23080	cytochrome C	VT47_22335	RelE/ParE family plasmid stabilization
VT47_10555	ABC transporter substrate-binding	VT47_14300	hypothetical protein	VT47_22135	hypothetical protein	VT47_23105	porin	VT47_22340	prevent-host-death protein
VT47_10560	dihydrofolate reductase	VT47_14305	hypothetical protein	VT47_22245	hypothetical protein	VT47_23160	cystathionine beta-synthase	VT47_22355	hypothetical protein
VT47_10565	monooxygenase	VT47_14315	filamentous hemagglutinin	VT47_22250	hypothetical protein	VT47_23165	hypothetical protein	VT47_22375	hypothetical protein
VT47_10640	Mn2+/Fe2+ transporter	VT47_14320	ShlB family hemolysin secretion/activation	VT47_22310	phage replication protein	VT47_23170	hypothetical protein	VT47_22380	integrase
VT47_10645	hydrolase	VT47_14330	acriflavin resistance protein	VT47_22315	conjugal transfer protein TrbJ	VT47_23175	hypothetical protein	VT47_22420	toxin/anti-toxin system, HipA-like toxin module
VT47_10665	fimbrial protein	VT47_14375	energy transducer TonB	VT47_22320	conjugal transfer protein TrbJ	VT47_23180	phosphatidylserine decarboxylase	VT47_22425	XRE family transcriptional
VT47_10670	hypothetical protein	VT47_14380	diguanylate cyclase	VT47_22325	conjugal transfer protein	VT47_23445	hypothetical protein	VT47_22625	hypothetical protein
VT47_10675	hypothetical protein	VT47_14385	hypothetical protein	VT47_22330	hypothetical protein	VT47_23590	Fic family protein	VT47_23050	hypothetical protein
VT47_10690	chemotaxis protein	VT47_14980	AraC family transcriptional regulator	VT47_22335	RelE/ParE family plasmid stabilization	VT47_23645	hypothetical protein	VT47_23165	hypothetical protein
VT47_10695	rhizopine-binding protein	VT47_14985	alpha/beta hydrolase	VT47_22340	prevent-host-death protein	VT47_23660	hypothetical protein	VT47_23170	hypothetical protein
VT47_10705	histidine kinase	VT47_15015	hypothetical protein	VT47_22380	integrase	VT47_23665	hypothetical protein	VT47_23175	hypothetical protein
VT47_10710	chemotaxis protein	VT47_15135	antibiotic biosynthesis monooxygenase	VT47_22395	lysine transporter LysE	VT47_23675	hypothetical protein	VT47_23180	phosphatidylserine decarboxylase
VT47_10805	hypothetical protein	VT47_15235	hypothetical protein	VT47_22400	AraC family transcriptional regulator	VT47_23680	hypothetical protein	VT47_23445	hypothetical protein
VT47_11005	membrane protein	VT47_15655	acetyltransferase	VT47_22625	hypothetical protein	VT47_23690	phosphoglycerate kinase	VT47_23590	Fic family protein
VT47_11010	membrane protein	VT47_15735	dehydrogenase	VT47_23050	hypothetical protein	VT47_23695	hypothetical protein	VT47_23645	hypothetical protein
VT47_11015	short-chain dehydrogenase	VT47_15740	aspartate aminotransferase	VT47_23160	cystathionine beta-synthase	VT47_23700	prophage PssSM-03	VT47_23660	hypothetical protein
VT47_11020	FAD-linked oxidase	VT47_15745	DNA mismatch repair protein MutT	VT47_23165	hypothetical protein	VT47_23825	hypothetical protein	VT47_23665	hypothetical protein
VT47_11025	hypothetical protein	VT47_15750	aminotransferase	VT47_23170	hypothetical protein	VT47_23910	PAAR domain-containing protein	VT47_23675	hypothetical protein
VT47_11065	lysine transporter LysE	VT47_15755	FAD-dependent oxidoreductase	VT47_23175	hypothetical protein	VT47_23915	phospholipase	VT47_23680	hypothetical protein
VT47_11070	AraC family transcriptional regulator	VT47_15760	phosphoglycolate phosphatase	VT47_23180	phosphatidylserine decarboxylase	VT47_23920	Sel1 repeat-containing protein	VT47_23685	hypothetical protein
VT47_11105	hypothetical protein	VT47_15765	dihydrorhizobitoxine desaturase	VT47_23445	hypothetical protein	VT47_23925	Sel1 repeat-containing protein	VT47_23785	peptidoglycan-binding protein LysM
VT47_11220	hypothetical protein	VT47_15770	5'-methylthioadenosine phosphorylase	VT47_23645	hypothetical protein	VT47_23930	hypothetical protein	VT47_23825	hypothetical protein
VT47_11280	hypothetical protein	VT47_15775	GDP-mannose pyrophosphatase	VT47_23660	hypothetical protein	VT47_23935	type IV secretion protein Rhs	VT47_23955	hypothetical protein

VT47_11305	acetyltransferase	VT47_15780	ABC transporter permease	VT47_23665	hypothetical protein	VT47_23940	hypothetical protein	VT47_23960	hypothetical protein
VT47_11335	hypothetical protein	VT47_15785	MFS transporter	VT47_23675	hypothetical protein	VT47_23955	hypothetical protein	VT47_23970	hypothetical protein
VT47_11445	hypothetical protein	VT47_15790	haloacid dehalogenase	VT47_23680	hypothetical protein	VT47_23970	hypothetical protein	VT47_23985	membrane protein
VT47_11455	DNA methylase	VT47_15900	alanine acetyltransferase	VT47_23690	phosphoglycerate kinase	VT47_23980	hypothetical protein	VT47_24345	hypothetical protein
VT47_11460	hypothetical protein	VT47_15910	hypothetical protein	VT47_23695	hypothetical protein	VT47_24005	type IV secretion protein Rhs	VT47_24515	membrane protein
VT47_11475	hypothetical protein	VT47_15920	hypothetical protein	VT47_23700	prophage PssSM-03	VT47_24590	NADPH-dependent FMN reductase	VT47_24580	dTDP-glucose 4,6-dehydratase
VT47_11605	toxin	VT47_15930	hypothetical protein	VT47_23710	GNAT family acetyltransferase	VT47_24595	ArsC family transcriptional	VT47_24585	PEP phosphonomutase
VT47_11860	peptidylprolyl isomerase	VT47_16075	hypothetical protein	VT47_23825	hypothetical protein	VT47_24600	ArsR family transcriptional	VT47_24590	NADPH-dependent FMN reductase
VT47_11895	hypothetical protein	VT47_16080	hypothetical protein	VT47_23910	PAAR domain-containing protein	VT47_24605	chemotaxis protein	VT47_24595	ArsC family transcriptional
VT47_12000	autotransporter	VT47_16145	cytochrome C oxidase subunit II	VT47_23915	phospholipase	VT47_24610	inorganic pyrophosphatase	VT47_24600	ArsR family transcriptional
VT47_12030	hypothetical protein	VT47_16285	hypothetical protein	VT47_23920	Sel1 repeat-containing protein	VT47_24615	hypothetical protein	VT47_24615	hypothetical protein
VT47_12225	metallophosphatase	VT47_16430	hypothetical protein	VT47_23925	Sel1 repeat-containing protein	VT47_24620	camphor resistance protein CrcB	VT47_24620	camphor resistance protein CrcB
VT47_12230	fatty acid desaturase	VT47_16560	hypothetical protein	VT47_23930	hypothetical protein	VT47_24625	chromate transporter	VT47_24625	chromate transporter
VT47_12235	hypothetical protein	VT47_16565	membrane protein	VT47_23935	type IV secretion protein Rhs	VT47_24755	type I restriction-modification system,	VT47_24720	membrane protein insertion efficiency
VT47_12240	hypothetical protein	VT47_16655	lysine transporter LysE	VT47_23940	hypothetical protein	VT47_24765	hypothetical protein	VT47_24755	type I restriction-modification system, M
VT47_12245	hypothetical protein	VT47_16660	YqcI/YcgG family protein	VT47_23950	hypothetical protein	VT47_24770	deoxyribonuclease HsdR	VT47_24765	hypothetical protein
VT47_12250	metal-dependent hydrolase	VT47_16665	LysR family transcriptional regulator	VT47_23955	hypothetical protein	VT47_24775	metal-dependent hydrolase	VT47_24770	deoxyribonuclease HsdR
VT47_12255	fatty acid desaturase	VT47_16720	AraC family transcriptional regulator	VT47_23960	hypothetical protein	VT47_24780	hypothetical protein	VT47_24775	metal-dependent hydrolase
VT47_12260	auxin-responsive GH3-related protein	VT47_16795	hypothetical protein	VT47_23965	hypothetical protein	VT47_24785	transposase	VT47_24780	hypothetical protein
VT47_12265	FeS-binding protein	VT47_17250	peroxiredoxin	VT47_23970	hypothetical protein	VT47_24790	transposase	VT47_24795	hypothetical protein
VT47_12325	peptide synthetase	VT47_17440	hypothetical protein	VT47_23980	hypothetical protein	VT47_24795	hypothetical protein	VT47_24800	haloacid dehalogenase
VT47_12330	peptide synthetase	VT47_17675	phosphoketolase	VT47_23995	hypothetical protein	VT47_24800	haloacid dehalogenase	VT47_24805	hypothetical protein
VT47_12335	peptide synthetase	VT47_17680	propionate kinase	VT47_24005	type IV secretion protein Rhs	VT47_24805	hypothetical protein	VT47_24810	hypothetical protein
VT47_12380	MFS transporter	VT47_17685	universal stress protein	VT47_24720	membrane protein insertion efficiency	VT47_24810	hypothetical protein	VT47_24815	transposase
VT47_12435	lysine exporter protein LysE/YggA	VT47_17690	Crp/Fnr family transcriptional regulator	VT47_24755	type I restriction-modification system, M	VT47_24815	transposase	VT47_24820	transposase

VT47_12440	transposase	VT47_17695	ion transporter	VT47_24765	hypothetical protein	VT47_24820	transposase	VT47_24825	hypothetical protein
VT47_12445	DNA helicase	VT47_17700	hypothetical protein	VT47_24770	deoxyribonuclease HsdR	VT47_24825	hypothetical protein	VT47_24830	hypothetical protein
VT47_12520	amino acid adenylation	VT47_17705	GNAT family acetyltransferase	VT47_24775	metal-dependent hydrolase	VT47_24830	hypothetical protein	VT47_24835	serine/threonine protein phosphatase
VT47_12525	syringomycin synthetase	VT47_17715	hypothetical protein	VT47_24780	hypothetical protein	VT47_24835	serine/threonine protein phosphatase	VT47_24840	plasmid stablization protein ParB
VT47_12530	peptide synthetase	VT47_17720	hypothetical protein	VT47_24785	transposase	VT47_24840	plasmid stablization protein ParB	VT47_24845	plasmid stablization protein ParB
VT47_12565	hypothetical protein	VT47_17730	cytochrome B561	VT47_24790	transposase	VT47_24845	plasmid stablization protein ParB	VT47_24850	serine recombinase
VT47_12570	non-ribosomal peptide synthetase	VT47_17735	TetR family transcriptional regulator	VT47_24795	hypothetical protein	VT47_24850	serine recombinase	VT47_24885	non-ribosomal peptide synthetase
VT47_12585	non-ribosomal peptide synthetase	VT47_17740	multidrug ABC transporter	VT47_24800	haloacid dehalogenase	VT47_24885	non-ribosomal peptide synthetase	VT47_24890	peptide synthetase
VT47_12595	amino acid adenylation	VT47_17745	hypothetical protein	VT47_24805	hypothetical protein	VT47_24890	peptide synthetase	VT47_24895	hypothetical protein
VT47_12600	hypothetical protein	VT47_17750	transcriptional regulator	VT47_24810	hypothetical protein	VT47_24895	hypothetical protein	VT47_24900	non-ribosomal peptide synthetase
VT47_12605	acetyltransferase	VT47_17790	topoisomerase	VT47_24815	transposase	VT47_24900	non-ribosomal peptide synthetase	VT47_24915	hypothetical protein
VT47_12615	amino acid adenylation protein	VT47_17845	CopG family transcriptional regulator	VT47_24820	transposase	VT47_24910	hypothetical protein	VT47_24925	hypothetical protein
VT47_12695	Type III secretion system effector [illegible]	VT47_17850	periplasmic or secreted lipoprotein	VT47_24825	hypothetical protein	VT47_24915	hypothetical protein	VT47_24930	hypothetical protein
VT47_12700	hypothetical protein	VT47_17915	hypothetical protein	VT47_24830	hypothetical protein	VT47_24920	non-ribosomal peptide synthetase	VT47_24940	hypothetical protein
VT47_12705	hypothetical protein	VT47_18050	hypothetical protein	VT47_24835	serine/threonine protein phosphatase	VT47_24925	hypothetical protein	VT47_24945	hypothetical protein
VT47_12710	hypothetical protein	VT47_18080	hypothetical protein	VT47_24840	plasmid stablization protein ParB	VT47_24930	hypothetical protein	VT47_24960	hypothetical protein
VT47_12715	hypothetical protein	VT47_18120	hypothetical protein	VT47_24845	plasmid stablization protein ParB	VT47_24935	filamentous hemagglutinin	VT47_24965	hypothetical protein
VT47_12725	hypothetical protein	VT47_18125	hypothetical protein	VT47_24850	serine recombinase	VT47_24940	hypothetical protein	VT47_24970	cytochrome B561
VT47_12730	hypothetical protein	VT47_18135	hypothetical protein	VT47_24885	non-ribosomal peptide synthetase	VT47_24945	hypothetical protein	VT47_24975	catalase
VT47_12735	hypothetical protein	VT47_18140	hypothetical protein	VT47_24890	peptide synthetase	VT47_24960	hypothetical protein	VT47_24985	transcriptional regulator
VT47_12750	recombinase	VT47_18145	hypothetical protein	VT47_24895	hypothetical protein	VT47_24965	hypothetical protein	VT47_25010	hypothetical protein
VT47_12755	integrase	VT47_18150	hypothetical protein	VT47_24900	non-ribosomal peptide synthetase	VT47_25010	hypothetical protein	VT47_25015	hypothetical protein
VT47_12760	hypothetical protein	VT47_18155	hypothetical protein	VT47_24910	hypothetical protein	VT47_25015	hypothetical protein	VT47_25020	hypothetical protein
VT47_12765	hypothetical protein	VT47_18160	hypothetical protein	VT47_24915	hypothetical protein	VT47_25020	hypothetical protein	VT47_25025	hypothetical protein
VT47_12775	hypothetical protein	VT47_18165	nuclease	VT47_24920	non-ribosomal peptide synthetase	VT47_25025	hypothetical protein	VT47_25030	hypothetical protein

VT47_12780	hypothetical protein	VT47_18170	lipoprotein	VT47_24925	hypothetical protein	VT47_25030	hypothetical protein	VT47_25040	hypothetical protein
VT47_12845	membrane protein	VT47_18200	hypothetical protein	VT47_24930	hypothetical protein	VT47_25035	type IV secretion protein Rhs	VT47_25045	beta-lactamase TEM
VT47_12850	hypothetical protein	VT47_18385	transporter	VT47_24935	filamentous hemagglutinin	VT47_25040	hypothetical protein	VT47_25080	hypothetical protein
VT47_12910	beta-glucosidase	VT47_18390	amidinotransferase	VT47_24940	hypothetical protein	VT47_25045	beta-lactamase TEM	VT47_25085	hypothetical protein
VT47_12915	UDP-galactopyranose mutase	VT47_18395	biotin carboxylase	VT47_24945	hypothetical protein	VT47_25060	hypothetical protein	VT47_25090	hypothetical protein
VT47_12920	glycosyl transferase	VT47_18400	mangotoxin biosynthesis protein MboC	VT47_24960	hypothetical protein	VT47_25065	hypothetical protein		
VT47_12925	UDP-glucose 4-epimerase	VT47_18405	oxidoreductase	VT47_24965	hypothetical protein	VT47_25070	YD repeat-containing protein		
VT47_12965	nucleoside hydrolase	VT47_18410	hypothetical protein	VT47_25010	hypothetical protein	VT47_25075	filamentous hemagglutinin		
VT47_13040	acetyl-CoA acetyltransferase	VT47_18460	glutathione S-transferase	VT47_25015	hypothetical protein	VT47_25080	hypothetical protein		
VT47_13225	hypothetical protein	VT47_18960	hypothetical protein	VT47_25020	hypothetical protein	VT47_25085	hypothetical protein		
VT47_13230	cytochrome C	VT47_19025	hypothetical protein	VT47_25025	hypothetical protein	VT47_25090	hypothetical protein		
VT47_13240	regulatory protein	VT47_19295	hypothetical protein	VT47_25030	hypothetical protein	VT47_25095	transposase		
VT47_13250	hypothetical protein	VT47_19500	hypothetical protein	VT47_25035	type IV secretion protein Rhs	VT47_25105	hypothetical protein		
VT47_13260	hypothetical protein	VT47_20105	fimbrial protein	VT47_25040	hypothetical protein	VT47_25110	hypothetical protein		
VT47_13265	chemotaxis protein	VT47_20110	bacteriocin	VT47_25045	beta-lactamase TEM				
VT47_13280	chitin-binding protein	VT47_20115	prophage PssSM-03	VT47_25060	hypothetical protein				
VT47_13305	hypothetical protein	VT47_20130	hypothetical protein	VT47_25065	hypothetical protein				
VT47_13335	hypothetical protein	VT47_20140	hypothetical protein	VT47_25070	YD repeat-containing protein				
VT47_13365	hypothetical protein	VT47_20150	hypothetical protein	VT47_25075	filamentous hemagglutinin				
VT47_13410	endoribonuclease L-PSP	VT47_20160	hypothetical protein	VT47_25080	hypothetical protein				
VT47_13415	monoamine oxidase	VT47_20170	hypothetical protein	VT47_25085	hypothetical protein				
VT47_13420	ABC transporter substrate-binding [illegible]	VT47_20175	hypothetical protein	VT47_25090	hypothetical protein				
VT47_13425	arginine ABC transporter ATP-[illegible]	VT47_20180	hypothetical protein	VT47_25095	transposase				
VT47_13430	amino acid ABC transporter permease	VT47_20190	Appr-1-p processing protein	VT47_25105	hypothetical protein				
VT47_13435	ligand-gated channel	VT47_20245	hypothetical protein	VT47_25110	hypothetical protein				

VT47_13440	ATPase AAA	VT47_20265	hypothetical protein
VT47_13445	dehydrogenase	VT47_20375	toxin
VT47_13450	hydroxypyruvate isomerase	VT47_20380	toxin
VT47_13460	ferredoxin	VT47_20585	hypothetical protein
VT47_13465	molybdopterin dehydrogenase	VT47_20595	BRO N-terminal domain-containing [illegible]
VT47_13470	xanthine dehydrogenase	VT47_20680	hypothetical protein
VT47_13475	LysR family transcriptional regulator	VT47_20690	hypothetical protein
VT47_13480	4-carboxymuconolactone [illegible]	VT47_20695	hypothetical protein
VT47_13485	NADPH:quinone reductase	VT47_20700	hypothetical protein
VT47_13490	hypothetical protein	VT47_20720	hypothetical protein
VT47_13600	hypothetical protein	VT47_20780	hypothetical protein
VT47_13605	amino acid adenylation	VT47_21285	hypothetical protein
VT47_13610	GntR family transcriptional regulator	VT47_21330	hypothetical protein
VT47_13615	ADP-ribosylglycohydrolase	VT47_21470	hypothetical protein
VT47_13620	allantoin permease	VT47_21475	transposase
VT47_13625	sugar kinase	VT47_21625	hypothetical protein
VT47_13630	toxin HipA	VT47_21630	hypothetical protein
VT47_13635	XRE family transcriptional regulator	VT47_21635	thiamine biosynthesis protein ThiF
VT47_13640	MFS transporter	VT47_21640	hypothetical protein
VT47_13645	TetR family transcriptional regulator	VT47_21645	patatin
VT47_13680	LysR family transcriptional regulator	VT47_21650	hypothetical protein
VT47_14005	cobalt transporter	VT47_21655	exonuclease V subunit alpha
VT47_14150	XRE family transcriptional regulator	VT47_22010	tail fiber assembly protein
VT47_14155	LysR family transcriptional regulator	VT47_22015	tail fiber protein
VT47_14160	pirin	VT47_22090	bacteriocin

VT47_14220	LysR family transcriptional regulator	VT47_22135	hypothetical protein
VT47_14225	MFS transporter	VT47_22245	hypothetical protein
VT47_14230	luciferase	VT47_22250	hypothetical protein
VT47_14235	luciferase	VT47_22300	integrase
VT47_14240	hypothetical protein	VT47_22305	plasmid-related protein
VT47_14245	hypothetical protein	VT47_22310	phage replication protein
VT47_14250	2-nitropropane dioxygenase	VT47_22315	conjugal transfer protein TrbJ
VT47_14255	antibiotic biosynthesis monooxygenase	VT47_22320	conjugal transfer protein TrbJ
VT47_14260	dimethylmenaquinone methyltransferase	VT47_22330	hypothetical protein
VT47_14265	3-isopropylmalate dehydrogenase	VT47_22350	hypothetical protein
VT47_14270	nucleoid-structuring protein H-NS	VT47_22355	hypothetical protein
VT47_14275	LysR family transcriptional regulator	VT47_22360	HxlR family transcriptional regulator
VT47_14280	hypothetical protein	VT47_22365	2'-hydroxyisoflavone reductase
VT47_14285	hypothetical protein	VT47_22375	hypothetical protein
VT47_14290	hypothetical protein	VT47_22380	integrase
VT47_14295	hypothetical protein	VT47_22385	methionine sulfoxide reductase A
VT47_14300	hypothetical protein	VT47_22395	lysine transporter LysE
VT47_14305	hypothetical protein	VT47_22400	AraC family transcriptional regulator
VT47_14315	filamentous hemagglutinin	VT47_22415	acetyltransferase
VT47_14375	energy transducer TonB	VT47_22420	toxin/anti-toxin system, HipA-like toxin module
VT47_14385	hypothetical protein	VT47_22425	XRE family transcriptional regulator
VT47_14515	CdaR family transcriptional regulator	VT47_22625	hypothetical protein
VT47_14520	lysophospholipase	VT47_23010	hypothetical protein
VT47_14585	RND transporter	VT47_23050	hypothetical protein
VT47_14590	hemolysin D	VT47_23110	hypothetical protein

VT47_14595	ACR family transporter	VT47_23160	cystathionine beta-synthase
VT47_14600	membrane protein	VT47_23165	hypothetical protein
VT47_14605	MFS transporter	VT47_23170	hypothetical protein
VT47_14610	GntR family transcriptional regulator	VT47_23175	hypothetical protein
VT47_14980	AraC family transcriptional regulator	VT47_23180	phosphatidylserine decarboxylase
VT47_14985	alpha/beta hydrolase	VT47_23210	amidase
VT47_14995	histidine kinase	VT47_23235	LacI family transcriptional regulator
VT47_15015	hypothetical protein	VT47_23305	hypothetical protein
VT47_15040	aminotransferase DegT	VT47_23445	hypothetical protein
VT47_15045	polysaccharide biosynthesis protein	VT47_23590	Fic family protein
VT47_15050	glycosyl transferase family 2	VT47_23645	hypothetical protein
VT47_15055	phosphotyrosine protein phosphatase	VT47_23660	hypothetical protein
VT47_15065	transcriptional regulator	VT47_23665	hypothetical protein
VT47_15070	glycosyl transferase family 2	VT47_23675	hypothetical protein
VT47_15075	glycosyl transferase family 1	VT47_23680	hypothetical protein
VT47_15080	glycosyl transferase family 1	VT47_23690	phosphoglycerate kinase
VT47_15085	glycosyl transferase	VT47_23695	hypothetical protein
VT47_15090	capsule biosynthesis protein CapH	VT47_23920	Sel1 repeat-containing protein
VT47_15095	capsular biosynthesis protein	VT47_23940	hypothetical protein
VT47_15235	hypothetical protein	VT47_23955	hypothetical protein
VT47_15635	pyridine nucleotide-disulfide oxidoreductase	VT47_23960	hypothetical protein
VT47_15705	chemotaxis protein CheY	VT47_23965	hypothetical protein
VT47_15820	DNA-3-methyladenine glycosylase	VT47_23970	hypothetical protein
VT47_15910	hypothetical protein	VT47_24005	type IV secretion protein Rhs
VT47_15920	hypothetical protein	VT47_24275	hypothetical protein

VT47_15930	hypothetical protein	VT47_24605	chemotaxis protein
VT47_16055	CAAX protease	VT47_24620	camphor resistance protein CrcB
VT47_16075	hypothetical protein	VT47_24625	chromate transporter
VT47_16120	prevent-host-death protein	VT47_24755	type I restriction-modification system, M [illegible]
VT47_16125	twitching motility protein PilT	VT47_24765	hypothetical protein
VT47_16285	hypothetical protein	VT47_24770	deoxyribonuclease HsdR
VT47_16555	MFS transporter permease	VT47_24775	metal-dependent hydrolase
VT47_16560	hypothetical protein	VT47_24780	hypothetical protein
VT47_16565	membrane protein	VT47_24785	transposase
VT47_16720	AraC family transcriptional regulator	VT47_24790	transposase
VT47_17050	sulfonate ABC transporter ATP-[illegible]	VT47_24795	hypothetical protein
VT47_17055	ABC transporter permease	VT47_24800	haloacid dehalogenase
VT47_17090	biotin transporter BioY	VT47_24805	hypothetical protein
VT47_17095	sulfonate ABC transporter substrate-[illegible]	VT47_24810	hypothetical protein
VT47_17170	thiopurine S-methyltransferase	VT47_24815	transposase
VT47_17660	histidine kinase	VT47_24820	transposase
VT47_17665	chemotaxis protein CheY	VT47_24825	hypothetical protein
VT47_17675	phosphoketolase	VT47_24830	hypothetical protein
VT47_17680	propionate kinase	VT47_24835	serine/threonine protein phosphatase
VT47_17685	universal stress protein	VT47_24840	plasmid stablization protein ParB
VT47_17695	ion transporter	VT47_24845	plasmid stablization protein ParB
VT47_17700	hypothetical protein	VT47_24850	serine recombinase
VT47_17705	GNAT family acetyltransferase	VT47_24885	non-ribosomal peptide synthetase
VT47_17715	hypothetical protein	VT47_24890	peptide synthetase
VT47_17720	hypothetical protein	VT47_24895	hypothetical protein

VT47_17745	hypothetical protein	VT47_24900	non-ribosomal peptide synthetase
VT47_17755	restriction endonuclease	VT47_24910	hypothetical protein
VT47_17760	ATPase	VT47_24915	hypothetical protein
VT47_17765	phage antirepressor protein	VT47_24920	non-ribosomal peptide synthetase
VT47_17780	membrane protein	VT47_24925	hypothetical protein
VT47_17785	chemotaxis protein	VT47_24930	hypothetical protein
VT47_17790	topoisomerase	VT47_24935	filamentous hemagglutinin
VT47_17845	CopG family transcriptional regulator	VT47_24940	hypothetical protein
VT47_17850	periplasmic or secreted lipoprotein	VT47_24945	hypothetical protein
VT47_17915	hypothetical protein	VT47_24960	hypothetical protein
VT47_17935	MFS transporter	VT47_24965	hypothetical protein
VT47_18000	hypothetical protein	VT47_24970	cytochrome B561
VT47_18080	hypothetical protein	VT47_24975	catalase
VT47_18085	LysR family transcriptional regulator	VT47_24980	quaternary ammonium transporter
VT47_18120	hypothetical protein	VT47_24985	transcriptional regulator
VT47_18125	hypothetical protein	VT47_25010	hypothetical protein
VT47_18135	hypothetical protein	VT47_25015	hypothetical protein
VT47_18140	hypothetical protein	VT47_25020	hypothetical protein
VT47_18145	hypothetical protein	VT47_25025	hypothetical protein
VT47_18150	hypothetical protein	VT47_25030	hypothetical protein
VT47_18155	hypothetical protein	VT47_25035	type IV secretion protein Rhs
VT47_18160	hypothetical protein	VT47_25040	hypothetical protein
VT47_18165	nuclease	VT47_25045	beta-lactamase TEM
VT47_18170	lipoprotein	VT47_25060	hypothetical protein
VT47_18175	hypothetical protein	VT47_25065	hypothetical protein

VT47_18185	prophage antirepressor, BRO-domain protein	VT47_25075	filamentous hemagglutinin
VT47_18250	antitoxin	VT47_25080	hypothetical protein
VT47_18385	transporter	VT47_25085	hypothetical protein
VT47_18390	amidinotransferase	VT47_25090	hypothetical protein
VT47_18395	biotin carboxylase	VT47_25095	transposase
VT47_18400	mangotoxin biosynthesis protein [illegible]	VT47_25105	hypothetical protein
VT47_18405	oxidoreductase	VT47_25110	hypothetical protein
VT47_18410	hypothetical protein		
VT47_18425	ligand-gated channel		
VT47_18455	zinc chelation protein SecC		
VT47_18715	hypothetical protein		
VT47_18720	hypothetical protein		
VT47_18730	glycosyl transferase family 51		
VT47_18740	FAD-binding protein		
VT47_18755	glucose dehydrogenase		
VT47_18790	polysaccharide deacetylase		
VT47_18795	amidase		
VT47_18800	GntR family transcriptional regulator		
VT47_19025	hypothetical protein		
VT47_19030	DNA helicase		
VT47_19260	insecticidal toxin complex protein C1		
VT47_19265	toxin		
VT47_19270	toxin		
VT47_19280	cytoplasmic protein		
VT47_19295	hypothetical protein		

VT47_19300	hypothetical protein
VT47_19500	hypothetical protein
VT47_19505	hypothetical protein
VT47_20120	prophage PssSM-03
VT47_20130	hypothetical protein
VT47_20140	hypothetical protein
VT47_20150	hypothetical protein
VT47_20160	hypothetical protein
VT47_20170	hypothetical protein
VT47_20175	hypothetical protein
VT47_20180	hypothetical protein
VT47_20190	Appr-1-p processing protein
VT47_20200	diguanylate cyclase
VT47_20215	chemotaxis protein
VT47_20230	amino acid amidase
VT47_20245	hypothetical protein
VT47_20375	toxin
VT47_20380	toxin
VT47_20390	peptidase M60-like domain protein
VT47_20585	hypothetical protein
VT47_20590	hypothetical protein
VT47_20595	BRO N-terminal domain-containing
VT47_20630	histone deacetylase superfamily protein
VT47_20640	DEAD/DEAH box helicase
VT47_20680	hypothetical protein

VT47_20690	hypothetical protein
VT47_20695	hypothetical protein
VT47_20700	hypothetical protein
VT47_20720	hypothetical protein
VT47_20735	molecular chaperone DnaJ
VT47_20780	hypothetical protein
VT47_20885	hypothetical protein
VT47_21215	transposase
VT47_21225	hypothetical protein
VT47_21285	hypothetical protein
VT47_21290	thioredoxin-like superfamily, group II [illegible]
VT47_21330	hypothetical protein
VT47_21470	hypothetical protein
VT47_21475	transposase
VT47_21500	ureidoglycolate lyase
VT47_21540	short-chain dehydrogenase
VT47_21555	beta-lactamase
VT47_21560	energy transducer TonB
VT47_21565	biopolymer transporter
VT47_21570	biopolymer transporter ExbD
VT47_21575	ligand-gated channel
VT47_21580	4-phytase
VT47_21625	hypothetical protein
VT47_21630	hypothetical protein
VT47_21635	thiamine biosynthesis protein ThiF

VT47_21640	hypothetical protein
VT47_21645	patatin
VT47_21650	hypothetical protein
VT47_21655	exonuclease V subunit alpha
VT47_22000	lysozyme
VT47_22015	tail fiber protein
VT47_22090	bacteriocin
VT47_22245	hypothetical protein
VT47_22250	hypothetical protein
VT47_22300	integrase
VT47_22305	plasmid-related protein
VT47_22310	phage replication protein
VT47_22315	conjugal transfer protein TrbJ
VT47_22320	conjugal transfer protein TrbJ
VT47_22325	conjugal transfer protein
VT47_22330	hypothetical protein
VT47_22335	RelE/ParE family plasmid stabilization
VT47_22340	prevent-host-death protein
VT47_22350	hypothetical protein
VT47_22355	hypothetical protein
VT47_22360	HxlR family transcriptional regulator
VT47_22365	2'-hydroxyisoflavone reductase
VT47_22375	hypothetical protein
VT47_22380	integrase
VT47_22385	methionine sulfoxide reductase A

VT47_22395	lysine transporter LysE
VT47_22400	AraC family transcriptional regulator
VT47_22405	phosphopantetheinyl transferase
VT47_22625	hypothetical protein
VT47_22630	transposase
VT47_22690	RHS repeat-associated core domain protein
VT47_22695	hypothetical protein
VT47_22705	YD repeat-containing protein
VT47_22940	ferredoxin
VT47_22950	chitinase
VT47_22955	PI-PLC X phosphodiestherase-like [illegible]
VT47_23025	glyceraldehyde-3-phosphate [illegible]
VT47_23050	hypothetical protein
VT47_23095	hypothetical protein
VT47_23160	cystathionine beta-synthase
VT47_23165	hypothetical protein
VT47_23170	hypothetical protein
VT47_23175	hypothetical protein
VT47_23180	phosphatidylserine decarboxylase
VT47_23210	amidase
VT47_23300	hypothetical protein
VT47_23445	hypothetical protein
VT47_23525	trypsin
VT47_23590	Fic family protein
VT47_23640	hypothetical protein

VT47_23645	hypothetical protein
VT47_23665	hypothetical protein
VT47_23675	hypothetical protein
VT47_23680	hypothetical protein
VT47_23690	phosphoglycerate kinase
VT47_23695	hypothetical protein
VT47_23715	acetyltransferase domain protein
VT47_23740	hypothetical protein
VT47_23825	hypothetical protein
VT47_23920	Sel1 repeat-containing protein
VT47_23950	hypothetical protein
VT47_23965	hypothetical protein
VT47_23980	hypothetical protein
VT47_23985	membrane protein
VT47_23995	hypothetical protein
VT47_24000	hypothetical protein
VT47_24005	type IV secretion protein Rhs
VT47_24015	membrane protein
VT47_24025	membrane protein
VT47_24040	mechanosensitive ion channel protein MscS
VT47_24160	lipoprotein
VT47_24275	hypothetical protein
VT47_24345	hypothetical protein
VT47_24460	membrane protein
VT47_24465	acyltransferase

VT47_24515	membrane protein
VT47_24535	histidinol phosphatase
VT47_24540	iron ABC transporter substrate-binding
VT47_24545	ABC transporter permease
VT47_24550	UDP-glucose 6-dehydrogenase
VT47_24555	protein CapI
VT47_24560	dolichol-phosphate mannosyltransferase
VT47_24565	Lipid A biosynthesis, N-terminal
VT47_24570	dolichyl-phosphate-mannose-protein
VT47_24575	SdiA-regulated
VT47_24590	NADPH-dependent FMN reductase
VT47_24595	ArsC family transcriptional regulator
VT47_24600	ArsR family transcriptional regulator
VT47_24605	chemotaxis protein
VT47_24610	inorganic pyrophosphatase
VT47_24615	hypothetical protein
VT47_24620	camphor resistance protein CrcB
VT47_24625	chromate transporter
VT47_24735	chromosomal replication initiation
VT47_24755	type I restriction-modification system, M
VT47_24765	hypothetical protein
VT47_24770	deoxyribonuclease HsdR
VT47_24775	metal-dependent hydrolase
VT47_24780	hypothetical protein
VT47_24785	transposase

VT47_24790	transposase
VT47_24795	hypothetical protein
VT47_24800	haloacid dehalogenase
VT47_24805	hypothetical protein
VT47_24810	hypothetical protein
VT47_24815	transposase
VT47_24820	transposase
VT47_24825	hypothetical protein
VT47_24830	hypothetical protein
VT47_24835	serine/threonine protein phosphatase
VT47_24840	plasmid stablization protein ParB
VT47_24845	plasmid stablization protein ParB
VT47_24850	serine recombinase
VT47_24885	non-ribosomal peptide synthetase
VT47_24890	peptide synthetase
VT47_24895	hypothetical protein
VT47_24900	non-ribosomal peptide synthetase
VT47_24910	hypothetical protein
VT47_24915	hypothetical protein
VT47_24920	non-ribosomal peptide synthetase
VT47_24925	hypothetical protein
VT47_24930	hypothetical protein
VT47_24935	filamentous hemagglutinin
VT47_24940	hypothetical protein
VT47_24945	hypothetical protein

VT47_24960	hypothetical protein
VT47_24965	hypothetical protein
VT47_24970	cytochrome B561
VT47_24975	catalase
VT47_24980	quaternary ammonium transporter
VT47_24985	transcriptional regulator
VT47_25010	hypothetical protein
VT47_25015	hypothetical protein
VT47_25020	hypothetical protein
VT47_25025	hypothetical protein
VT47_25030	hypothetical protein
VT47_25035	type IV secretion protein Rhs
VT47_25040	hypothetical protein
VT47_25045	beta-lactamase TEM
VT47_25060	hypothetical protein
VT47_25065	hypothetical protein
VT47_25070	YD repeat-containing protein
VT47_25075	filamentous hemagglutinin
VT47_25080	hypothetical protein
VT47_25085	hypothetical protein
VT47_25090	hypothetical protein
VT47_25110	hypothetical protein

ompared with *P. syringae* pv. *tomato* DC3000, *P. syringae* pv. *syringae* B728a, *P. syringae* CC1557, *P. syringae* pv. *syringae* HS191, *P. syringae* pv. *syringae* B64, *P. syringae* pv. *syringae* SM. The CDS for which no homologs were found in all six strains, were considered as strain specific.
