# Supplementary material for: Identification of the Genes of the Plant Pathogen Pseudomonas syringae MB03 Required for the Nematicidal Activity Against Caenorhabditis elegans Through an Integrated Approach
Source: Front Microbiol. 2022 Mar 9;13:826962. doi: 10.3389/fmicb.2022.826962 (PMC8959697; doi:10.3389/fmicb.2022.826962)
Supplement: Supplementary file 4 [file Data_Sheet_4.PDF]

Table S4. Prediction of genomic islands in *P. syringae* MB03 genome and distribution of genes into pan-genome.

Contig number	Length (bp)	Locus Tag	Strand	Product	Strain specific gene	Pan-genome	Pathogenicity [a]
3	8457	VT47_00420	-1	LuxR family transcriptional regulator		Auxiliary	
		VT47_00425	-1	anthranilate synthase subunit II		Auxiliary	
		VT47_00430	-1	anthranilate synthase	VT47_00430	Strain specific	
		VT47_00435	-1	hypothetical protein	VT47_00435	Strain specific	
		VT47_00440	-1	alpha/beta hydrolase	VT47_00440	Strain specific	
		VT47_00445	-1	hypothetical protein	VT47_00445	Strain specific	
		VT47_00450	-1	FMN reductase	VT47_00450	Strain specific	
		VT47_00455	-1	transcriptional regulator	VT47_00455	Strain specific	
		VT47_00460	1	hypothetical protein	VT47_00460	Strain specific	
		VT47_00465	-1	coenzyme F390 synthetase	VT47_00465	Strain specific	
8	6167	VT47_03675	-1	DNA topoisomerase III		Auxiliary	
		VT47_03680	1	LysR family transcriptional regulator		Auxiliary	
		VT47_03685	1	LuxR family transcriptional regulator		Auxiliary	
		VT47_03690	1	hypothetical protein		Auxiliary	
		VT47_03695	-1	lysine transporter LysE		Auxiliary	
		VT47_03700	-1	glycoside hydrolase		Auxiliary	
11	6129	VT47_04560	-1	glycosyltransferase		Auxiliary	

		VT47_04565	-1	hypothetical protein	VT47_04565	Strain specific	
		VT47_04570	-1	HAD superfamily hydrolase-like protein		Auxiliary	
		VT47_04575	-1	UDP-glucose 6-dehydrogenase		Auxiliary	
12	6348	VT47_06200	-1	NorR transcriptional regulator/ chemotaxis protein CheY		Auxiliary	
		VT47_06205	1	dihydropteridine reductase		Auxiliary	Transcriptomics
		VT47_06210	1	2-nitropropane dioxygenase		Auxiliary	Transcriptomics
		VT47_06215	1	tautomerase		Auxiliary	Transcriptomics
		VT47_06220	1	hypothetical protein		Auxiliary	
		VT47_06225	-1	hypothetical protein		Auxiliary	
12	9739	VT47_06930	1	aspartyl-tRNA synthetase		Core	
		VT47_06935	1	hypothetical protein		Core	Mutant library screening
		VT47_06940	1	crossover junction endodeoxyribonuclease RuvC		Core	
		VT47_06945	1	ATP-dependent DNA helicase RuvA		Core	
		VT47_06950	1	ATP-dependent DNA helicase RuvB		Core	
		VT47_06955	1	4-hydroxybenzoyl-CoA thioesterase		Core	
		VT47_06960	1	protein tolQ		Core	*P. aeruginosa* homolog
		VT47_06965	1	biopolymer transporter TolR		Core	
		VT47_06970	1	cell envelope biogenesis protein TolA		Auxiliary	
		VT47_06975	1	translocation protein TolB		Core	
		VT47_06980	1	peptidoglycan-binding protein		Core	

12	5706	VT47_07005	-1	integrase		Auxiliary
		VT47_07020	1	hypothetical protein	VT47_07020	Strain specific
		VT47_07025	-1	Clp protease		Auxiliary
12	18845	VT47_07080	-1	lipoprotein		Auxiliary
		VT47_07090	-1	hypothetical protein		Auxiliary
		VT47_07100	1	ompetence-damaged protein		Auxiliary
		VT47_07105	1	transposase		Auxiliary
		VT47_07110	1	transposase		Auxiliary
		VT47_07125	-1	phenazine biosynthesis protein PhzF		Auxiliary
		VT47_07130	-1	hypothetical protein	VT47_07130	Strain specific
		VT47_07135	1	hypothetical protein		Auxiliary
		VT47_07140	-1	membrane protein		Auxiliary
		VT47_07145	-1	hypothetical protein		Auxiliary
		VT47_07150	-1	conjugal transfer protein		Auxiliary
		VT47_07155	-1	conjugal transfer protein		Auxiliary
		VT47_07165	-1	aspartate carbamoyltransferase catalytic subunit	VT47_07165	Strain specific
		VT47_07170	-1	MFS transporter		Auxiliary
		VT47_07175	-1	methyltransferase type 11	VT47_07175	Strain specific
17	6480	VT47_08630	-1	GNAT family acetyltransferase		Core
		VT47_08635	1	hypothetical protein	VT47_08635	Strain specific

		VT47_08640	1	hypothetical protein	VT47_08640	Strain specific
		VT47_08645	1	hypothetical protein	VT47_08645	Strain specific
		VT47_08650	1	hypothetical protein		Auxiliary
		VT47_08655	1	hypothetical protein	VT47_08655	Strain specific
		VT47_08660	-1	type III effector HopAZ1		Auxiliary
18	27131	VT47_09780	-1	transposase		Auxiliary
		VT47_09785	-1	transposase		Auxiliary
		VT47_09795	-1	hypothetical protein	VT47_09795	Strain specific
		VT47_09800	-1	hypothetical protein	VT47_09800	Strain specific
		VT47_09805	-1	ProQ activator of osmoprotectant transporter prop	VT47_09805	Strain specific
		VT47_09810	1	hypothetical protein	VT47_09810	Strain specific
		VT47_09815	1	hypothetical protein	VT47_09815	Strain specific
		VT47_09820	1	hypothetical protein	VT47_09820	Strain specific
		VT47_09825	1	hypothetical protein	VT47_09825	Strain specific
		VT47_09830	-1	hypothetical protein		Auxiliary
		VT47_09835	1	serine/threonine protein phosphatase		Auxiliary
		VT47_09840	1	hypothetical protein	VT47_09840	Strain specific
		VT47_09850	1	hypothetical protein	VT47_09850	Strain specific
		VT47_09855	1	hypothetical protein		Auxiliary
		VT47_09860	-1	hypothetical protein	VT47_09860	Strain specific

		VT47_09865	-1	hypothetical protein	VT47_09865	Strain specific
		VT47_09870	-1	hypothetical protein		Auxiliary
		VT47_09875	-1	hypothetical protein	VT47_09875	Strain specific
		VT47_09880	-1	hypothetical protein	VT47_09880	Strain specific
19	7979	VT47_11235	-1	Major exported protein		Auxiliary
		VT47_11240	1	type VI secretion protein ImpA		Auxiliary
		VT47_11245	1	type VI secretion protein		Auxiliary
		VT47_11250	1	type VI secretion protein		Auxiliary
		VT47_11255	1	type VI secretion protein		Auxiliary
		VT47_11260	1	type VI secretion protein		Auxiliary
		VT47_11265	1	type VI secretion protein		Auxiliary
32	9345	VT47_13245	1	repressor		Core
		VT47_13250	1	hypothetical protein		Auxiliary
		VT47_13255	-1	prophage PssSM-03		Auxiliary
		VT47_13260	1	hypothetical protein		Auxiliary
		VT47_13265	1	chemotaxis protein		Auxiliary
		VT47_13270	1	hypothetical protein		Auxiliary
		VT47_13275	1	5-methyltetrahydropteroyltriglutamate--homocysteine methyltransferase		Auxiliary
		VT47_13280	-1	chitin-binding protein		Auxiliary
33	4689	VT47_14275	-1	LysR family transcriptional regulator		Auxiliary

		VT47_14280	-1	hypothetical protein		Auxiliary
		VT47_14285	-1	hypothetical protein	VT47_14285	Strain specific
		VT47_14290	-1	hypothetical protein	VT47_14290	Strain specific
		VT47_14295	-1	hypothetical protein	VT47_14295	Strain specific
		VT47_14300	-1	hypothetical protein	VT47_14300	Strain specific
36	5184	VT47_15910	1	hypothetical protein	VT47_15910	Strain specific
		VT47_15920	1	hypothetical protein	VT47_15920	Strain specific
		VT47_15930	-1	hypothetical protein		Auxiliary
		VT47_15935	-1	transposase		Auxiliary
42	10452	VT47_18120	-1	hypothetical protein	VT47_18120	Strain specific
		VT47_18125	1	hypothetical protein	VT47_18125	Strain specific
		VT47_18130	1	hypothetical protein		Auxiliary
		VT47_18135	1	hypothetical protein	VT47_18135	Strain specific
		VT47_18140	1	hypothetical protein		Auxiliary
		VT47_18145	-1	hypothetical protein	VT47_18145	Strain specific
		VT47_18150	-1	hypothetical protein	VT47_18150	Strain specific
		VT47_18155	1	hypothetical protein		Auxiliary
		VT47_18160	-1	hypothetical protein	VT47_18160	Strain specific
46	6033	VT47_21540	1	short-chain dehydrogenase		Auxiliary
		VT47_21545	-1	diguanylate phosphodiesterase		Auxiliary

		VT47_21550	-1	ABC transporter substrate-binding protein		Auxiliary	
		VT47_21555	1	beta-lactamase		Auxiliary	
46	7954	VT47_21625	-1	hypothetical protein	VT47_21625	Strain specific	
		VT47_21630	-1	hypothetical protein	VT47_21630	Strain specific	
		VT47_21635	-1	thiamine biosynthesis protein ThiF	VT47_21635	Strain specific	
		VT47_21640	-1	hypothetical protein	VT47_21640	Strain specific	
		VT47_21645	-1	patatin	VT47_21645	Strain specific	
		VT47_21650	1	hypothetical protein	VT47_21650	Strain specific	
		VT47_21655	-1	exonuclease V subunit alpha	VT47_21655	Strain specific	
47	23656	VT47_22280	1	DNA primase		Core	
		VT47_22285	1	RNA polymerase subunit sigma-70		Auxiliary	
		VT47_22290	1	diguanylate phosphodiesterase		Core	
		VT47_22300	1	integrase		Auxiliary	
		VT47_22305	1	plasmid-related protein		Auxiliary	
		VT47_22310	1	phage replication protein	VT47_22310	Strain specific	
		VT47_22315	1	conjugal transfer protein TrbJ		Auxiliary	
		VT47_22320	1	conjugal transfer protein TrbJ		Auxiliary	Transcriptomics
		VT47_22325	1	conjugal transfer protein		Auxiliary	
		VT47_22330	1	hypothetical protein	VT47_22330	Strain specific	
		VT47_22335	-1	RelE/ParE family plasmid stabilization system protein		Auxiliary	

		VT47_22340	-1	prevent-host-death protein		Auxiliary
		VT47_22345	1	integrase		Auxiliary
		VT47_22350	-1	hypothetical protein		Auxiliary
		VT47_22355	-1	hypothetical protein		Auxiliary
		VT47_22360	-1	HxlR family transcriptional regulator		Auxiliary
		VT47_22365	1	2'-hydroxyisoflavone reductase		Auxiliary
		VT47_22375	-1	hypothetical protein		Auxiliary
		VT47_22380	-1	integrase	VT47_22380	Strain specific
		VT47_22385	-1	methionine sulfoxide reductase A		Auxiliary
		VT47_22390	1	formate/nitrite transporter		Auxiliary
		VT47_22395	-1	lysine transporter LysE		Auxiliary
		VT47_22400	-1	AraC family transcriptional regulator		Auxiliary
49	6170	VT47_23160	-1	cystathionine beta-synthase		Auxiliary
		VT47_23165	-1	hypothetical protein	VT47_23165	Strain specific
		VT47_23170	-1	hypothetical protein	VT47_23170	Strain specific
		VT47_23175	-1	hypothetical protein	VT47_23175	Strain specific
		VT47_23180	-1	phosphatidylserine decarboxylase	VT47_23180	Strain specific
49	6393	VT47_23665	1	hypothetical protein	VT47_23665	Strain specific
		VT47_23675	1	hypothetical protein	VT47_23675	Strain specific
		VT47_23680	1	hypothetical protein	VT47_23680	Strain specific

		VT47_23685	-1	hypothetical protein	Auxiliary	
		VT47_23690	-1	phosphoglycerate kinase	Auxiliary	
		VT47_23695	-1	hypothetical protein	Auxiliary	
		VT47_23700	1	prophage PssSM-03	Auxiliary	
49	4389	VT47_23910	1	PAAR domain-containing protein	Auxiliary	
		VT47_23915	1	phospholipase	Auxiliary	
		VT47_23920	1	Sel1 repeat-containing protein	Auxiliary	
		VT47_23925	1	Sel1 repeat-containing protein	Auxiliary	
49	6636	VT47_23940	-1	hypothetical protein	Auxiliary	
		VT47_23945	-1	guanosine polyphosphate pyrophosphohydrolase	Auxiliary	
		VT47_23950	-1	hypothetical protein	Auxiliary	
		VT47_23955	-1	hypothetical protein	Auxiliary	
		VT47_23960	-1	hypothetical protein	Auxiliary	
		VT47_23965	-1	hypothetical protein	Auxiliary	Transcriptomics
		VT47_23970	-1	hypothetical protein	Auxiliary	
		VT47_23980	1	hypothetical protein	Auxiliary	
		VT47_23985	1	membrane protein	Auxiliary	
52	8237	VT47_24585	1	PEP phosphonomutase	Auxiliary	
		VT47_24590	-1	NADPH-dependent FMN reductase	Auxiliary	
		VT47_24595	-1	ArsC family transcriptional regulator	Auxiliary	

		VT47_24600	-1	ArsR family transcriptional regulator		Auxiliary
		VT47_24605	1	chemotaxis protein		Auxiliary
		VT47_24610	-1	inorganic pyrophosphatase		Auxiliary
		VT47_24615	-1	hypothetical protein		Auxiliary
		VT47_24620	-1	camphor resistance protein CrcB		Auxiliary
		VT47_24625	-1	chromate transporter		Auxiliary
52	18260	VT47_24765	1	hypothetical protein		Auxiliary
		VT47_24770	1	deoxyribonuclease HsdR		Auxiliary
		VT47_24775	1	metal-dependent hydrolase		Auxiliary
		VT47_24780	1	hypothetical protein	VT47_24780	Strain specific
		VT47_24785	1	transposase		Auxiliary
		VT47_24790	1	transposase		Auxiliary
		VT47_24795	1	hypothetical protein		Auxiliary
		VT47_24800	1	haloacid dehalogenase		Auxiliary
		VT47_24805	-1	hypothetical protein		Auxiliary
		VT47_24810	1	hypothetical protein		Auxiliary
		VT47_24815	1	transposase		Auxiliary
		VT47_24820	1	transposase		Auxiliary
		VT47_24825	1	hypothetical protein		Auxiliary
		VT47_24830	1	hypothetical protein	VT47_24830	Strain specific

		VT47_24835	1	serine/threonine protein phosphatase		Auxiliary
		VT47_24840	-1	plasmid stablization protein ParB		Auxiliary
67	6040	VT47_25110	1	hypothetical protein		Auxiliary
		VT47_25105	1	hypothetical protein		Auxiliary
		VT47_25040	-1	hypothetical protein	VT47_25040	Strain specific
		VT47_25035	-1	type IV secretion protein Rhs		Auxiliary
		VT47_24015	1	membrane protein		Auxiliary

[a] Pathogenicity of a gene was proposed based on its identification in any of the four approaches used in this study.
