# Supplementary material for: Identification of the Genes of the Plant Pathogen Pseudomonas syringae MB03 Required for the Nematicidal Activity Against Caenorhabditis elegans Through an Integrated Approach
Source: Front Microbiol. 2022 Mar 9;13:826962. doi: 10.3389/fmicb.2022.826962 (PMC8959697; doi:10.3389/fmicb.2022.826962)
Supplement: Supplementary file 5 [file Data_Sheet_9.PDF]

Table S5. Genes of *P. syringae* MB03 with high differential expression during exponential and stationary phase interaction.

Exponential Phase Interaction						Stationary Phase Interaction					
**gene_id**	**gene_name**	**log2FC1(Sample12C/Sample1**	**up-down-**	**NR_tophit_name**	**NR_tophit_description**	**gene_id**	**gene_name**	**log2FC1(Sample24C/Sample24**	**up-down-**	**NR_tophit_name**	**NR_tophit_description**
gnl|PRJNA278487|VT47_10310		3.383936613	up	gi|489440555|ref|WP_003346032.1|	periplasmic binding protein/LacI transcriptional regulator [*P. syringae*]	gnl|PRJNA278487|VT47_06590		6.426957945	up	gi|489517218|ref|WP_003422039.1|	hypothetical protein [*P. syringae*]
gnl|PRJNA278487|VT47_15265		3.246627325	up	gi|514421326|ref|WP_016568489.1|	(Myo)inositol ABC-type transport system, ATP-binding protein [*P.*	gnl|PRJNA278487|VT47_15130	atoE	6.198545537	up	gi|489515638|ref|WP_003420472.1|	Short chain fatty acid transporter [*P. syringae*]
gnl|PRJNA278487|VT47_09390		3.200334469	up	gi|489515052|ref|WP_003419888.1|	2-dehydro-3-deoxy-6-phosphogalactonate aldolase	gnl|PRJNA278487|VT47_13020		5.850354911	up	gi|489440889|ref|WP_003346362.1|	LysR family transcriptional regulator [*P. syringae*]
gnl|PRJNA278487|VT47_07185		3.178515796	up	gi|489523145|ref|WP_003427928.1|	hypothetical protein [*P. syringae*]	gnl|PRJNA278487|VT47_11145		5.769362936	up	gi|490552874|ref|WP_004417981.1|	putative NAD(FAD)-dependent dehydrogenase in
gnl|PRJNA278487|VT47_18935		3.131169024	up	gi|489508156|ref|WP_003413024.1|	C4-dicarboxylate ABC transporter substrate-binding protein [*P.*	gnl|PRJNA278487|VT47_18790		5.518835377	up	gi|66047164|ref|YP_237005.1|	polysaccharide deacetylase [*P. syringae* pv. *syringae* B728a]
gnl|PRJNA278487|VT47_18940		3.1274786	up	gi|489520482|ref|WP_003425277.1|	membrane protein TctB [*P. syringae*]	gnl|PRJNA278487|VT47_22965	fixB	5.494911738	up	gi|489522248|ref|WP_003427036.1|	electron transfer flavoprotein subunit alpha [*P. syringae*]
gnl|PRJNA278487|VT47_07180	gsiB	3.120944159	up	gi|489413048|ref|WP_003318984.1|	hypothetical protein [*P. syringae*]	gnl|PRJNA278487|VT47_22635		5.171542185	up	gi|66047932|ref|YP_237773.1|	AraC family transcriptional regulator [*P. syringae* pv.
gnl|PRJNA278487|VT47_24115	pntA	3.088069011	up	gi|66048241|ref|YP_238082.1|	NAD(P) transhydrogenase subunit alpha [*P. syringae* pv. *syringae*	gnl|PRJNA278487|VT47_24345		5.155687245	up	gi|489410069|ref|WP_003316060.1|	hypothetical protein [*P. syringae*]
gnl|PRJNA278487|VT47_09385	dgoK	2.995527571	up	gi|490553211|ref|WP_004418318.1|	2-dehydro-3-deoxygalactonokinase [P. syringae]	gnl|PRJNA278487|VT47_05955		5.155687245	up	gi|490537938|ref|WP_004403088.1|	type III secretion protein HrcQa [*P. syringae*]
gnl|PRJNA278487|VT47_24110	pntA	2.962334718	up	gi|489516551|ref|WP_003421380.1|	NAD(P) transhydrogenase subunit alpha [*P. syringae*]	gnl|PRJNA278487|VT47_11890	gnyD/IVD	4.811156312	up	gi|514420916|ref|WP_016568079.1|	Isovaleryl-CoA dehydrogenase [*P. syringae*]
gnl|PRJNA278487|VT47_04870		2.934206123	up	gi|498110609|ref|WP_010424765.1|	hypothetical protein, partial [*P. syringae*]	gnl|PRJNA278487|VT47_11875		4.477130649	up	gi|490539571|ref|WP_004404714.1|	3-methylcrotonyl-CoA carboxylase alpha subunit [*P.*
gnl|PRJNA278487|VT47_10315	rbsA	2.841691753	up	gi|489409584|ref|WP_003315577.1|	ABC transporter [*Pseudomonas* ABC transporter [*P. syringae* pv. *japonica*	gnl|PRJNA278487|VT47_11885		4.354064464	up	gi|489412579|ref|WP_003318524.1|	propionyl-CoA carboxylase [*P. syringae*]
gnl|PRJNA278487|VT47_10470	gsiB	2.792468381	up	gi|489413048|ref|WP_003318984.1|	hypothetical protein [P. syringae]	gnl|PRJNA278487|VT47_11880	liuC	3.808728136	up	gi|514420914|ref|WP_016568077.1|	Gamma-carboxygeranoyl-CoA hydratase [*P. syringae*]
gnl|PRJNA278487|VT47_15270		2.752220291	up	gi|489409780|ref|WP_003315773.1|	sugar ABC transporter periplasmic sugar-binding protein [*P. syringae*]	gnl|PRJNA278487|VT47_03060		3.731135083	up	gi|498093628|ref|WP_010407784.1|	ABC transporter [*P. syringae*]
gnl|PRJNA278487|VT47_20030		2.744582198	up	gi|490545314|ref|WP_004410438.1|	transcription elongation factor GreB [*P. syringae*]	gnl|PRJNA278487|VT47_18775	fabG	3.728232668	up	gi|66047161|ref|YP_237002.1|	Short-chain dehydrogenase/reductase SDR
gnl|PRJNA278487|VT47_01010	citN	2.721541521	up	gi|66043468|ref|YP_233309.1|	citrate transporter [*P. syringae* pv. *syringae* B728a]	gnl|PRJNA278487|VT47_03075		3.563304494	up	gi|489517490|ref|WP_003422306.1|	HAD family hydrolase [*P. syringae*]
gnl|PRJNA278487|VT47_02210	mdcA	2.702761009	up	gi|489498052|ref|WP_003402963.1|	malonate decarboxylase subunit alpha [P. syringae]	gnl|PRJNA278487|VT47_11730	smoG, mtlG	3.304032385	up	gi|489441722|ref|WP_003347187.1|	mannitol ABC transporter permease [*P. syringae*]
gnl|PRJNA278487|VT47_18945	tctA	2.683914478	up	gi|489520475|ref|WP_003425270.1|	hypothetical protein [*P. syringae*]	gnl|PRJNA278487|VT47_08190		3.267604232	up	gi|489485696|ref|WP_003390667.1|	ABC transporter ATP-binding protein [*P. syringae*]
gnl|PRJNA278487|VT47_10495		2.590321792	up	gi|514420749|ref|WP_016567912.1|	Short-chain dehydrogenase/reductase (SDR) family oxidoreductase	gnl|PRJNA278487|VT47_03055		3.231217861	up	gi|489411262|ref|WP_003317235.1|	hypothetical protein [*P. syringae*]
gnl|PRJNA278487|VT47_10500		2.582500306	up	gi|489528253|ref|WP_003432995.1|	hypothetical protein [*P. syringae*]	gnl|PRJNA278487|VT47_18480	hisJ	3.216548036	up	gi|489524009|ref|WP_003428785.1|	extracellular solute-binding protein [*P. syringae*]

<table>
<tr><td>gnl|PRJNA278487|VT47_20505</td><td>ybdD</td><td>2.520483584</td><td>up</td><td>gi|489407801|ref|WP_003313802.1|</td><td>hypothetical protein [P. syringae]</td></tr>
<tr><td>gnl|PRJNA278487|VT47_09395</td><td>dgoD</td><td>2.516272976</td><td>up</td><td>gi|498112168|ref|WP_010426324.1|</td><td>galactonate dehydratase, partial [P. syringae]</td></tr>
<tr><td>gnl|PRJNA278487|VT47_10190</td><td>oprD</td><td>2.512033386</td><td>up</td><td>gi|490553107|ref|WP_004418214.1|</td><td>Outer membrane porin, OprD family [P. syringae]</td></tr>
<tr><td>gnl|PRJNA278487|VT47_15130</td><td>atoE</td><td>2.497333631</td><td>up</td><td>gi|489515638|ref|WP_003420472.1|</td><td>Short chain fatty acid transporter [P. syringae]</td></tr>
<tr><td>gnl|PRJNA278487|VT47_10490</td><td>GntR</td><td>2.493178763</td><td>up</td><td>gi|489518999|ref|WP_003423803.1|</td><td>transcriptional regulator GntR [P. syringae]</td></tr>
<tr><td>gnl|PRJNA278487|VT47_17900</td><td>actP</td><td>2.40308425</td><td>up</td><td>gi|498107443|ref|WP_010421599.1|</td><td>acetate permease [P. syringae]</td></tr>
<tr><td>gnl|PRJNA278487|VT47_22115</td><td>estA</td><td>2.388178734</td><td>up</td><td>gi|489514889|ref|WP_003419726.1|</td><td>esterase [P. syringae]</td></tr>
<tr><td>gnl|PRJNA278487|VT47_18645</td><td>gltI</td><td>2.340834184</td><td>up</td><td>gi|71735103|ref|YP_276039.1|</td><td>amino acid ABC transporter substrate-binding protein [P. syringae pv.</td></tr>
<tr><td>gnl|PRJNA278487|VT47_07350</td><td>PSPPH_1548</td><td>2.335374262</td><td>up</td><td>gi|71736606|ref|YP_273800.1|</td><td>hypothetical protein PSPPH_1548 [P. syringae pv. phaseolicola 1448A]</td></tr>
<tr><td>gnl|PRJNA278487|VT47_23245</td><td>hutU</td><td>2.290637249</td><td>up</td><td>gi|28872382|ref|NP_795001.1|</td><td>urocanate hydratase [P. syringae pv. tomato str. DC3000]</td></tr>
<tr><td>gnl|PRJNA278487|VT47_22635</td><td></td><td>2.285584412</td><td>up</td><td>gi|66047932|ref|YP_237773.1|</td><td>AraC family transcriptional regulator [P. syringae pv. syringae B728a]</td></tr>
<tr><td>gnl|PRJNA278487|VT47_20330</td><td>dppA</td><td>2.278519561</td><td>up</td><td>gi|490550970|ref|WP_004416086.1|</td><td>Dipeptide ABC-type transport system, periplasmic substrate-binding protein</td></tr>
<tr><td>gnl|PRJNA278487|VT47_04875</td><td></td><td>2.260401914</td><td>up</td><td>gi|490537562|ref|WP_004402712.1|</td><td>hypothetical protein [P. syringae]</td></tr>
<tr><td>gnl|PRJNA278487|VT47_09400</td><td>dgoT</td><td>2.231817953</td><td>up</td><td>gi|489410547|ref|WP_003316531.1|</td><td>D-galactonate transporter [P. syringae]</td></tr>
<tr><td>gnl|PRJNA278487|VT47_24120</td><td>pntB</td><td>2.202556168</td><td>up</td><td>gi|489528100|ref|WP_003432845.1|</td><td>NAD(P)(+) transhydrogenase [P. syringae]</td></tr>
<tr><td>gnl|PRJNA278487|VT47_02225</td><td>mdcD</td><td>2.188168568</td><td>up</td><td>gi|514419834|ref|WP_016566997.1|</td><td>Malonate decarboxylase, beta subunit [P. syringae]</td></tr>
<tr><td>gnl|PRJNA278487|VT47_10320</td><td>rbsC</td><td>2.186050586</td><td>up</td><td>gi|514420739|ref|WP_016567902.1|</td><td>Ribose ABC-type transport system, permease protein RbsC [P. syringae]</td></tr>
<tr><td>gnl|PRJNA278487|VT47_15125</td><td>scoB</td><td>2.179559113</td><td>up</td><td>gi|66046467|ref|YP_236308.1|</td><td>3-oxoacid CoA-transferase [P. syringae pv. syringae B728a]</td></tr>
<tr><td>gnl|PRJNA278487|VT47_18650</td><td>gltJ</td><td>2.175973467</td><td>up</td><td>gi|489513682|ref|WP_003418523.1|</td><td>amino acid ABC transporter permease [P. syringae]</td></tr>
<tr><td>gnl|PRJNA278487|VT47_18655</td><td>gltK</td><td>2.161489676</td><td>up</td><td>gi|489524053|ref|WP_003428829.1|</td><td>amino acid ABC transporter permease [P. syringae]</td></tr>
<tr><td>gnl|PRJNA278487|VT47_02990</td><td>livK</td><td>2.150193608</td><td>up</td><td>gi|489411246|ref|WP_003317220.1|</td><td>extracellular ligand-binding receptor [P. syringae]</td></tr>
<tr><td>gnl|PRJNA278487|VT47_18660</td><td>gltL</td><td>2.124871494</td><td>up</td><td>gi|489458394|ref|WP_003363626.1|</td><td>arginine ABC transporter ATP-binding protein [P. syringae]</td></tr>
<tr><td>gnl|PRJNA278487|VT47_02220</td><td>mdcC</td><td>2.037246405</td><td>up</td><td>gi|489440848|ref|WP_003346322.1|</td><td>malonate decarboxylase subunit delta [P. syringae]</td></tr>
<tr><td>gnl|PRJNA278487|VT47_06005</td><td>CesT</td><td>2.037246405</td><td>up</td><td>gi|66044473|ref|YP_234314.1|</td><td>hypothetical protein Psyr_1225 [P. syringae pv. syringae B728a]</td></tr>
</table>

<table>
<tr><td>gnl|PRJNA278487|VT47_08495</td><td>tauD</td><td>3.089487906</td><td>up</td><td>gi|489443513|ref|WP_003348961.1|</td><td>taurine dioxygenase [P. syringae]</td></tr>
<tr><td>gnl|PRJNA278487|VT47_08195</td><td></td><td>3.075089767</td><td>up</td><td>gi|28870893|ref|NP_793512.1|</td><td>ABC transporter permease [P. syringae pv. tomato str.</td></tr>
<tr><td>gnl|PRJNA278487|VT47_18780</td><td>fabG</td><td>3.073540074</td><td>up</td><td>gi|498106706|ref|WP_010420862.1|</td><td>glucose 1-dehydrogenase, putative, partial [P. syringae]</td></tr>
<tr><td>gnl|PRJNA278487|VT47_12800</td><td></td><td>2.987729402</td><td>up</td><td>gi|490552561|ref|WP_004417669.1|</td><td>Protein of unknown function (DUF1446) [P. syringae]</td></tr>
<tr><td>gnl|PRJNA278487|VT47_22320</td><td></td><td>2.987729402</td><td>up</td><td>gi|489517885|ref|WP_003422695.1|</td><td>conjugal transfer protein TrbJ [P. syringae]</td></tr>
<tr><td>gnl|PRJNA278487|VT47_16420</td><td>flgG</td><td>2.972098541</td><td>up</td><td>gi|489499430|ref|WP_003404338.1|</td><td>flagellar basal body rod protein FlgG [P. syringae]</td></tr>
<tr><td>gnl|PRJNA278487|VT47_15285</td><td>iolG</td><td>2.898866164</td><td>up</td><td>gi|489522319|ref|WP_003427107.1|</td><td>myo-inositol 2-dehydrogenase [P. syringae]</td></tr>
<tr><td>gnl|PRJNA278487|VT47_18345</td><td>ompA</td><td>2.896490172</td><td>up</td><td>gi|489488418|ref|WP_003393373.1|</td><td>OmpA/MotB [P. syringae]</td></tr>
<tr><td>gnl|PRJNA278487|VT47_23710</td><td></td><td>2.896490172</td><td>up</td><td>gi|498118884|ref|WP_010433040.1|</td><td>GNAT family acetyltransferase [P. syringae]</td></tr>
<tr><td>gnl|PRJNA278487|VT47_06530</td><td></td><td>2.873399999</td><td>up</td><td>gi|489408281|ref|WP_003314274.1|</td><td>putative lipoprotein [P. syringae]</td></tr>
<tr><td>gnl|PRJNA278487|VT47_05225</td><td>algE</td><td>2.85697829</td><td>up</td><td>gi|489410959|ref|WP_003316937.1|</td><td>alginate biosynthesis protein AlgE [P. syringae]</td></tr>
<tr><td>gnl|PRJNA278487|VT47_15310</td><td></td><td>2.846997706</td><td>up</td><td>gi|489442557|ref|WP_003348013.1|</td><td>AP endonuclease [Pseudomonas AP</td></tr>
<tr><td>gnl|PRJNA278487|VT47_24480</td><td>tauB</td><td>2.8105728</td><td>up</td><td>gi|489516654|ref|WP_003421482.1|</td><td>ABC transporter [Pseudomonas ABC</td></tr>
<tr><td>gnl|PRJNA278487|VT47_08995</td><td></td><td>2.799088876</td><td>up</td><td>gi|489409096|ref|WP_003315089.1|</td><td>hypothetical protein [P. syringae]</td></tr>
<tr><td>gnl|PRJNA278487|VT47_09960</td><td>pabB</td><td>2.799088876</td><td>up</td><td>gi|490545329|ref|WP_004410453.1|</td><td>aminodeoxychorismate synthase [P. syringae]</td></tr>
<tr><td>gnl|PRJNA278487|VT47_16450</td><td>flgC</td><td>2.793245058</td><td>up</td><td>gi|28869138|ref|NP_791757.1|</td><td>flagellar basal body rod protein FlgC [P. syringae pv. tomato</td></tr>
<tr><td>gnl|PRJNA278487|VT47_02330</td><td>mmsB</td><td>2.793194681</td><td>up</td><td>gi|66043733|ref|YP_233574.1|</td><td>3-hydroxyisobutyrate dehydrogenase [P. syringae</td></tr>
<tr><td>gnl|PRJNA278487|VT47_03065</td><td></td><td>2.793194681</td><td>up</td><td>gi|498093631|ref|WP_010407787.1|</td><td>nucleotidyl transferase [P. syringae]</td></tr>
<tr><td>gnl|PRJNA278487|VT47_18175</td><td></td><td>2.789851096</td><td>up</td><td>gi|489507939|ref|WP_003412808.1|</td><td>hypothetical protein [P. syringae]</td></tr>
<tr><td>gnl|PRJNA278487|VT47_09990</td><td>prpC</td><td>2.732894872</td><td>up</td><td>gi|514420644|ref|WP_016567807.1|</td><td>2-methylcitrate synthase [P. syringae]</td></tr>
<tr><td>gnl|PRJNA278487|VT47_05400</td><td>gcvP</td><td>2.724129182</td><td>up</td><td>gi|490537748|ref|WP_004402898.1|</td><td>glycine dehydrogenase [P. syringae]</td></tr>
<tr><td>gnl|PRJNA278487|VT47_24485</td><td>tauC</td><td>2.680480093</td><td>up</td><td>gi|489410031|ref|WP_003316022.1|</td><td>binding-protein dependent transport system inner</td></tr>
<tr><td>gnl|PRJNA278487|VT47_16625</td><td></td><td>2.679272836</td><td>up</td><td>gi|489408790|ref|WP_003314783.1|</td><td>aldehyde dehydrogenase [P. syringae]</td></tr>
<tr><td>gnl|PRJNA278487|VT47_11145</td><td></td><td>2.670348219</td><td>up</td><td>gi|490552874|ref|WP_004417981.1|</td><td>putative NAD(FAD)-dependent dehydrogenase in</td></tr>
</table>

gnl|PRJNA278487|VT47_02845		2.037246405	up	gi|489488587|ref|WP_003393541.1|	hypothetical protein [*P. syringae*]	gnl|PRJNA278487|VT47_18500	hisP	2.667222112	up	gi|66047105|ref|YP_236946.1|	ABC transporter [*P. syringae* pv. *syringae* B728a]
gnl|PRJNA278487|VT47_02540	putP	2.036984966	up	gi|498105688|ref|WP_010419844.1|	Na+/solute symporter [*P. syringae*]	gnl|PRJNA278487|VT47_11720		2.648006147	up	gi|498097310|ref|WP_010411466.1|	mannitol dehydrogenase [*P. syringae*]
gnl|PRJNA278487|VT47_15305	iolB	2.018936738	up	gi|489442555|ref|WP_003348011.1|	5-deoxyglucuronate isomerase [*P. syringae*]	gnl|PRJNA278487|VT47_09995	acnA	2.567241376	up	gi|514420645|ref|WP_016567808.1|	2-methylcitrate dehydratase, Fe-S dependent [*P. syringae*]
gnl|PRJNA278487|VT47_18480	argT	2.013551543	up	gi|489524009|ref|WP_003428785.1|	extracellular solute-binding protein [*P. syringae*]	gnl|PRJNA278487|VT47_05395	gcvT	2.51126579	up	gi|490537746|ref|WP_004402896.1|	glycine cleavage system T protein [*P. syringae*]
gnl|PRJNA278487|VT47_11195		-2.018086287	down	gi|489519322|ref|WP_003424123.1|	hypothetical protein [*P. syringae*]	gnl|PRJNA278487|VT47_20340		2.473083068	up	gi|489407761|ref|WP_003313762.1|	outer membrane porin [*P. syringae*]
gnl|PRJNA278487|VT47_01705		-2.024425427	down	gi|489411333|ref|WP_003317305.1|	hypothetical protein [*P. syringae*]	gnl|PRJNA278487|VT47_22655	glyA	2.470196983	up	gi|498092921|ref|WP_010407077.1|	serine hydroxymethyltransferase [*P.*
gnl|PRJNA278487|VT47_02205		-2.024425427	down	gi|492186337|ref|WP_005772978.1|	hypothetical protein, partial [*P. amygdali*]	gnl|PRJNA278487|VT47_24115	pntA	2.459864383	up	gi|66048241|ref|YP_238082.1|	NAD(P) transhydrogenase subunit alpha [*P. syringae* pv.
gnl|PRJNA278487|VT47_13300		-2.024425427	down	gi|490803134|ref|WP_004665266.1|	hypothetical protein [*P. amygdali*]	gnl|PRJNA278487|VT47_12495		2.459864383	up	gi|489515665|ref|WP_003420499.1|	hypothetical protein [*P. syringae*]
gnl|PRJNA278487|VT47_21225		-2.090258305	down	gi|387613266|ref|YP_006116382.1|	hypothetical protein ETEC_2829 [*Escherichia coli* ETEC H10407]	gnl|PRJNA278487|VT47_16455	flgB	2.446954422	up	gi|489410798|ref|WP_003316778.1|	flagellar basal body rod protein FlgB [*P. syringae*]
gnl|PRJNA278487|VT47_03545	rpsT	-2.091179568	down	gi|488615127|ref|WP_002551976.1|	30S ribosomal protein S20 [*P. savastanoi*]	gnl|PRJNA278487|VT47_09645	fimA	2.419895008	up	gi|489518811|ref|WP_003423616.1|	fimbrial protein [*Pseudomonas* fimbrial protein] [*P. syringae*
gnl|PRJNA278487|VT47_06215	yrdN	-2.108612611	down	gi|489408206|ref|WP_003314200.1|	4-oxalocrotonate tautomerase [*P. syringae*]	gnl|PRJNA278487|VT47_16520		2.415827958	up	gi|489410783|ref|WP_003316764.1|	hypothetical protein [*P. syringae*]
gnl|PRJNA278487|VT47_15510		-2.164241715	down	gi|490552058|ref|WP_004417168.1|	Tellurite-resistance/dicarboxylate transporter (TDT) family protein [*P.*	gnl|PRJNA278487|VT47_17630	phoQ	2.387675255	up	gi|490551619|ref|WP_004416732.1|	Two component sensor histidine kinase PhoQ [*P.*
gnl|PRJNA278487|VT47_13700		-2.181100186	down	gi|489518055|ref|WP_003422865.1|	amino acid ABC transporter permease [*P. syringae*]	gnl|PRJNA278487|VT47_16345	fleS	2.361099648	up	gi|66046689|ref|YP_236530.1|	flagellar sensor histidine kinase FleS [*P. syringae* pv. *syringae*
gnl|PRJNA278487|VT47_14430	nasB	-2.189749074	down	gi|489521484|ref|WP_003426274.1|	FAD-dependent pyridine nucleotide-disulfide oxidoreductase [*P. syringae*]	gnl|PRJNA278487|VT47_05295		2.358643118	up	gi|489410942|ref|WP_003316920.1|	polar amino acid ABC transporter, inner membrane
gnl|PRJNA278487|VT47_14840	infA	-2.211945799	down	gi|15597815|ref|NP_251309.1|	translation initiation factor IF-1 [*P. aeruginosa* PAO1]	gnl|PRJNA278487|VT47_16620		2.347979065	up	gi|489522799|ref|WP_003427585.1|	hypothetical protein [*P. syringae*]
gnl|PRJNA278487|VT47_01435	scoP	-2.265708312	down	gi|489521197|ref|WP_003425988.1|	electron transport protein SCO1/SenC [*P. syringae*]	gnl|PRJNA278487|VT47_16425	flgF	2.341675014	up	gi|66046705|ref|YP_236546.1|	flagellar basal body rod protein FlgF [*P. syringae* pv. *syringae*
gnl|PRJNA278487|VT47_06455	ybjK	-2.286176546	down	gi|489522548|ref|WP_003427334.1|	hypothetical protein [*P. syringae*]	gnl|PRJNA278487|VT47_14740		2.326368289	up	gi|489411981|ref|WP_003317936.1|	serralysin [*Pseudomonas* serralysin] [*P. syringae* pv.
gnl|PRJNA278487|VT47_16165		-2.286176546	down	gi|489516387|ref|WP_003421217.1|	hypothetical protein [*P. syringae*]	gnl|PRJNA278487|VT47_22975		2.322686481	up	gi|489522237|ref|WP_003427025.1|	NADH:flavin oxidoreductase [*P. syringae*]
gnl|PRJNA278487|VT47_01585		-2.303468399	down	gi|66043585|ref|YP_233426.1|	hypothetical protein Psyr_0316 [*P. syringae* pv. *syringae* B728a]	gnl|PRJNA278487|VT47_09695	kdpB	2.279173986	up	gi|489518829|ref|WP_003423634.1|	potassium-transporting ATPase subunit B [*P. syringae*]
gnl|PRJNA278487|VT47_00795		-2.338060487	down	gi|495366359|ref|WP_008091073.1|	C4-dicarboxylate ABC transporter [*Pseudomonas* sp. GM84]	gnl|PRJNA278487|VT47_23870		2.267979749	up	gi|66048184|ref|YP_238025.1|	hypothetical protein Psyr_4960 [*P. syringae* pv. *syringae*
gnl|PRJNA278487|VT47_00345		-2.338060487	down	gi|489412737|ref|WP_003318679.1|	hypothetical protein [*P. syringae*]	gnl|PRJNA278487|VT47_16235	flhF	2.252385212	up	gi|489517355|ref|WP_003422173.1|	flagellar biosynthesis regulator FlhF [*P. syringae*]
gnl|PRJNA278487|VT47_10345		-2.338060487	down	gi|489409575|ref|WP_003315568.1|	hypothetical protein [*P. syringae*]	gnl|PRJNA278487|VT47_20965		2.237735212	up	gi|489478981|ref|WP_003384028.1|	DedA:phosphoesterase, PA-phosphatase related protein,
gnl|PRJNA278487|VT47_22755	betI	-2.397545675	down	gi|66047958|ref|YP_237799.1|	transcriptional regulator BetI [*P. syringae* pv. *syringae* B728a]	gnl|PRJNA278487|VT47_11740		2.2322548	up	gi|489511592|ref|WP_003416443.1|	sugar ABC transporter substrate-binding protein [*P.*
gnl|PRJNA278487|VT47_04670		-2.46017758	down	gi|498112884|ref|WP_010427040.1|	6-carboxy-5,6,7,8-tetrahydropterin synthase [*P. syringae*]	gnl|PRJNA278487|VT47_16445	flgD	2.2213032	up	gi|489410799|ref|WP_003316779.1|	flagellar basal body rod modification protein [*P.*

gnl|PRJNA278487|VT47_16930		-2.547265713	down	gi|489520008|ref|WP_003424806.1|	hypothetical protein [*P. syringae*]
gnl|PRJNA278487|VT47_05675		-2.562057064	down	gi|489517011|ref|WP_003421835.1|	hypothetical protein [*P. syringae*]
gnl|PRJNA278487|VT47_06825	fpr	-2.800403118	down	gi|66044635|ref|YP_234476.1|	ferredoxin--NADP(+) reductase [*P. syringae* pv. *syringae* B728a]
gnl|PRJNA278487|VT47_23465	ssuA	-2.831514635	down	gi|489436680|ref|WP_003342194.1|	ABC transporter, substrate-binding protein, aliphatic sulfonate
gnl|PRJNA278487|VT47_14685		-3.608653618	down	gi|489411967|ref|WP_003317922.1|	hypothetical protein [*P. syringae*]
gnl|PRJNA278487|VT47_06210		-4.370026406	down	gi|489408205|ref|WP_003314199.1|	2-nitropropane dioxygenase NPD [*P. syringae*]
gnl|PRJNA278487|VT47_06205	hmp	-5.273877345	down	gi|489408203|ref|WP_003314198.1|	bifunctional nitric oxide dioxygenase/dihydropteridine
gnl|PRJNA278487|VT47_13480	YurZ	-5.438501985	down	gi|489521596|ref|WP_003426385.1|	4-carboxymuconolactone decarboxylase [*P. syringae*]
gnl|PRJNA278487|VT47_03005	livG	2.217972815	up	gi|489411251|ref|WP_003317225.1|	amino acid ABC transporter ATP-binding protein [*P.*
gnl|PRJNA278487|VT47_08210		2.211548498	up	gi|489504844|ref|WP_003409726.1|	ABC transporter substrate-binding protein [*P. syringae*]
gnl|PRJNA278487|VT47_19370	LysE	2.197066539	up	gi|489407490|ref|WP_003313496.1|	amino acid transporter LysE [*P. syringae*]
gnl|PRJNA278487|VT47_13395	xylF	2.196261586	up	gi|498091035|ref|WP_010405191.1|	periplasmic binding protein/LacI transcriptional
gnl|PRJNA278487|VT47_00525		2.195070896	up	gi|489532526|ref|WP_003437257.1|	1-phosphofructokinase B [*P. syringae*]
gnl|PRJNA278487|VT47_08515		2.193129829	up	gi|498095086|ref|WP_010409242.1|	hypothetical protein [*P. syringae*]
gnl|PRJNA278487|VT47_15205	phaC	2.193129829	up	gi|71735738|ref|YP_275336.1|	monovalent cation/H+ antiporter subunit C [*P.*
gnl|PRJNA278487|VT47_12290		2.193129829	up	gi|489490523|ref|WP_003395474.1|	sugar ABC transporter substrate-binding protein [*P.*
gnl|PRJNA278487|VT47_09405	IclR	2.193129829	up	gi|489489562|ref|WP_003394515.1|	regulatory protein, IclR [*P. syringae*]
gnl|PRJNA278487|VT47_15125		2.191336051	up	gi|66046467|ref|YP_236308.1|	3-oxoacid CoA-transferase [*P. syringae* pv. *syringae* B728a]
gnl|PRJNA278487|VT47_11155		2.189402697	up	gi|66045570|ref|YP_235411.1|	binding-protein dependent transport system inner
gnl|PRJNA278487|VT47_05555		2.189402697	up	gi|489408071|ref|WP_003314067.1|	fimbrial protein [*Pseudomonas* fimbrial protein] [*P. syringae*
gnl|PRJNA278487|VT47_19825	ptsH	2.189402697	up	gi|66047378|ref|YP_237219.1|	phosphocarrier HPr protein [*P. syringae* pv. *syringae* B728a]
gnl|PRJNA278487|VT47_16440	flgE	2.182896778	up	gi|489517331|ref|WP_003422150.1|	flagellar hook protein FlgE [*P. syringae*]
gnl|PRJNA278487|VT47_21430		2.179323696	up	gi|489523003|ref|WP_003427788.1|	hypothetical protein, partial [*P. syringae*]
gnl|PRJNA278487|VT47_11540		2.179323696	up	gi|489412044|ref|WP_003317997.1|	binding-protein dependent transport system inner
gnl|PRJNA278487|VT47_05405	gcvH	2.173726838	up	gi|489410914|ref|WP_003316893.1|	glycine cleavage system protein H [*P. syringae*]
gnl|PRJNA278487|VT47_16375	fliC	2.156231928	up	gi|489410818|ref|WP_003316798.1|	flagellin [*P. syringae*]
gnl|PRJNA278487|VT47_15455	manC	2.148167354	up	gi|490552069|ref|WP_004417179.1|	Mannose-1-phosphate guanylyltransferase/mannose-6-
gnl|PRJNA278487|VT47_23865		2.137340713	up	gi|489412862|ref|WP_003318802.1|	hypothetical protein [*P. syringae*]
gnl|PRJNA278487|VT47_08150	HlyD	2.134989778	up	gi|489397773|ref|WP_003304251.1|	secretion protein HlyD [*P. syringae*]
gnl|PRJNA278487|VT47_17640		2.128840792	up	gi|514421557|ref|WP_016568720.1|	Hypothetical protein [*P. syringae*]
gnl|PRJNA278487|VT47_21795	rplW	2.120088455	up	gi|28867856|ref|NP_790475.1|	50S ribosomal protein L23 [*P. syringae* pv. *tomato* str.
gnl|PRJNA278487|VT47_13140		2.103592025	up	gi|514421089|ref|WP_016568252.1|	Putative 2,3-diaminopropionate biosynthesis protein SbnAB

gnl|PRJNA278487|VT47_1 8785		2.103592025	up	gi|489520533|ref|WP_00342532 8.1|	major facilitator transporter [*P. syringae*]
gnl|PRJNA278487|VT47_0 7535		2.103592025	up	gi|514420383|ref|WP_01656754 6.1|	Putative AP superfamily protein [*P. syringae*]
gnl|PRJNA278487|VT47_0 5190		2.093964858	up	gi|498110448|ref|WP_01042460 4.1|	mannose-1-phosphate guanylyltransferase/mannose-6-
gnl|PRJNA278487|VT47_0 3010	livF	2.075429382	up	gi|489411252|ref|WP_00331722 6.1|	amino acid ABC transporter ATPase [*P. syringae*]
gnl|PRJNA278487|VT47_1 8645	gltI	2.02100614	up	gi|71735103|ref|YP_276039.1|	amino acid ABC transporter substrate-binding protein [*P.*
gnl|PRJNA278487|VT47_0 6900	oprD	2.020795717	up	gi|489517279|ref|WP_00342210 0.1|	outer membrane porin [*P. syringae*]
gnl|PRJNA278487|VT47_0 9985	prpB	2.014645267	up	gi|489511285|ref|WP_00341613 7.1|	2-methylisocitrate lyase [*P. syringae*]
gnl|PRJNA278487|VT47_1 2325		2.012352795	up	gi|489515749|ref|WP_00342058 3.1|	amino acid adenylation [*P. syringae*]
gnl|PRJNA278487|VT47_1 9940		2.012352795	up	gi|489435061|ref|WP_00334059 4.1|	hypothetical protein [*P. syringae*]
gnl|PRJNA278487|VT47_2 4210		2.011170247	up	gi|498108541|ref|WP_01042269 7.1|	response regulator receiver, partial [*P. syringae*]
gnl|PRJNA278487|VT47_1 8670	dctD	2.010173482	up	gi|489524057|ref|WP_00342883 3.1|	helix-turn-helix, Fis-type [*P. syringae*]
gnl|PRJNA278487|VT47_1 9930		-2.004339677	down	gi|514421756|ref|WP_01656891 9.1|	Putative SAM-dependent methyltransferase [*P. syringae*]
gnl|PRJNA278487|VT47_1 0035		-2.008528289	down	gi|66045335|ref|YP_235176.1|	hypothetical protein Psyr_2095 [*P. syringae* pv. *syringae*
gnl|PRJNA278487|VT47_0 1960		-2.008817855	down	gi|489410658|ref|WP_00331664 0.1|	1-(5-phosphoribosyl)-5-[(5-phosphoribosylamino)methylid
gnl|PRJNA278487|VT47_0 6065	iscR	-2.059056775	down	gi|489504227|ref|WP_00340911 1.1|	transcriptional regulator [*P. syringae*]
gnl|PRJNA278487|VT47_1 7990		-2.063599105	down	gi|489488315|ref|WP_00339327 1.1|	Alpha/beta hydrolase fold protein [*P. syringae*]
gnl|PRJNA278487|VT47_1 8535		-2.094305238	down	gi|378947966|ref|YP_00520545 4.1|	protein TnpC1 [*P. fluorescens* F113]
gnl|PRJNA278487|VT47_1 4305		-2.101218674	down	gi|489497009|ref|WP_00340192 3.1|	hypothetical protein [*P. syringae*]
gnl|PRJNA278487|VT47_0 1435	scoP	-2.101218674	down	gi|489521197|ref|WP_00342598 8.1|	electron transport protein SCO1/SenC [*P. syringae*]
gnl|PRJNA278487|VT47_1 9980	rimP	-2.110951985	down	gi|66047409|ref|YP_237250.1|	hypothetical protein Psyr_4182 [*P. syringae* pv. *syringae*
gnl|PRJNA278487|VT47_1 8865		-2.117683001	down	gi|489479157|ref|WP_00338419 6.1|	hypothetical protein [*P. syringae*]
gnl|PRJNA278487|VT47_1 9750	IbaG	-2.122805745	down	gi|66047363|ref|YP_237204.1|	BolA-like protein [*P. syringae* pv. *syringae* B728a]
gnl|PRJNA278487|VT47_1 9855		-2.130144597	down	gi|489435089|ref|WP_00334062 2.1|	Nitrilase/cyanide hydratase and apolipoprotein *N* -
gnl|PRJNA278487|VT47_2 2480		-2.130641011	down	gi|28867736|ref|NP_790355.1|	hypothetical protein PSPTO_0507 [*P. syringae* pv.

gnl|PRJNA278487|VT47_06285		-2.147421739	down	gi|489434135|ref|WP_003339676.1|	hypothetical protein [*P. syringae*]
gnl|PRJNA278487|VT47_05580		-2.147421739	down	gi|489510436|ref|WP_003415292.1|	hypothetical protein [*P. syringae*]
gnl|PRJNA278487|VT47_13505	pgsA	-2.162371081	down	gi|489505974|ref|WP_003410850.1|	CDP-diacylglycerol--glycerol-3-phosphate 3-
gnl|PRJNA278487|VT47_09110		-2.203878899	down	gi|489440989|ref|WP_003346461.1|	hypothetical protein [*P. syringae*]
gnl|PRJNA278487|VT47_18525	capB	-2.276417664	down	gi|28871287|ref|NP_793906.1|	cold shock protein CapB [*P. syringae* pv. *tomato* str.
gnl|PRJNA278487|VT47_10745	phnD	-2.295314421	down	gi|489519120|ref|WP_003423924.1|	phosphonates ABC transporter periplasmic phosphonates-
gnl|PRJNA278487|VT47_18050		-2.333349261	down	gi|71734880|ref|YP_273722.1|	hypothetical protein PSPPH_1467 [*P. syringae* pv.
gnl|PRJNA278487|VT47_23965		-2.38617042	down	gi|490550126|ref|WP_004415244.1|	Hypothetical protein [*P. syringae*]
gnl|PRJNA278487|VT47_21870	secE	-2.435436528	down	gi|492563235|ref|WP_005887064.1|	preprotein translocase subunit SecE [*P. syringae* group
gnl|PRJNA278487|VT47_20645		-2.442236402	down	gi|489407834|ref|WP_003313834.1|	putative DMT superfamily transporter inner membrane
gnl|PRJNA278487|VT47_04670	queD	-2.463928436	down	gi|498112884|ref|WP_010427040.1|	6-carboxy-5,6,7,8-tetrahydropterin synthase [*P.*
gnl|PRJNA278487|VT47_24285	aspA	-2.568911155	down	gi|489410088|ref|WP_003316079.1|	aspartate ammonia-lyase [*P. syringae*]
gnl|PRJNA278487|VT47_04260	phnA	-2.599544569	down	gi|498110793|ref|WP_010424949.1|	PhnA protein [*P. syringae*]
gnl|PRJNA278487|VT47_21470		-2.612913396	down	gi|498103304|ref|WP_010417460.1|	NAD-dependent epimerase/dehydratase, partial
gnl|PRJNA278487|VT47_03545	rpsT	-2.736658418	down	gi|488615127|ref|WP_002551976.1|	30S ribosomal protein S20 [*P. savastanoi*]
gnl|PRJNA278487|VT47_18590	bfr	-2.81758897	down	gi|28871302|ref|NP_793921.1|	bacterioferritin [*P. syringae* pv. *tomato* str. DC3000]
gnl|PRJNA278487|VT47_21040		-2.950294637	down	gi|66047613|ref|YP_237454.1|	hypothetical protein Psyr_4386 [*P. syringae* pv. *syringae*
gnl|PRJNA278487|VT47_10030		-3.038711997	down	gi|489478593|ref|WP_003383654.1|	crfX protein [*Pseudomonas* crfX protein] [*P. syringae* pv.
gnl|PRJNA278487|VT47_13300		-3.05804175	down	gi|490803134|ref|WP_004665266.1|	hypothetical protein [*P. amygdali*]
gnl|PRJNA278487|VT47_21930	erpA	-3.094401765	down	gi|28867833|ref|NP_790452.1|	HesB/YadR/YfhF family protein [*P. syringae* pv.
gnl|PRJNA278487|VT47_01265		-3.359794678	down	gi|489521165|ref|WP_003425957.1|	hypothetical protein [*P. syringae*]
gnl|PRJNA278487|VT47_14685		-3.652499976	down	gi|489411967|ref|WP_003317922.1|	hypothetical protein [*P. syringae*]
