# Supplementary material for: Identification of the Genes of the Plant Pathogen Pseudomonas syringae MB03 Required for the Nematicidal Activity Against Caenorhabditis elegans Through an Integrated Approach
Source: Front Microbiol. 2022 Mar 9;13:826962. doi: 10.3389/fmicb.2022.826962 (PMC8959697; doi:10.3389/fmicb.2022.826962)
Supplement: Supplementary file 6 [file Data_Sheet_10.PDF]

Table S6. Transcriptional response of selected genes present on predicted genomic islands.

Locus tag	Expression [a]		Product	PS core [b]
	12 h	24 h		
VT47_06205	Down		bifunctional nitric oxide dioxygenase	Absent
VT47_06210	Down		nitronate monooxygenase	Absent
VT47_06215	Down		tautomerase	Absent
VT47_22320		Up	conjugal transfer protein	Absent
VT47_23965		Down	Hypothetical protein	Absent

Note: [a] Differential expression was investigated during two growth phases of bacterial pathogen, exponential phase (12 h) and stationary phase (24 h)

[b] Comparison was done with the core genome of *P. syringae* (PS core) determined by Baltrus and co-workers [1]

References

[1] Baltrus DA, Nishimura MT, Romanchuk A, Chang JH, Mukhtar MS, Cherkis K, et al. Dynamic evolution of pathogenicity revealed by sequencing and comparative genomics of 19 *Pseudomonas syringae* isolates. PLoS Pathog. 2011;7: e1002132.
