# Supplementary material for: Identification of the Genes of the Plant Pathogen Pseudomonas syringae MB03 Required for the Nematicidal Activity Against Caenorhabditis elegans Through an Integrated Approach
Source: Front Microbiol. 2022 Mar 9;13:826962. doi: 10.3389/fmicb.2022.826962 (PMC8959697; doi:10.3389/fmicb.2022.826962)
Supplement: Supplementary file 7 [file Data_Sheet_5.PDF]

Table S7. Distribution of type III secretion system effector proteins in *P. syringae* MB03.

TTE gene	Locus tag	Strain showing best BLASTp results according to PPI database [a]	Percent similarity	
			***P. syringae* pv. *syringae* B64** [b]	***P. syringae* pv. *syringae* SM** [b]
avrE1	VT47_05850	*P. syringae.* pv *syringae* B728a	99	99
hopI1	VT47_20735	*P. syringae* pv. syringae B728a	96	97
hopM1	VT47_05840	*P. syringae* pv. *syringae* B728a	99	98
hopZ3	VT47_06000	*P. syringae* pv. *syringae* B728a	100	Not found
hopAA1	VT47_05825	*P. syringae* pv. *maculicola* ES4326	98	98
hopAH2	VT47_14550	*P. syringae* pv. *syringae* B728a	99	99
hopAK1	VT47_18270	*P. syringae* pv. *syringae* B728a	97	96
		P. syringae pv. *syringae* FF5	Not found	99
hopAZ1	VT47_08660	*P. syringae* pv. *aptata* DSM50252		
hopBA1	VT47_12675	*P. syringae* pv. *aptata* DSM50252	99	98
hopBC1	VT47_07955	*P. syringae* pv. *aptata* DSM50252	Not found	Not found

Note: [a] Sequences of Hop proteins were obtained from www.pseudomonas-syringae.org. The column shows best blastp results of PPI database.

[b] Protein sequences of bacterial strains were obtained from NCBI and used to find out homolog of MB03 effectors.
