# Supplementary material for: Identification of the Genes of the Plant Pathogen Pseudomonas syringae MB03 Required for the Nematicidal Activity Against Caenorhabditis elegans Through an Integrated Approach
Source: Front Microbiol. 2022 Mar 9;13:826962. doi: 10.3389/fmicb.2022.826962 (PMC8959697; doi:10.3389/fmicb.2022.826962)
Supplement: Supplementary file 8 [file Data_Sheet_6.PDF]

Table S8. Homologs of nematicidal genes of *P. aeruginosa* PAO1 and PA14 found in *P. syringae* MB03

No	Gene in PAO1	PAO1/PA14 locus tag	PS Core	Length (aa)	MB03 locus tag	H value	Function	PA14*
***P. aeruginosa* PAO1**								
1	*ptsP*	PA0337	Present	759	VT47_23320	0.859	phosphoenolpyruvate-protein phosphotransferase	Present
2	*hlyD*	PA0425	Present	383	VT47_19135	0.656	Resistance-Nodulation-Cell Division (RND) multidrug efflux membrane fusion protein MexA precursor	Absent
3	*pdxA*	PA0593	Present	328	VT47_22200	0.802	pyridoxal phosphate biosynthetic protein PdxA	Absent
4	*xdhB*	PA1523	Present	799	VT47_08595	0.813	xanthine dehydrogenase	Absent
5	*kdpB*	PA1634	Present	690	VT47_09695	0.835	potassium-transporting ATPase, B chain	Absent
6		PA2854	Present	323	VT47_09910	0.728	conserved hypothetical protein	Absent
7		PA3071	Present	312	VT47_08100	0.689	hypothetical protein	Absent
8		PA3139	Present	398	VT47_09305	0.771	probable amino acid aminotransferase	Absent
9	*prc*	PA3257	Present	698	VT47_07565	0.754	periplasmic tail-specific protease	Absent
10		PA3448	Present	274	VT47_12125	0.65	probable permease of ABC transporter	Absent
11		PA3449	Present	333	VT47_12130	0.64	conserved hypothetical protein	Absent
12		PA3460	Present	585	VT47_17860	0.73	probable acetyltransferase	Absent
13	*rpoS*	PA3622	Present	334	VT47_06765	0.884	sigma factor RpoS	Absent
14	*mucP*	PA3649	Present	450	VT47_06645	0.762	conserved hypothetical protein	Absent
15		PA3799	Present	493	VT47_06145	0.805	conserved hypothetical protein	Absent
16		PA3950	Present	449	VT47_20640	0.682	probable ATP-dependent RNA helicase	Absent
17	*rlpA*	PA4000	Present	342	VT47_20920	0.669	hypothetical protein	Absent
18		PA4113	Present	396	VT47_04270	0.644	probable major facilitator superfamily (MFS) transporter	Absent
19	*tufA*	PA4265	Present	397	VT47_21815	0.869	elongation factor Tu	Absent

20	*hisD*	PA4448	Present	440	VT47_19735	0.886	histidinol dehydrogenase	Absent
21	*pilD*	PA4528	Present	290	VT47_03925	0.79	type 4 prepilin peptidase PilD	Absent
22		PA4684	Present	432	VT47_04220	0.815	hypothetical protein	Absent
23		PA4767	Present	144	VT47_20085	0.743	conserved hypothetical protein	Absent
24	*smpB*	PA4768	Present	159	VT47_20095	0.812	SmpB protein	Absent
25		PA5138	Present	250	VT47_23530	0.668	hypothetical protein	Absent
26		PA5202	Present	129	VT47_01295	0.643	hypothetical protein	Absent
27	*gshA*	PA5203	Present	527	VT47_01290	0.695	glutamate-cysteine ligase	Present
28	*crc*	PA5332	Present	259	VT47_01090	0.865	catabolite repression control protein	Absent
29	*phoB*	PA5360	Present	229	VT47_24190	0.943	two-component response regulator PhoB	Absent
30	*phoR*	PA5361	Present	443	VT47_24195	0.752	two-component sensor PhoR	Absent
***P. syringae* PA14**								
1	*ptsP*	PA14_04410	Present	759	VT47_23320	0.859	phosphoenolpyruvate-protein phosphotransferase	
2	*aruB*	PA14_52660	Present	448	VT47_16870	0.79	succinylarginine dihydrolase	
3	*pepP*	PA14_69000	Present	444	VT47_01610	0.815	aminopeptidase P	
4	*cspB*	PA14_05960	Present	69	VT47_05390	0.739	putative major cold shock protein	
5	*fabF1*	PA14_25690	Present	414	VT47_07740	0.836	beta-ketoacyl-acyl carrier protein synthase II	
6	*gacA*	PA14_30650	Present	214	VT47_13515	0.897	response regulator GacA	
7	*glnK*	PA14_69810	Present	112	VT47_00880	0.973	Nitrogen regulatory protein PII	
8	*surA,*	PA14_07760	Present	430	VT47_22195	0.735	peptidyl-prolyl cis-trans isomerase	
9	*minD*	PA14_22020	Present	270	VT47_07500	0.885	cell division inhibitor MinD	
10	*clpA*	PA14_30230	Present	758	VT47_14845	0.904	ATP-dependent clp protease, ATP-binding subunit ClpA	
11	*gnyD*	PA14_38440	Present	387	VT47_11890	0.868	Citronelloyl-CoA dehydrogenase, GnyD	
12	*fleN*	PA14_45640	Present	280	VT47_16230	0.893	flagellar synthesis regulator FleN	
13	*lysC*	PA14_52580	Present	412	VT47_16840	0.908	aspartate kinase alpha and beta chain	

14		PA14_52610	Absent	334	VT47_16850	0.79	possible threonine aldolase
15	*prpC*	PA14_53950	Present	375	VT47_09990	0.853	citrate synthase 2
16		PA14_41730	Present	327	VT47_09965	0.826	conserved hypothetical protein
17	*gacS/lemA*	PA14_52260	Absent	925	VT47_17580	0.672	sensor/response regulator hybrid
18		PA14_61680	Present	276	VT47_04675	0.704	methyl transferase
19	*nusA*	PA14_62770	Present	493	VT47_19975	0.876	transcription elongation factor
20	*apaH*	PA14_07700	Present	283	VT47_22215	0.723	diadenosine tetraphosphatase
21	*nagZ*	PA14_25195	Absent	332	VT47_15350	0.843	beta-hexosaminidase
22	*clpS*	PA14_30210	Present	122	VT47_14850	0.803	ATP-dependent Clp protease adaptor protein
23		PA14_31580	Present	409	VT47_11915	0.868	acyl-CoA dehydrogenase
24		PA14_66120	Absent	318	VT47_02665	0.752	hypothetical protein
25	*prpB*	PA14_53940	Present	298	VT47_09985	0.862	carboxyphosphonoenolpyruvate phosphonomutase
26	*dnaK*	PA14_62970	Present	637	VT47_20055	0.876	molecular chaperone
27	*miaA*	PA14_65320	Present	323	VT47_02865	0.758	delta 2-isopentenylpyrophosphate transferase
28	*cysQ*	PA14_68370	Present	273	VT47_23770	0.67	3'(2'),5'-bisphosphate nucleotidase
29	*gshA*	PA14_68730	Present	527	VT47_01290	0.697	glutamate--cysteine ligase
30	*sltB1*	PA14_12080	Present	340	VT47_20925	0.738	soluble lytic transglycosylase B
31	*galU*	PA14_38350	Present	279	VT47_13815	0.846	UTP-glucose-1-phosphate uridylyltransferase
32		PA14_41710	Present	508	VT47_09970	0.813	putative membrain protein
33	*mucD*	PA14_54390	Present	474	VT47_18880	0.753	serine protease MucD precursor
34	*yhdG*	PA14_64180	Present	332	VT47_21130	0.801	putative tRNA-dihydrouridine synthase
35	*cmpX*	PA14_41590	Present	274	VT47_10035	0.807	cytoplasmic membrane protein
36	*rbfA*	PA14_62740	Present	129	VT47_19965	0.736	ribosome-binding factor A
37	*uvrC*	PA14_30660	Present	608	VT47_13510	0.829	excinuclease ABC subunit C
38		PA14_49930	Absent	279	VT47_18045	0.735	hypothetical protein

39	*tolQ*	PA14_51750	Present	231	VT47_06960	0.909	TolQ protein
40	*typA*	PA14_67560	Present	605	VT47_23145	0.803	GTP-binding protein TypA/BipA
41	*aldH*	PA14_70140	Present	497	VT47_01155	0.863	putative aldehyde dehydrogenase
42	*gshB*	PA14_05310	Present	317	VT47_02435	0.808	glutathione synthetase
43		PA14_06400	Present	308	VT47_13020	0.698	putative transcriptional regulator, LysR family
44	*gnyA*	PA14_38480	Present	655	VT47_11875	0.716	putative acyl-CoA carboxylase alpha chain
45	*tatC*	PA14_66980	Present	267	VT47_01905	0.824	sec-independent protein translocase
46		PA14_25830	Present	189	VT47_07790	0.767	conserved hypothetical protein
47		PA14_27230	Present	151	VT47_17210	0.676	putative transcriptional regulator, MarR family
48	*ppsA*	PA14_41670	Present	791	VT47_10010	0.909	phosphoenolpyruvate synthase
49	*aruC*	PA14_52720	Present	406	VT47_16890	0.776	N-succinylglutamate 5-semialdehyde dehydrogenase
50	*acsA*	PA14_52800	Present	651	VT47_16925	0.862	acetyl-coenzyme A synthetase
51		PA14_41710	Present	508	VT47_09970	0.813	putative membrain protein
52	*fleQ*	PA14_50220	Present	490	VT47_16350	0.827	transcriptional regulator
53	*fixR*	PA14_68040	Absent	245	VT47_18190	0.8	putative short-chain alcohol dehydrogenase
54		PA14_69250	Present	196	VT47_01470	0.755	putative membrane-associated protein
55		PA14_07790	LHV	224	VT47_22180	0.683	putative nucleotidyltransferase
56	*accD/gnyB*	PA14_38460	Present	535	VT47_11885	0.845	acyl-CoA carboxyltransferase beta chain
57	*kdpD*	PA14_43350	Absent	885	VT47_09705	0.753	two-component sensor KdpD
58		PA14_61980	Present	231	VT47_04215	0.78	conserved hypothetical protein
59	*pnp*	PA14_62710	Present	701	VT47_19950	0.86	polyribonucleotide nucleotidyltransferase
60	*vfr*	PA14_08370	Present	214	VT47_21975	0.794	cyclic AMP receptor-like protein
61	*crfX*	PA14_41610	Present	84	VT47_10030	0.655	conserved hypothetical protein
62	*aruG*	PA14_52690	Present	340	VT47_16880	0.85	arginine/ornithine succinyltransferase AII subunit
63		PA14_56790	Present	687	VT47_19380	0.792	putative GGDEF domain/EAL domain protein

64		PA14_57820	Present	79	VT47_19750	0.835	conserved hypothetical protein
65	*ccoN*	PA14_10500	Present	475	VT47_16100	0.787	cytochrome c oxidase subunit
66	*csdA*	PA14_16930	Present	401	VT47_06570	0.661	putative cysteine sulfinate desulfinase
67	*glsA*	PA14_43320	Absent	302	VT47_10970	0.805	putative glutaminase
68		PA14_60800	Present	554	VT47_20470	0.865	putative ABC transporter, ATP-binding protein
69		PA14_69700	Present	233	VT47_00850	0.674	conserved hypothetical protein
70		PA14_07780	Present	338	VT47_22185	0.654	putative phosphotransferase
71	*rhlB*	PA14_14040	Present	507	VT47_05280	0.764	ATP-dependent RNA helicase RhlB
72	*folE1*	PA14_19630	Present	186	VT47_05015	0.866	GTP cyclohydrolase I precursor
73	*topA*	PA14_25110	Present	868	VT47_15380	0.87	DNA topoisomerase I
74		PA14_41140	Present	360	VT47_08285	0.831	putative ABC transporter, permease protein
75		PA14_41150	Present	339	VT47_08280	0.864	putative permease of ABC transporter
76	*pmbA*	PA14_58050	Present	449	VT47_19840	0.811	PmbA protein
77		PA14_11910	Absent	151	VT47_03215	0.748	conserved hypothetical protein
78	*algZ*	PA14_20290	Present	108	VT47_16805	0.796	DNA binding-protein
79	*aruD*	PA14_52670	Absent	488	VT47_16875	0.783	succinylglutamate 5-semialdehyde dehydrogenase
80		PA14_11250	Present	294	VT47_21485	0.782	putative dTDP-4-rhamnose reductase-related protein
81	*iscS*	PA14_14730	Present	404	VT47_06070	0.874	cysteine desulfurase
82	*glpD*	PA14_17930	Present	512	VT47_18640	0.73	glycerol-3-phosphate dehydrogenase
83	*sth*	PA14_25390	Present	464	VT47_09025	0.851	soluble pyridine nucleotide transhydrogenase
84	*atpI*	PA14_73320	Present	126	VT47_24685	0.657	ATP synthase protein I
85	*ompH*	PA14_17170	Present	168	VT47_06655	0.768	putative outer membrane protein OmpH
86	*yadG*	PA14_27770	Present	310	VT47_07640	0.813	putative ABC transporter, ATP-binding protein
87	*secB*	PA14_67720	Present	163	VT47_23490	0.779	secretion protein SecB
