# Supplementary material for: Identification of the Genes of the Plant Pathogen Pseudomonas syringae MB03 Required for the Nematicidal Activity Against Caenorhabditis elegans Through an Integrated Approach
Source: Front Microbiol. 2022 Mar 9;13:826962. doi: 10.3389/fmicb.2022.826962 (PMC8959697; doi:10.3389/fmicb.2022.826962)
Supplement: Supplementary file 9 [file Data_Sheet_7.PDF]

Table S9. ***in silico*** **characterization of selected hypothetical proteins**

Position		Physiochemical Properties				Subcellular Localization			Virulance	Host Pathogen Interaction
Locus	Accession No	Mol Wt.	Isoelectric point	Stability	Aliphatic Index	Signal Pep	TMHMM	CELLO	VICMPred	HPIDB
VT47_06935	KZL40945	26565.64	4.6	stable	80.73	No Signal Seq	No TMH	Cytoplasmic	Non-virulent	Yes
VT47_23445	KZL36278	7627.85	9.22	stable	66.51	No Signal Seq	No TMH	Cytoplasmic	Non-virulent	No
VT47_24345	KZL36140	11333.82	4.33	stable	94.47	No Signal Seq	No TMH	Cytoplasmic	Non-virulent	No
VT47_20505	KZL36988	7578.65	7.79	Satble	43.54	No Signal Seq	No TMH	Periplasmic	Non-virulent	No
VT47_04875	KZL40559.1	50464.01	8.77	Satble	73.71	First 24 AA	1 TMH	Periplasmic	Non-virulent	No
VT47_08995	KZL39868.1	19137.9	5.6	Satble	104.31	First 20 AA	No TMH	Periplasmic	Non-virulent	No
VT47_18175	KZL37697.1	15667.98	6.57	unsatble	84.76	First 22 AA	No TMH	Periplasmic	Non-virulent	No
VT47_11195	KZL39725.1	10790.29	5.69	unsatble	94.3	No Signal Seq	1 TMH	Cytoplasmic	Non-virulent	No
VT47_01705	KZL42270.1	33649.43	9.48	stable	92.38	No Signal Seq	3 TMH	Inner Mem	Non-virulent	No
VT47_21225	KZL36814.1	128833.5	6.02	unsatble	88.16	No Signal Seq	No TMH	Outer Mem	Non-virulent	No
VT47_01585	KZL42246.1	5663.62	9.4	unsatble	103.2	No Signal Seq	1 TMH	Cytoplasmic	Non-virulent	No
VT47_00345	KZL42513.1	6649.55	8.27	unsatble	95.93	No Signal Seq	No TMH	Cytoplasmic	Non-virulent	No
VT47_05675	KZL40709.1	7623.54	8.11	unsatble	49.42	No Signal Seq	No TMH	Cytoplasmic	Non-virulent	No
VT47_17640	KZL37864.1	12282.7	5.68	Satble	89.14	Frist 22 AA	No TMH	Outer Mem	Non-virulent	No
VT47_19940	KZL37514.1	12517.2	9.34	unsatble	77.5	First 30 AA	1 TMH	Outer Mem	Non-virulent	No
VT47_14305	KZL38610.1	17423.09	4.43	stable	115.16	No Signal Seq	No TMH	Cytoplasmic	Non-virulent	No
VT47_18865	KZL37312.1	14366.54	6.72	stable	90.4	first 25 AA	1 TMH	Periplasmic	Non-virulent	No
VT47_05580	KZL40691.1	8062.05	9.77	unsatble	35.81	No Signal Seq	No TMH	Periplasmic	Non-virulent	No
VT47_09110	KZL39890.1	8692.86	4.5	unsatble	111.39	No Signal Seq	No TMH	Cytoplasmic	Non-virulent	No
VT47_23965	KZL36389.1	11957.74	5.01	unsatble	94.65	No Signal Seq	No TMH	Cytoplasmic	Non-virulent	No
VT47_01265	KZL42185.1	12774.61	4.99	stable	100.96	No Signal Seq	No TMH	Cytoplasmic	Non-virulent	No
VT47_14685	KZL38410.1	7709.46	5.03	stable	44.53	First 22 AA	No TMH	Periplasmic	Non-virulent	No
VT47_04220	KZL41203.1	49079.3	6.92	unsatble	97.18	No Signal Seq	No TMH	Cytoplasmic	Non-virulent	No
VT47_01295	KZL42191.1	13733.95	5.92	unsatble	108.19	No Signal Seq	No TMH	Cytoplasmic	Non-virulent	No
VT47_00850	KZL42355.1	27015.93	5.48	stable	102.75	No Signal Seq	No TMH	Cytoplasmic	Non-virulent	No

Captions

Signal Pep = Software used to identify Signal peptide sequence in hypothetical proteins (http://www.cbs.dtu.dk/services/SignalP/)
TMHMM = Software which is used to identify transmembrane helix regions in proteins to find our their presences at membrane. (http://www.cbs.dtu.dk/services/TMHMM/)
CELLO= Software is used to find out subcellular localization of hypothetical protein in cells (http://cello.life.nctu.edu.tw/).
VICMPred = Software which is used to identify to virulence in hypothetical protein. (http://www.imtech.res.in/raghava/vicmpred/)
HPIDB= Host pathogen Interaction Database, used to figure proteins which are involved in host pathogen interaction. (http://www.agbase.msstate.edu/hpi/main.html)

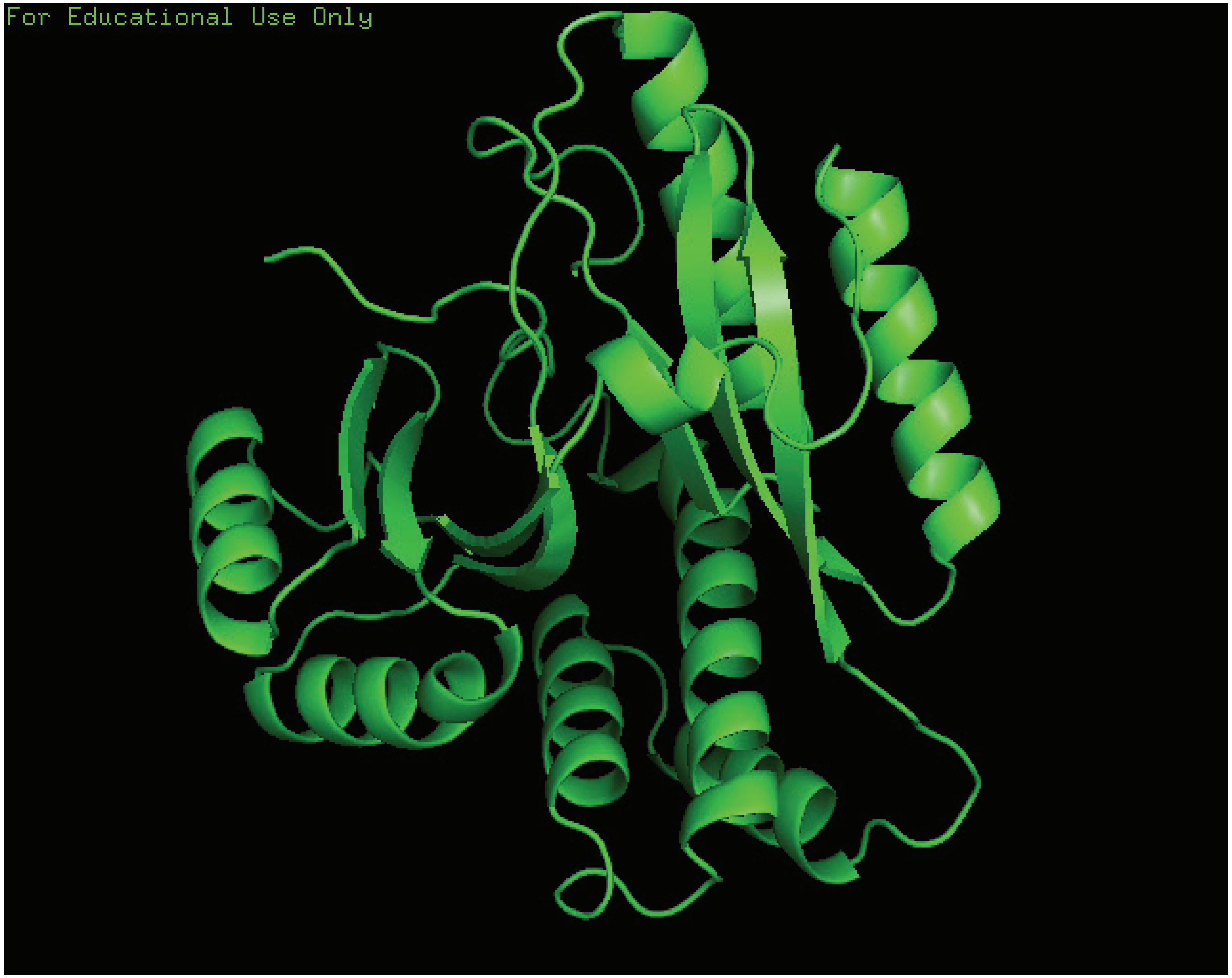

KZL40945.1 hypothetical protein 3D structure predicted by I-TASSER and refined by using 3DRef software

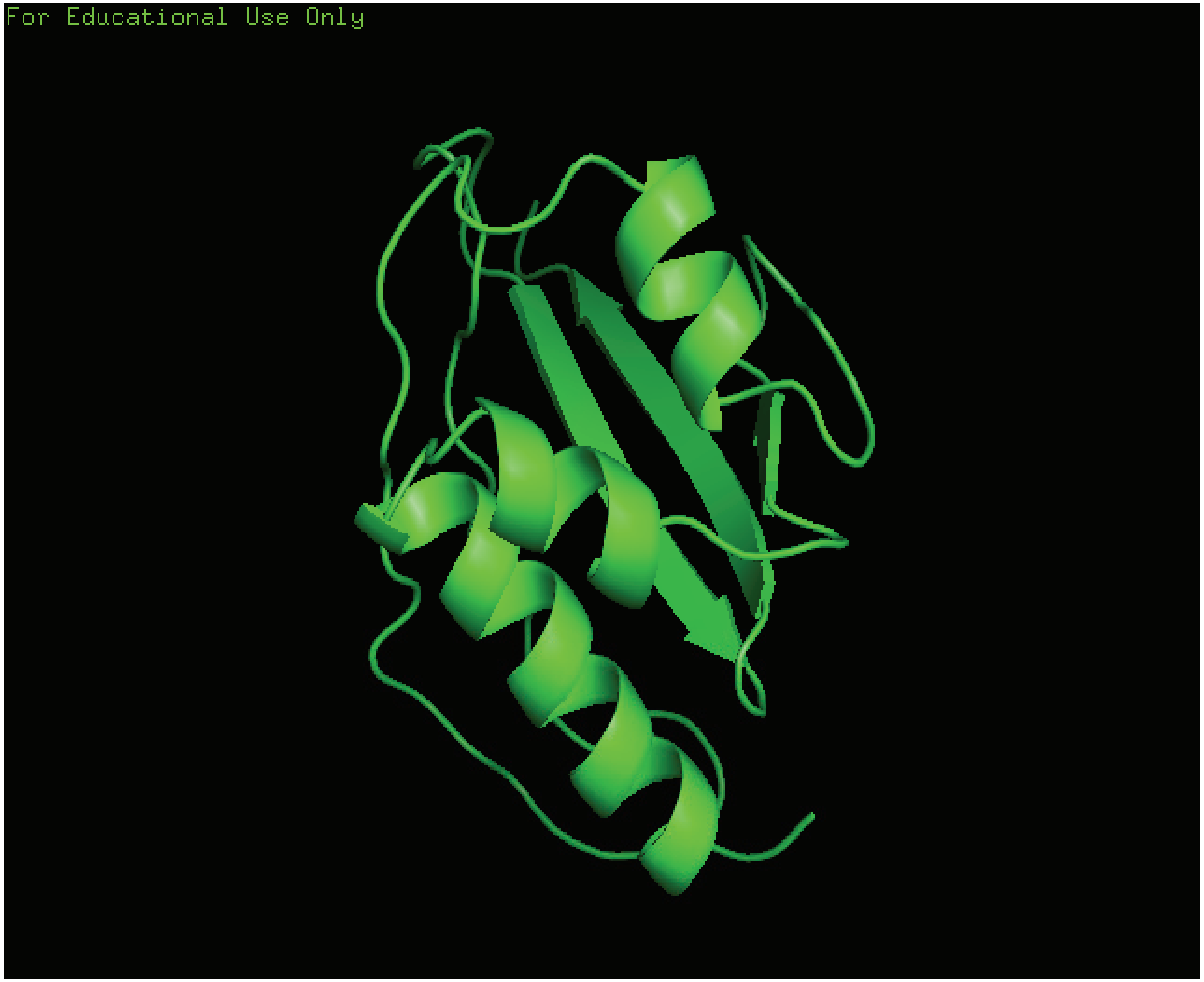

KZL37514 hypothetical protein 3D structure predicted by I-TASSER and refined by using 3DRef software

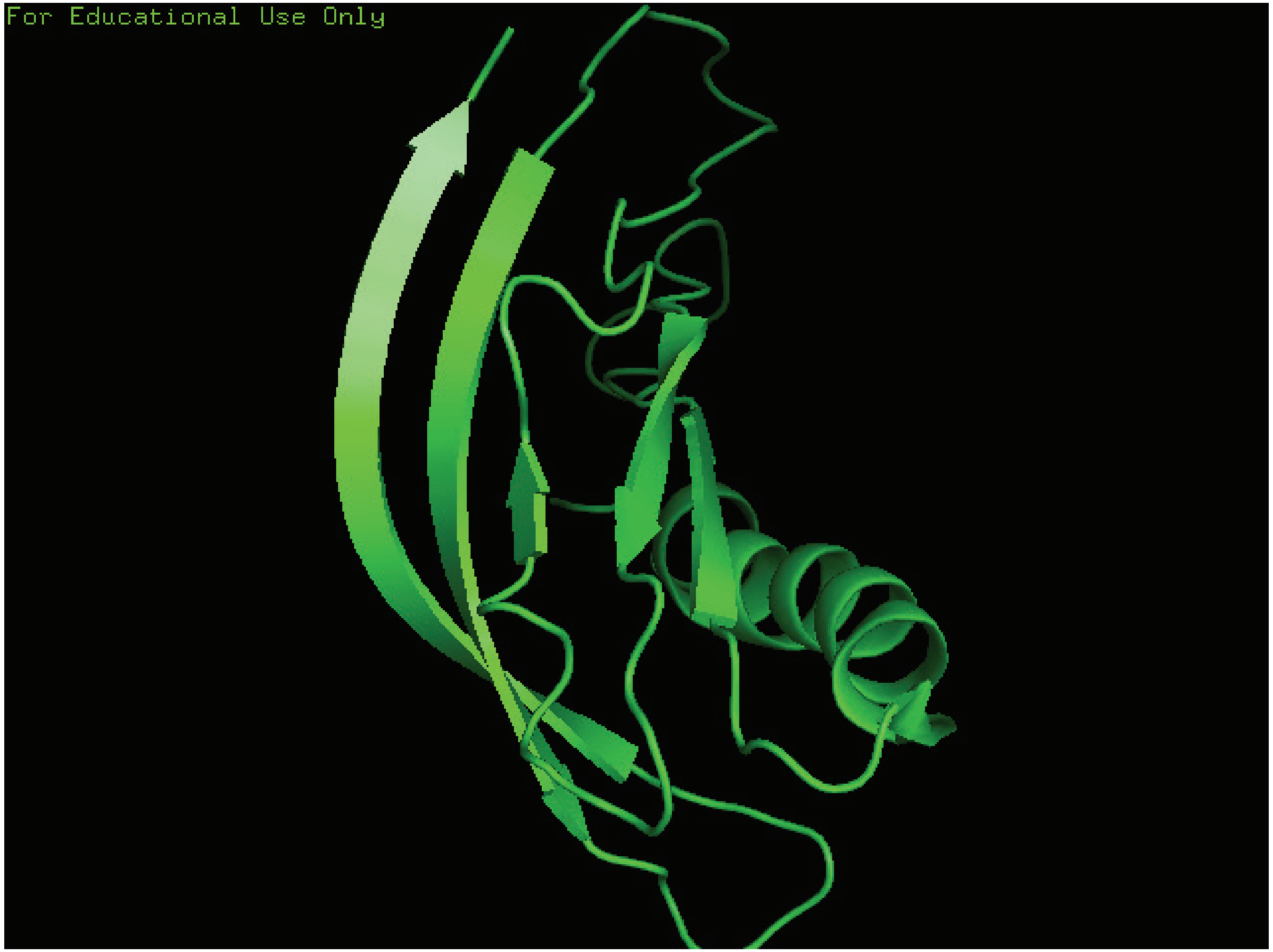

KZL37864 hypothetical protein 3D structure by I-TASSER and refined by using 3DRef software.

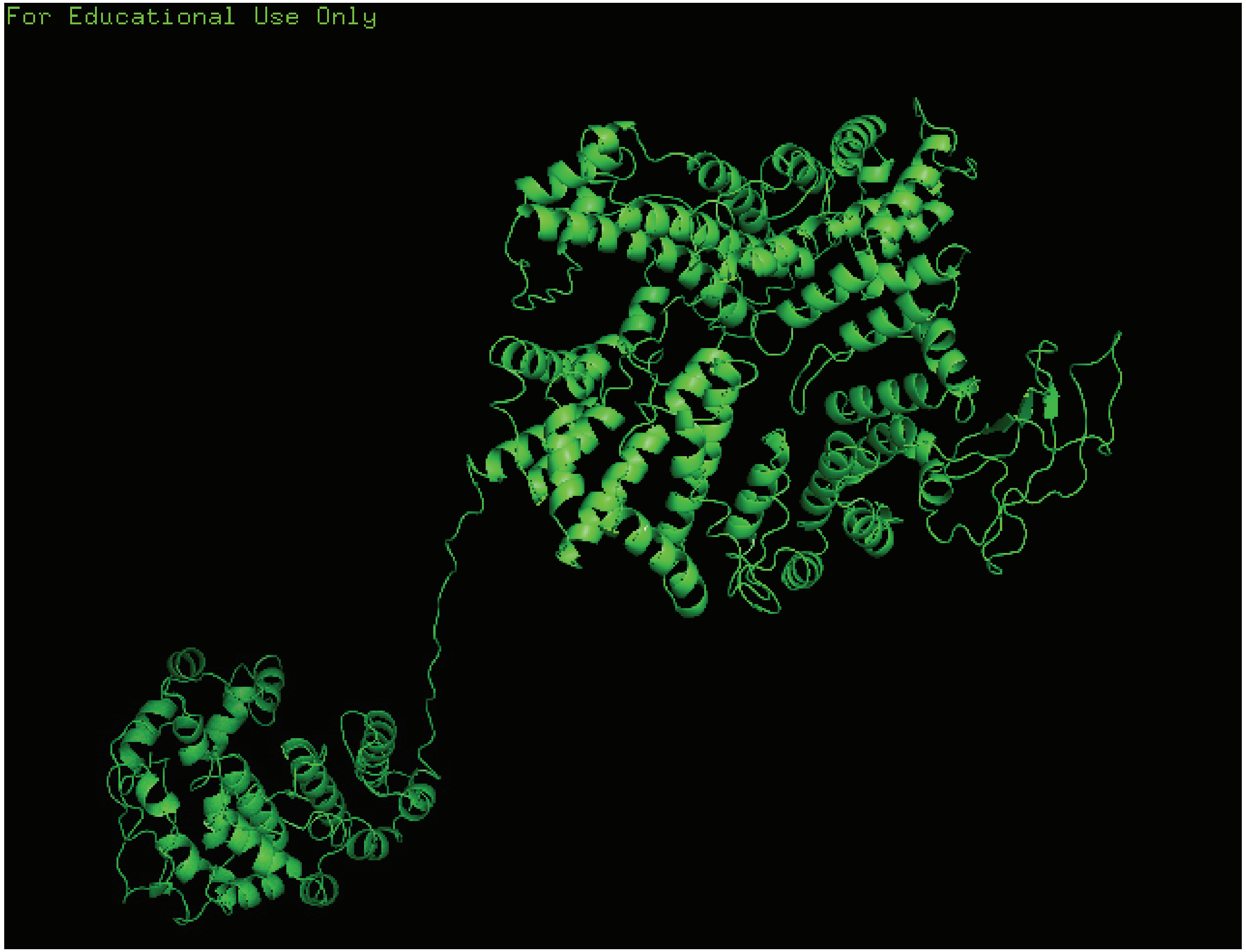

KZL36814 hypothetical protein 3D structure predicted by I-TASSER and refined by using 3DRef software
