# Supplementary material for: Identification of the Genes of the Plant Pathogen Pseudomonas syringae MB03 Required for the Nematicidal Activity Against Caenorhabditis elegans Through an Integrated Approach
Source: Front Microbiol. 2022 Mar 9;13:826962. doi: 10.3389/fmicb.2022.826962 (PMC8959697; doi:10.3389/fmicb.2022.826962)
Supplement: Supplementary file 10 [file Data_Sheet_8.PDF]

Table S10 List of regulated genes from over-expressed transcriptional regulator

Locus Tag of TR	Regulation	TR	Regulated gene	TR Function
VT47_13020	Up	AtzR	*atzD* (cyanuric acid amidohydrolase)	Activator
VT47_09405	Up	PcaR	*pcaI*	Activator
VT47_10490	Up	FadR	*plsB* (glycerol-3-phosphate acyltransferase)	Repressor
			ytfQ (galactofuranose ABC transporter periplasmic binding protein)	Repressor
			araf (L-arabinose ABC transporter periplasmic binding protein)	Activator
			araG (L-arabinose ABC transporter ATPase)	Activator
			araH (L-arabinose ABC transporter permease)	Activator
			araE (arabinose transporter)	Activator
			ygeA (Asp/Glu_racemase family protein)	Activator
VT47_22635	Up	AraC	*araJ* (L-arabinose-inducible putative transporter, MFS family)	Activator
			araB (L-ribulokinase)	Activator
			araA (L-arabinose isomerase)	Activator
			ytfR (putative sugar ABC transporter ATPase)	Activator
			yjfF (putative sugar ABC transporter permease)	Activator
			ytfT (putative sugar ABC transporter permease)	Activator
			ftnB (ferritin B, putative ferrous iron reservoir)	Activator
			yabl (DedA family inner membrane protein)	Activator
