# Supplementary material for: Identification of the Genes of the Plant Pathogen Pseudomonas syringae MB03 Required for the Nematicidal Activity Against Caenorhabditis elegans Through an Integrated Approach
Source: Front Microbiol. 2022 Mar 9;13:826962. doi: 10.3389/fmicb.2022.826962 (PMC8959697; doi:10.3389/fmicb.2022.826962)
Supplement: Supplementary file 11 [file Data_Sheet_11.PDF]

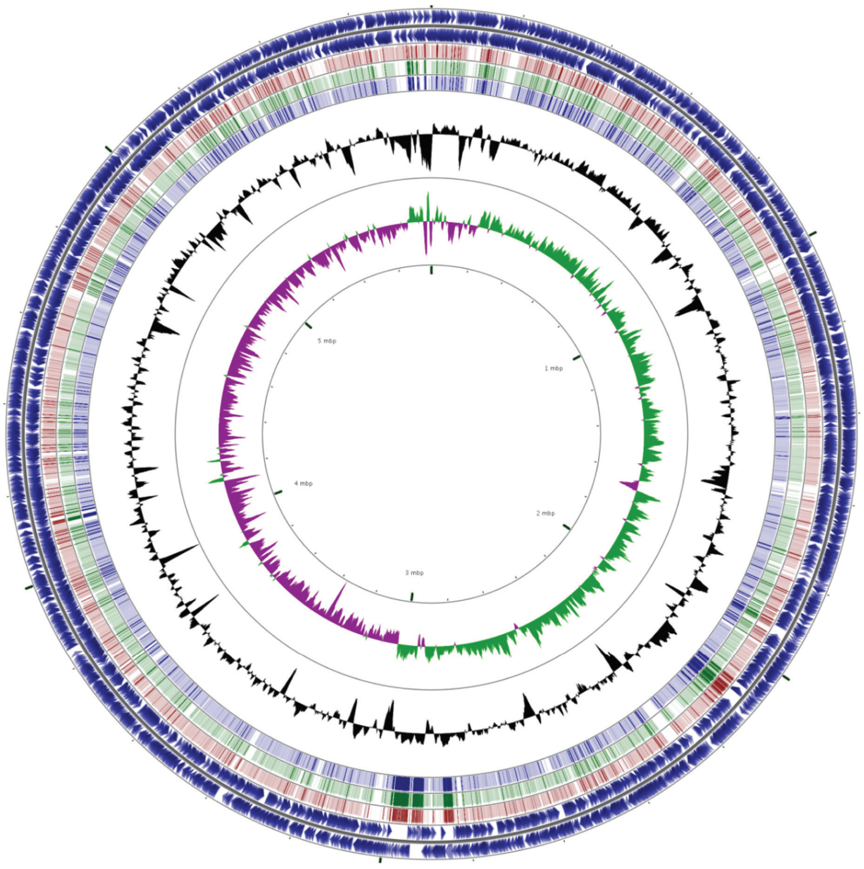

Figure S1. Graphical representation of GC content and GC skew. Concatenated chromosome was used to generate GC content. The outermost two circles show CDS of *P. syringae* MB03. From circle three to five, Blastn comparison with DC3000, CC1557 and B728a are shown, respectively. The sixth circle shows GC content and seventh circle shows GC skew where green color represents positive and purple color shows negative values.
