# Supplementary material for: Identification of the Genes of the Plant Pathogen Pseudomonas syringae MB03 Required for the Nematicidal Activity Against Caenorhabditis elegans Through an Integrated Approach
Source: Front Microbiol. 2022 Mar 9;13:826962. doi: 10.3389/fmicb.2022.826962 (PMC8959697; doi:10.3389/fmicb.2022.826962)
Supplement: Supplementary file 12 [file Data_Sheet_12.PDF]

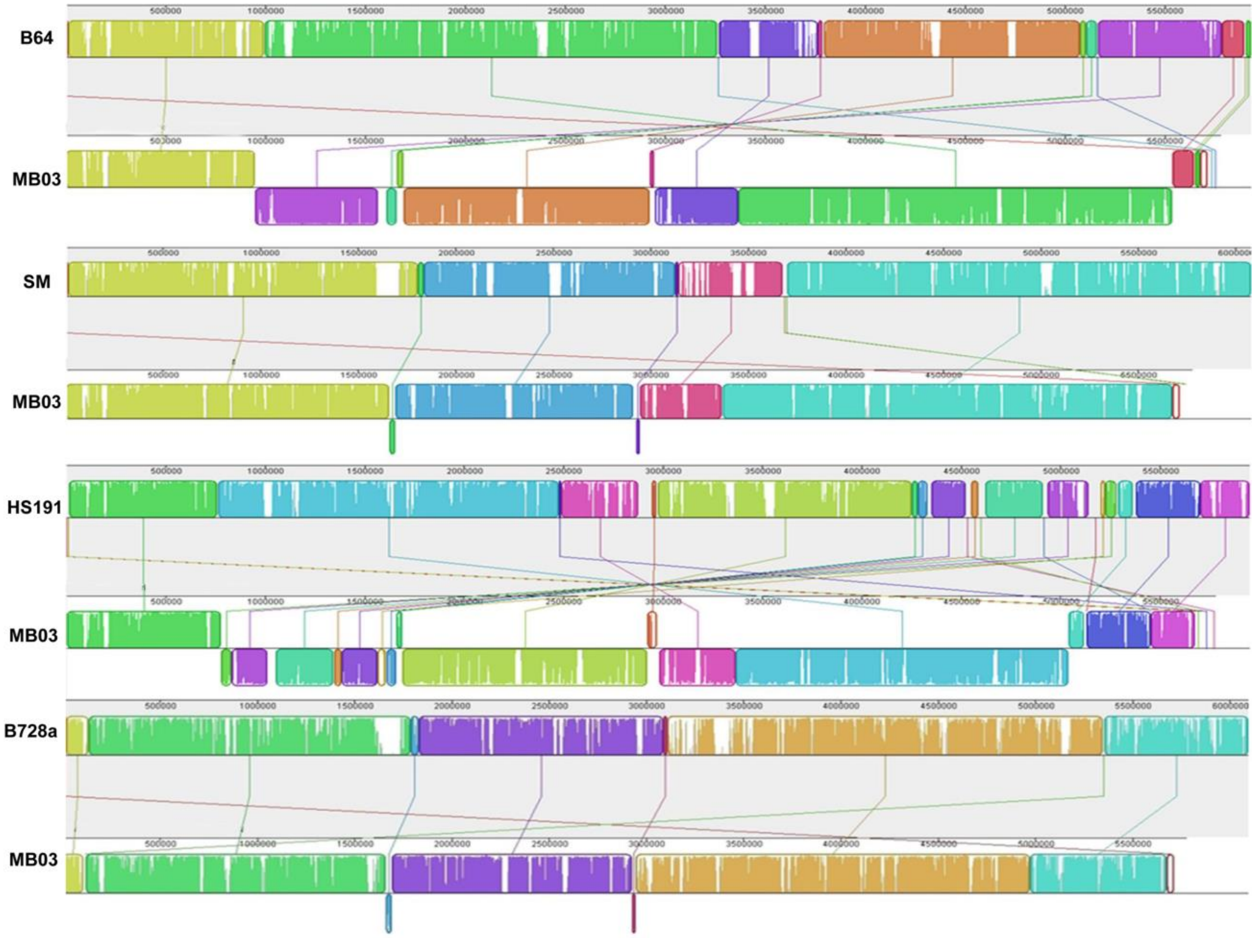

Figure S2. Genome alignment of *P. syringae* MB03 with other strains. The genome of *P. syringae* MB03 was aligned with other strains including *P. syringae* pv. *syringae* B64, *P. syringae* pv. *syringae* SM, *P. syringae* pv. *syringae* HS191 and *P. syringae* pv. *syringae* B728a. Before genomic alignment, the contigs of MB03 were ordered using Mauve. In each sub-figure, the upper colored line represents the reference genome and the lower one represents the MB03 genome. Colorless regions show unique features of the respective genomes. The numerical values on top of each panel represents nucleotide coordinates. Connecting lines are inferring synteny between two genome assemblies.
