# Supplementary material for: Identification of the Genes of the Plant Pathogen Pseudomonas syringae MB03 Required for the Nematicidal Activity Against Caenorhabditis elegans Through an Integrated Approach
Source: Front Microbiol. 2022 Mar 9;13:826962. doi: 10.3389/fmicb.2022.826962 (PMC8959697; doi:10.3389/fmicb.2022.826962)
Supplement: Supplementary file 13 [file Data_Sheet_13.pdf]

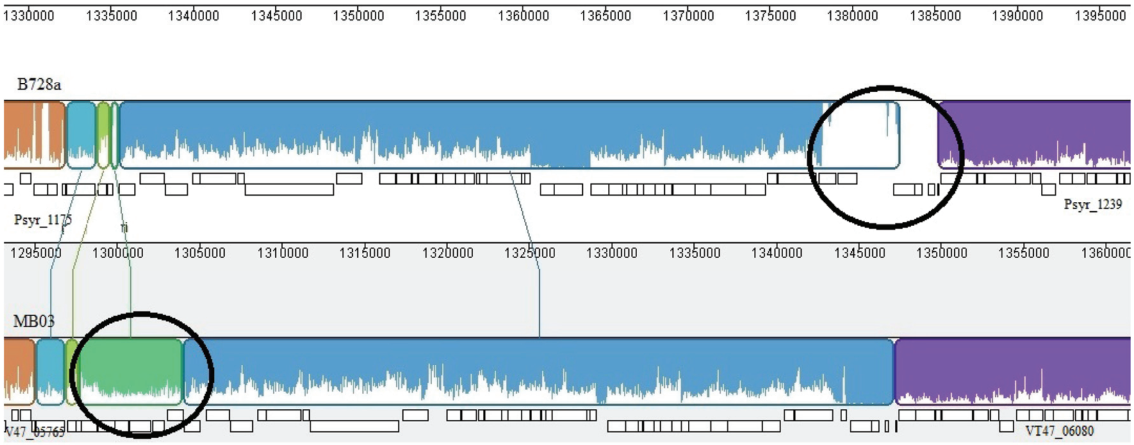

Figure S3. Comparison of candidate pathogenicity island of B728a and MB03 carrying genes for type III secretion system. The upper colored panel shows genomic organization of *P. syringae* pv. *syringae* B728a and lower panel represents genome of *P. syringae* MB03. White rectangular blocks below colored panel represents CDS of their respective genome. The regions of high dissimilarity are shown within black circles.
