# Supplementary figures and images for: Identification of the Genes of the Plant Pathogen Pseudomonas syringae MB03 Required for the Nematicidal Activity Against Caenorhabditis elegans Through an Integrated Approach
Source: Front Microbiol. 2022 Mar 9;13:826962. doi: 10.3389/fmicb.2022.826962 (PMC8959697; doi:10.3389/fmicb.2022.826962)

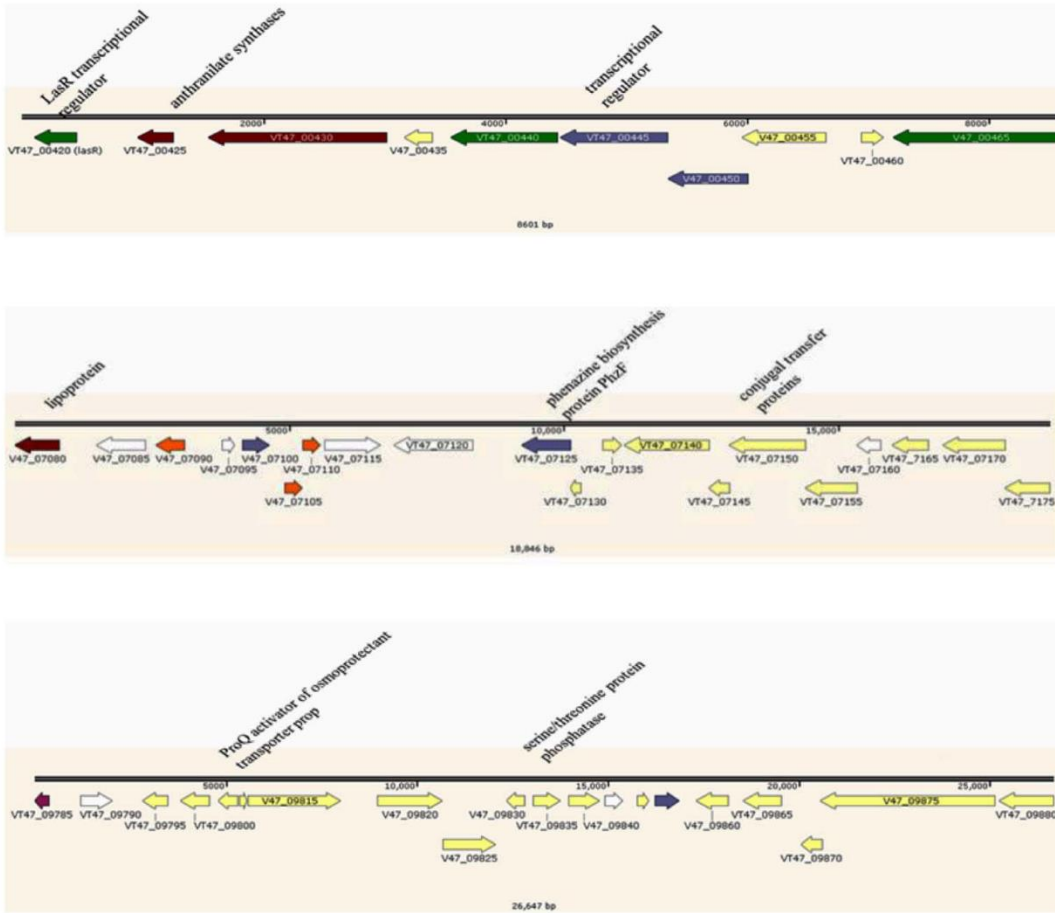

**Figure S4.** Some of the predicted genomic islands of *P. syringae* MB03

Supplement: Supplementary file 14 [file Data_Sheet_14.PDF]

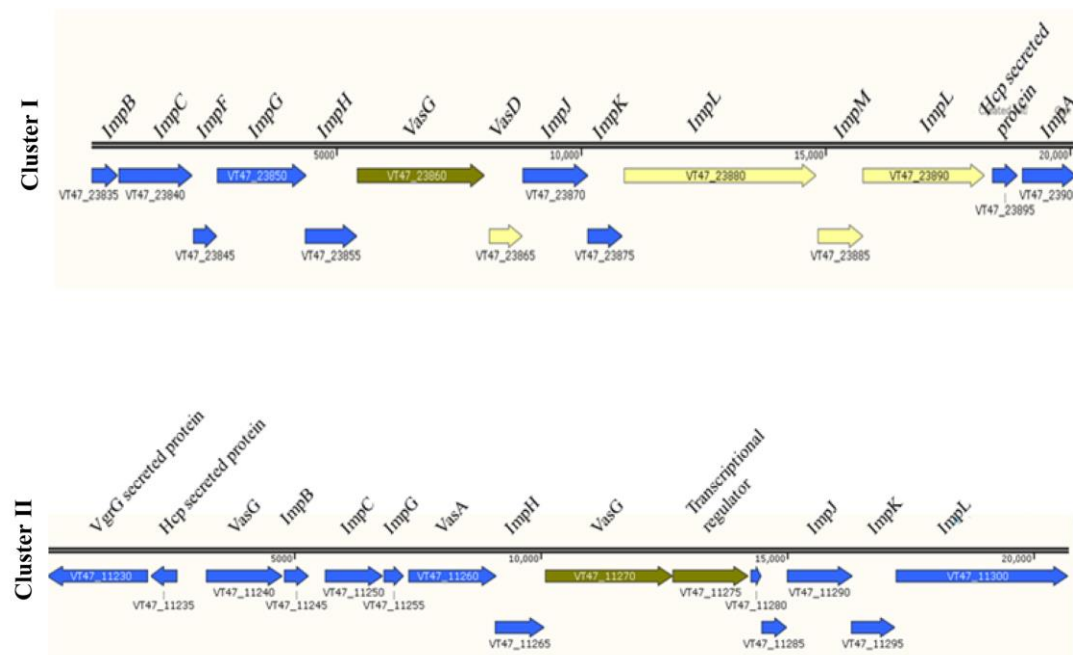

**Figure S5.** Clusters of type VI secretion system in *P. syringae* MB03

Supplement: Supplementary file 15 [file Data_Sheet_15.PDF]
